# National and Regional Trends in the Prevalence of Hypertension in South Korea Amid the Pandemic, 2009-2022: Nationwide Study of Over 3 Million Individuals

**DOI:** 10.2196/51891

**Published:** 2024-07-30

**Authors:** Hyeri Lee, Minji Kim, Selin Woo, Jaeyu Park, Hyeon Jin Kim, Rosie Kwon, Ai Koyanagi, Lee Smith, Min Seo Kim, Guillermo F López Sánchez, Elena Dragioti, Jinseok Lee, Hayeon Lee, Masoud Rahmati, Sang Youl Rhee, Jun Hyuk Lee, Ho Geol Woo, Dong Keon Yon

**Affiliations:** 1 Center for Digital Health Medical Science Research Institute Kyung Hee University College of Medicine Seoul Republic of Korea; 2 Department of Regulatory Science Kyung Hee University Seoul Republic of Korea; 3 Research and Development Unit Parc Sanitari Sant Joan de Deu Barcelona Spain; 4 Centre for Health, Performance and Wellbeing Anglia Ruskin University Cambridge United Kingdom; 5 Cardiovascular Disease Initiative Broad Institute of MIT and Harvard Cambridge, MA United States; 6 Division of Preventive Medicine and Public Health Department of Public Health Sciences, School of Medicine University of Murcia Murcia Spain; 7 Pain and Rehabilitation Centre, Department of Medical and Health Sciences Linköping University Linköping Sweden; 8 Research Laboratory Psychology of Patients, Families & Health Professionals Department of Nursing, School of Health Sciences University of Ioannina Ioannina Greece; 9 Department of Biomedical Engineering Kyung Hee University Yongin Republic of Korea; 10 Department of Physical Education and Sport Sciences Faculty of Literature and Humanities Vali-E-Asr University of Rafsanjan Rafsanjan Iran; 11 Department of Physical Education and Sport Sciences Faculty of Literature and Human Sciences Lorestan University Khoramabad Iran; 12 Research Centre on Health Services and Quality of Life Aix Marseille University Marseille France; 13 Department of Endocrinology and Metabolism Kyung Hee University School of Medicine Seoul Republic of Korea; 14 Health and Human Science University of Southern California Los Angeles, CA United States; 15 Department of Neurology Kyung Hee University Medical Center Kyung Hee University College of Medicine Seoul Republic of Korea; 16 Department of Pediatrics Kyung Hee University College of Medicine Seoul Republic of Korea

**Keywords:** COVID-19, pandemic, Korea, hypertension, HPN, high blood pressure, prevalence, national trends, regional trends, nationwide study, socioeconomic, trends, participant, population based, cross-sectional study, treatment

## Abstract

**Background:**

Understanding the association between hypertension prevalence and socioeconomic and behavioral variables during a pandemic is essential, and this analysis should extend beyond short-term trends.

**Objective:**

This study aims to examine long-term trends in the prevalence of participants diagnosed with and receiving treatment for hypertension, using data collected by a nationally representative survey from 2009 to 2022, which includes the COVID-19 pandemic era.

**Methods:**

A nationwide, population-based, cross-sectional study used data collected from the South Korea Community Health Survey between 2009 and 2022. The study sample comprised 3,208,710 Korean adults over a period of 14 years. We aimed to assess trends in the prevalence of participants diagnosed with and receiving treatment for hypertension in the national population from 2009 to 2022, with a specific focus on the COVID-19 pandemic, using weighted linear regression models.

**Results:**

Among the included 3,072,546 Korean adults, 794,239 (25.85%) were aged 19-39 years, 1,179,388 (38.38%) were aged 40-59 years; 948,097 (30.86%) were aged 60-79 years, and 150,822 (4.91%) were aged 80 years or older. A total of 1,426,379 (46.42%) were men; 761,896 (24.80%) and 712,264 (23.18%) were diagnosed with and received treatment for hypertension, respectively. Although the overall prevalence over the 14-year period increased, the upward trends of patients diagnosed with and receiving treatment for hypertension decreased during the COVID-19 pandemic era compared with the prepandemic era (β difference for trend during vs before the pandemic –.101, 95% CI –0.107 to –0.094 vs –.133, 95% CI –0.140 to –0.127). Notably, the trends in prevalence during the pandemic were less pronounced in subgroups of older adults (≥60 years old) and individuals with higher alcohol consumption (≥5 days/month).

**Conclusions:**

This nationwide representative study found that the national prevalence of participants diagnosed with and receiving treatment for hypertension increased during the prepandemic era. However, there was a marked decrease in these trends during the prepandemic era, compared with the pandemic era, particularly among specific subgroups at increased risk of negative outcomes. Future studies are needed to evaluate the factors associated with changes in the prevalence of hypertension during the COVID-19 pandemic.

## Introduction

Hypertension continues to pose a significant public health challenge globally, linked to severe conditions such as stroke, ischemic heart disease, other vascular diseases, and kidney failure, resulting in approximately 8.5 million deaths annually [1]. Given that individuals with hypertension can benefit from lifestyle changes and may require pharmacological treatment to mitigate the risk of chronic diseases and mortality, early detection and management of hypertension are crucial [2]. Since its emergence in Wuhan, China, the COVID-19 pandemic caused by SARS-CoV-2 has evolved into a global crisis profoundly affecting lives worldwide [3]. One study during the pandemic noted a roughly 25% increase in the prevalence of hypertension [4]. Despite a slowdown in the rise of hypertension prevalence, its rate continued to steadily increase during the pandemic period in South Korea [5]. Because of the interaction between COVID-19 and angiotensin-converting enzyme 2 (ACE2), there is speculation that COVID-19’s pathogenesis could be linked to hypertension [6,7]. Several reports have noted that individuals who contract COVID-19 may have a higher likelihood of developing hypertension compared with those who have not contracted the virus [8,9]. Preclinical studies have suggested that SARS-CoV-2 could affect blood pressure regulation through ACE2 receptor interaction, sustained activation of the renin-angiotensin-aldosterone system, and endothelial injury [10,11]. Previous studies have indicated that individuals aged over 60 years, males, and those with lower education and income levels exhibit a higher prevalence of hypertension among patients with COVID-19 [12,13]. However, these reports have primarily focused on short-term trends and the pathophysiological associations of hypertension prevalence. Given that hypertension is influenced by a myriad of factors, including age, sex, racial groups, socioeconomic status, health behaviors, and environmental and genetic factors [14-16], it is crucial to explore the relationship between hypertension prevalence and these factors during the pandemic, moving beyond short-term trends. Furthermore, global health care systems shifted their focus predominantly toward the detection, containment, and treatment of COVID-19, resulting in reduced emphasis on routine blood pressure screening and hypertension management in primary care settings [17].

Therefore, this study aims to examine national trends in hypertension prevalence and treatment, analyzing various risk factors before and during the COVID-19 pandemic to offer a comprehensive perspective on its public health impacts. To test our hypothesis, we utilized data from a large-scale population-based longitudinal study conducted over 14 years, from 2009 to 2022.

## Methods

### Data Source

This annual, interviewer-administered, serial cross-sectional study utilized data from the Korea Community Health Survey (KCHS) to compare the prevalence of hypertension between the periods before (2009-2019) and during the COVID-19 pandemic (2020-2022) [18]. The KCHS is a large-scale population-based survey initiated by the Korea Disease Control and Prevention Agency (KDCA) in 2008 to assess public health promotion programs [18]. It uses a stratified multistage probability sampling design to ensure that the selected households represent the demographic diversity of the South Korean population. Trained interviewers conducted face-to-face interviews to collect data on health behaviors, body measurements, and health-related outcomes, thereby minimizing response bias that could arise from self-administered questionnaires.

To accurately capture and analyze the impact of the COVID-19 pandemic, which began in early 2020, on the prevalence and treatment of hypertension, it was essential to assess changes annually. The pandemic led to substantial disruptions in health behaviors, access to medical care, and public health policies, all of which likely influenced the management and diagnosis of hypertension. Presenting data annually enables a thorough investigation into the temporal effects of the pandemic and the influence of specific health policies on trends in hypertension.

Sampling was conducted using proportional sampling, taking into account the number of households based on housing types, with a secondary sample of households selected through systematic sampling [19]. Potential participants were contacted directly by the South Korean government via postal mail. Each year, a sample size of 900 individuals per region was surveyed from a population of 220,000 individuals, aiming to represent the broader population of South Korea. The KCHS applied a proportional sampling method to select participants, targeting adults aged 19 years and older. Data used in the study were deidentified.

### Ethics Approval

The study protocol received approval from the Institutional Review Board of the KDCA (protocol numbers 2010-02CON-22-P, 2011-05CON04-C, 2012-07CON-01-2C, 2013-06EXP-01-3C, 2014-08EXP-09-4CA, and 2016-10-01-TA), as well as approval under the local law of state-approved statistics (approval number 117075) and the Enforcement Regulation (Article 2, Paragraph 2, Item 1) of the Bioethics and Safety Act by the Korean government. All participants provided written informed consent, and this study adhered to the principles outlined in the Declaration of Helsinki.

### Independent Variable

**Figure 1:**
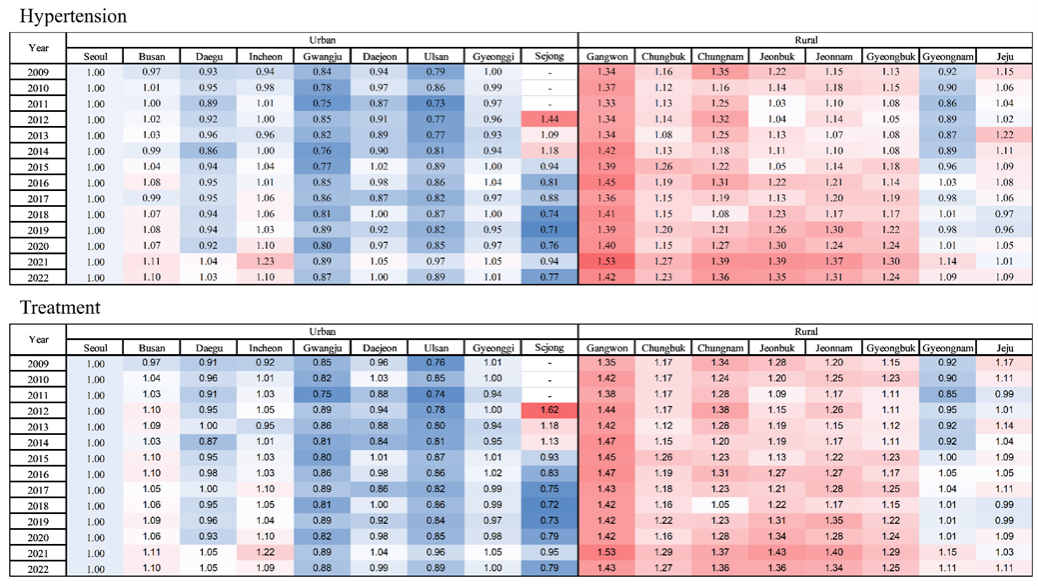
Regional relative prevalence ratio of (A) hypertension (upper panels) and (B) hypertension treatment (lower panels) in urban and rural regions (reference: Seoul) during the period from 2009 to 2022.

**Figure 2:**
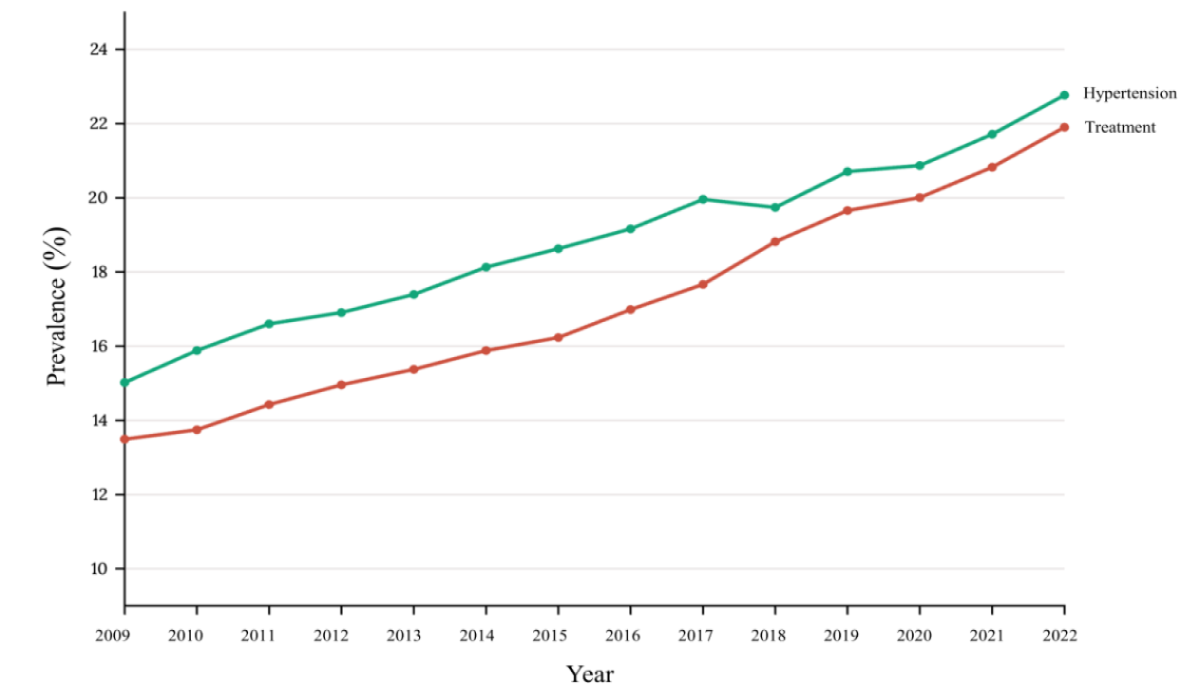
Nationwide trends in the overall prevalence of hypertension among Korean adults during the period from 2009 to 2022.

In this study, the independent variable was defined as the survey period, divided annually for more detailed analysis: 2009, 2010, 2011, 2012, 2013, 2014, 2015, 2016, 2017, 2018, 2019, 2020, 2021, and 2022 (Figures 1 and 2). This decision was made to improve clarity and precision in observing trends over time. Additionally, further analysis was segmented into 5 periods: 2009-2011, 2012-2014, 2015-2017, 2018-2019, and 2020-2022 (Tables S1 and S2 in Multimedia Appendix 1) [20]. Given that the first case of COVID-19 in South Korea was reported in 2020, the period from 2020 to 2022 was designated as the pandemic period.

### Dependent Variable

Patients diagnosed with hypertension by a physician were classified into the hypertension group. Treatment status among these patients was determined based on their response to the question: “Are you currently receiving treatment to manage your blood pressure?” Those who reported currently taking medication for blood pressure management or using nondrug therapies such as exercise and dietary changes after diagnosis were categorized as the treatment group [21].

### Covariates

We considered 12 covariates related to participant characteristics: age (19-39, 40-59, 60-69, and ≥80 years), sex, region of residence (urban and rural) [22,23], recipients of basic livelihood security [24], economic level of the family (unknown, low, and high) [15], smoking status [25], frequency of alcohol consumption (0, <5, and ≥5 days/month) [26], BMI category (normal and overweight to obese) [27], depression status [28], educational background (high school or lower and college or higher) [29], occupation status (white-collar, blue-collar, and unemployed) [30], and marital status (married and unmarried) [31]. The economic level of the family was categorized based on monthly household income into 3 groups: low (<3 million won [<US $2176]), high (≥3 million won [>US $2176]), and unknown [32,33]. Residential areas were classified into 2 categories [34]: urban (including Seoul, Busan, Daegu, Incheon, Gwangju, Daejeon, Ulsan, Sejong, and Gyeonggi) and rural (including Gangwon, Chungbuk, Chungnam, Jeonbuk, Jeonnam, Gyeongbuk, Gyeongnam, and Jeju). Occupational variables were categorized into the following 3 groups according to the Korean Standard Occupational Classification: white-collar (including managers, specialists, and secretaries), blue-collar (including service workers, salespeople, agricultural workers, forestry workers, fishermen, craftsmen, artists, machine operators, unskilled workers, and armed forces), and unemployed (including students and housewives). According to the Korean Society for the Study of Obesity [35], BMI was calculated based on self-reported height and body weight and categorized into 2 groups: normal (<23.0 kg/m^2^) and overweight to obese (≥23.0 kg/m^2^).

### Statistical Analysis

In this study, we examined trends in the prevalence of hypertension diagnosis and treatment among the national population from 2009 to 2022, with a specific emphasis on the COVID-19 pandemic period. To accurately account for the demographic composition of the population and mitigate potential sampling biases, all statistical analyses were performed using weights derived from the stratified multistage sampling design of the KCHS.

We used weighted linear regression models to assess changes in hypertension prevalence trends from the prepandemic period (2009 to 2019) to the pandemic period (2020 to 2022). This approach was selected for its capability to analyze data from complex sampling designs such as the KCHS, where stratified multistage sampling may introduce correlations within strata. To address the clustering and correlation inherent in survey data from the KCHS, adjustments for design effects such as robust standard errors or design-based weights were applied. To quantify changes, we calculated the β difference between the 2 periods.

Similarly, weighted logistic regression models were used to estimate odds ratios (ORs) and 95% CIs to identify risk factors associated with hypertension and its treatment during the pandemic period compared with the earlier timeframe. Covariates included in the models were age, sex, region of residence, receipt of basic livelihood security, economic level of the family, smoking status, frequency of alcohol consumption, BMI category, depression status, educational background, occupation status, and marital status. These models facilitated a comprehensive analysis by accounting for potential confounders and elucidating associations within the population data. Subgroup analyses were also conducted within these logistic regression models to explore demographic and risk-stratified trends. This approach enabled the identification of specific groups showing statistically significant associations in hypertension prevalence during the pandemic, as well as the assessment of the impact of various risk factors.

Our goal was to pinpoint groups demonstrating notable shifts in hypertension prevalence attributable to the pandemic and to evaluate the influence of diverse risk factors. All analyses were executed using SAS software (version 9.4; SAS Institute), with statistical significance set at *P* values less than .05 [36,37].

## Results

The study sample initially included 3,208,710 adults who participated in the KCHS survey over a 14-year period. After excluding 136,164 participants due to missing data on health-related outcomes and BMI, the final analysis included data from 3,072,546 participants (761,896 with hypertension; 712,264 receiving treatment; and 2,310,650 in the control group; see Figure S1 in Multimedia Appendix 1). The demographic characteristics of the participants are detailed in Table S1 in Multimedia Appendix 2 [32,33]. Among the 3,072,546 adult participants, 794,239 (25.85%) were aged 19-39 years, 1,179,388 (38.38%) were aged 40-59 years, 948,097 (30.86%) were aged 60-79 years, and 150,822 (4.91%) were aged 80 years or older. Additionally, 1,426,379 (46.42%) were male participants and 1,560,286 (50.78%) were rural residents (see Figures S2-S13 in Multimedia Appendix 1).

Table S2 in Multimedia Appendix 2 presents the prevalence of diagnosed hypertension over a 14-year period and its trends before and during the COVID-19 pandemic. From 2009 to 2022, the prevalence of diagnosed hypertension increased from 15.02% (95% CI 14.81%-15.22%) to 22.77% (95% CI 22.51%-23.03%; Figure S14 and Table S3 in Multimedia Appendix 1). Despite the overall increase, the rate of hypertension prevalence growth slowed during the COVID-19 pandemic. Specifically, the growth rate decreased from a β of .124 (95% CI 0.119-0.130) before the pandemic to a β of .024 (95% CI 0.020-0.027) during the pandemic, indicating a substantial slowdown in new diagnoses (β difference –.101, 95% CI –0.107 to –0.094; Table S3 in Multimedia Appendix 2). This trend was particularly notable among older adults (aged ≥60 years) and frequent alcohol consumers (≥5 days/month), where the increase in hypertension diagnoses not only slowed but actually reversed, indicating a decrease in new cases during the pandemic.

Similarly, the treatment rate for hypertension increased from 13.49% (95% CI 13.30%-13.68%) in 2009 to 21.90% (95% CI 21.65%-22.15%) in 2022 (Table S2 in Multimedia Appendix 2). However, akin to diagnoses, the increase in treatment rates also experienced a downturn during the pandemic, with the growth rate decreasing (β difference –.133, 95% CI –0.140 to –0.127; Table S3 in Multimedia Appendix 2). The β values, which signify the annual change in prevalence rates, reveal that while there was a consistent annual increase in cases before the pandemic, the rate of increase substantially slowed during the pandemic. This slowdown reflects disruptions likely caused by reduced access to health care services and changes in patient behavior.

The continuous increase in both hypertension diagnoses and treatment over the study period, albeit at a slower pace during the pandemic, highlights a persistent trend of growing hypertension burden. This trend, alongside the observed slowdown in the rate of increase during the pandemic, underscores the substantial impact of the pandemic on health service utilization and hypertension management. Detailed trends and statistical significance of these observations are presented in Tables S2 and S3 in Multimedia Appendix 2, with stratification analysis revealing consistent patterns across various demographic groups. This indicates a broad and significant impact of the pandemic on hypertension management across the population.

## Discussion

### Principal Findings

This study analyzed trends in the prevalence of participants diagnosed with and receiving treatment for hypertension during the COVID-19 pandemic era compared with the prepandemic era, utilizing nationwide representative survey data from over 3 million South Koreans from 2009 to 2022. To the best of our knowledge, this is the first comprehensive, long-term study using a data set that covers pandemic periods to investigate trends in hypertension. During the prepandemic era, both the number of participants diagnosed with hypertension and those receiving treatment consistently increased, as evidenced by each variable. Interestingly, the data revealed a deceleration in the upward trend of hypertension diagnoses and treatment prevalence during the pandemic era compared with the prepandemic period. Furthermore, the estimated prevalence of participants diagnosed with hypertension decreased during the pandemic era, particularly among specific groups such as older adults (aged ≥60 years) and those who frequently consumed alcohol (≥5 days/month). Similarly, the estimated prevalence of participants receiving treatment for hypertension decreased during the pandemic era, particularly among specific groups such as older adults and individuals who frequently consumed alcohol.

### Plausible Mechanism

As COVID-19 rapidly spread and lockdowns were imposed, there was a marked decline in both national and individual economic activities. Medical supply chains were disrupted, and access to medical care was significantly restricted [38]. Previous research has indicated a 22% decrease in outpatient visits and a 20% reduction in outpatient prescriptions, including antihypertensive drugs, during the early phase of the pandemic [39]. Moreover, health care inequalities worsened during the COVID-19 pandemic [40]. Consequently, the data showed a more pronounced decrease in the upward trend of hypertension diagnoses during the pandemic compared with the prepandemic era. Similarly, the upward trend in the prevalence of participants receiving hypertension treatment declined during the COVID-19 pandemic compared with the prepandemic period. Previous studies have suggested an increase in average systolic and diastolic blood pressure during the COVID-19 pandemic [41].

Hypertension in patients with COVID-19 has been linked to a 2.5-fold increase in both the severity of the disease and mortality rates [42]. It is plausible that the increased mortality rates during the COVID-19 pandemic could be linked to the observed lower rates of hypertension diagnoses [43]. Additionally, reduced accessibility to health care facilities induced by the pandemic may have affected regular health management and timely hypertension diagnosis [44]. This effect could be reflected in the prevalence statistics, potentially resulting in a decrease in overall hypertension prevalence [45].

This effect of COVID-19 has predominantly been observed in older patients [46]. Older individuals with hypertension are at an increased risk of severe COVID-19 and higher mortality rates [7]. Furthermore, it is conceivable that older individuals may have avoided hospital visits due to the risk of COVID-19 infection, potentially leading to a decrease in recorded diagnoses of hypertension [47]. Additionally, during the pandemic, many people experienced increased stress, anxiety, and social isolation, which in turn led to an uptick in alcohol consumption [48,49]. Excessive alcohol consumption can contribute to cardiac and renal damage in individuals with hypertension, potentially exacerbating mortality rates [50]. Accordingly, during the COVID-19 pandemic, higher mortality rates in vulnerable groups may correlate with the observed decrease in the overall reported prevalence of hypertension.

### Comparison With Previous Studies

A significant amount of research has focused on exploring the global prevalence and trends of hypertension. This body of research offers a robust foundation for comprehending the epidemiological landscape of this critical health issue across diverse populations and geographic regions. Recent studies have also examined the relationship between the COVID-19 pandemic and hypertension. Studies on hypertension prevalence before COVID-19 indicated an increasing trend in China (n=9,191,121) [51], India (n=1542) [52], Bangladesh (n=305,432) [53], the Democratic Republic of the Congo (n=10,866) [54], and Ethiopia (n=66,099) [55]. Conversely, Brazil showed constant trends (n=578,977) [56], while Italy exhibited a decreasing trend (n=88,823) [57]. Furthermore, studies on the prevalence of hypertension during COVID-19 revealed an increasing trend in the United States (n=464,585 and n=72,706) [41,58], China (n=3724 and n=7394) [59,60], Japan (n=748) [61], Turkey (n=142) [62], Argentina (n=12,144) [63], and Brazil (n=57,768) [64]. Conversely, decreasing trends were observed in France (n=2273) [65] and Italy (n=126) [66]. Additionally, a population-based study involving 1.2 million participants across 1468 studies worldwide observed an increase in the prevalence of hypertension [67]. The studies mentioned above had smaller participant numbers compared with our study, with data excluding China, and presented varied findings regarding the prevalence of participants diagnosed with hypertension during the COVID-19 pandemic. Additionally, our study is the first to investigate the prevalence of participants receiving treatment for hypertension during the COVID-19 pandemic for the first time. Besides, while previous studies have documented the prevalence of hypertension during various periods, our study uniquely captures the immediate and enduring effects of the COVID-19 pandemic over several years. This longitudinal aspect allows us to observe not just the short-term impacts typically reported in other studies but also how these effects evolve or persist over time.

### Policy Implications

The COVID-19 pandemic appears to have exacerbated challenges in managing treated hypertension, possibly due to limited health care access for patients with hypertension resulting from enforced restrictions. Furthermore, individuals refraining from social activities during the pandemic may experience negative impacts on mental health, which could potentially lead to poorer hypertension management [44,68]. In particular, higher prevalence rates were observed among individuals at higher risk, such as older adults and those who frequently consume alcohol. Understanding these trends and identifying groups with increased prevalence are crucial for developing mental and health care programs aimed at addressing hypertension and related diseases effectively. To address these issues, the adoption of online medical services and support through smartphone apps, which has been promoted over the past few years, has significantly accelerated. These technologies hold great potential to fundamentally address these challenges, making them critically important [69,70].

### Limitations

This study had several limitations. First, it relied on self-report questionnaires to collect information about hypertension and for receiving hypertension treatment in Korean adults. This method may have led to underestimations in the prevalence of these issues due to reporting biases such as recall bias, information bias, selection bias, and the stigma effect. Second, this study did not investigate specific risk factors and comorbidities associated with hypertension. Future studies should explore factors such as physical activity, sleep patterns, dietary habits, and other environmental factors that could contribute to hypertension and its management. Understanding these associations could provide deeper insights into effective prevention and treatment strategies for hypertension. Third, it is important to note that our data specifically pertain to Korean adults, and therefore, these findings may not be generalizable to other regions or populations. Conducting large-scale international studies would be necessary to examine the factors associated with hypertension in adults across different countries and demographics. Fourth, this study acknowledges potential biases inherent in the sampling and weighting processes, which may impact the generalizability and interpretation of the results. Despite efforts to ensure representativeness, discrepancies between raw and weighted data indicate possible selection biases, suggesting that certain demographic groups might be underrepresented or overrepresented. Finally, our study is unable to report on the proportion of individuals who may be managing their hypertension through various treatments without a physician’s diagnosis, which could potentially influence the observed prevalence and treatment outcomes. Despite these limitations, this study represents the first long-term, large-scale examination of hypertension trends in South Korea using a data set of more than 3 million adults.

### Conclusion

This serial cross-sectional study observed that the national prevalence of participants diagnosed with hypertension and receiving treatment for hypertension exhibited an upward trend during the prepandemic era. However, this trend appeared to decelerate during the pandemic era. Notably, this change was more pronounced among specific subgroups, including older adults (≥60 years), frequent alcohol consumers (≥5 days/month), the unemployed, and the unmarried. Future research is warranted to explore the factors associated with the observed changes in the prevalence of hypertension across different periods of the COVID-19 pandemic.

## Appendix

Multimedia Appendix 1 National and regional trends in the prevalence of hypertension among South Korean adults during the COVID-19 pandemic.

Multimedia Appendix 2 Analysis results: (1) general characteristics of South Korean adults, 2009-2022; (2) nationwide trends in the prevalence of participants diagnosed with and receiving treatment for hypertension among South Korean adults, 2009-2022; and (3) nationwide trends in the prevalence of participants diagnosed with and receiving treatment for hypertension before (2009-2019) and during the COVID-19 pandemic (2020-2022) among South Korean adults.

## Abbreviations

ACE2: angiotensin-converting enzyme 2
KCHS: Korea Community Health Survey
KDCA: Korea Disease Control and Prevention Agency

## Data Availability

Data are available on reasonable request. The study protocol and statistical code can be obtained from DKY. The data set is available from the Korea Disease Control and Prevention Agency (KDCA) through a data use agreement.
