# Supplementary material for: National and Regional Trends in the Prevalence of Hypertension in South Korea Amid the Pandemic, 2009-2022: Nationwide Study of Over 3 Million Individuals
Source: JMIR Public Health Surveill. 2024 Jul 30;10:e51891. doi: 10.2196/51891 (PMC11322715; doi:10.2196/51891)
Supplement: Multimedia Appendix 1 [file publichealth_v10i1e51891_app1.docx]

| **Supplementary Material** |
| --- |

Original Article

**National and regional trends in the prevalence of hypertension in South Korea amid the pandemic, 2009–2022: a nationwide study of over 3 million individuals**

**Subtitle:** Prevalence of hypertension

Hyeri Lee,^1,2∥^ Minji Kim,^1,2∥^ Selin Woo,^1^ Jaeyu Park,^1,2^ Hyeon Jin Kim,^1,2^ Rosie Kwon,^1^ Ai Koyanagi,^3^ Lee Smith,^4^ Min Seo Kim,^5^ Guillermo F. López Sánchez,^6^ Elena Dragioti,^7,8^ Jinseok Lee,^9^ Hayeon Lee,^1,9^ Masoud Rahmati,^10,11^ Sang Youl Rhee,^12^ Jun Hyuk Lee,^13^ Ho Geol Woo,^14^* Dong Keon Yon, ^1,2,15^*

^∥^ The authors were contributed equally

*Corresponding authors

**Corresponding author**

Dong Keon Yon, MD, PhD, FACAAI, FAAAAI (lead contact)

Department of Pediatrics, Kyung Hee University College of Medicine, 23 Kyungheedae-ro, Dongdaemun-gu, Seoul, 02447, South Korea

Email: [yonkkang@gmail.com](mailto:yonkkang@gmail.com)

**Table of contents**

| **Supplementary Material** |  | **Page** |
| --- | --- | --- |
| **Figure S1** | Study flow | P4 |
| **Figure S2** | Age-stratification trends in prevalence of from Korean adults, 2009-2022 | P5 |
| **Figure S3** | Sex-stratification trends in prevalence of from Korean adults, 2009-2022 | P6 |
| **Figure S4** | BMI-stratification trends in prevalence of from Korean adults, 2009-2022 | P7 |
| **Figure S5** | Smoking status-stratification trends in prevalence of from Korean adults, 2009-2022 | P8 |
| **Figure S6** | Economic level of family-stratification trends in prevalence of from Korean adults, 2009-2022 | P9 |
| **Figure S7** | Regions of residence-stratification trends in prevalence of from Korean adults, 2009-2022 | P10 |
| **Figure S8** | Occupation status-stratification trends in prevalence of from Korean adults, 2009-2022 | P11 |
| **Figure S9** | Alcohol consumption frequency-stratification trends in prevalence of from Korean adults, 2009-2022 | P12 |
| **Figure S10** | Basic livelihood security recipient-stratification trends in prevalence of from Korean adults, 2009-2022 | P13 |
| **Figure S11** | Depression status-stratification trends in prevalence of from Korean adults, 2009-2022 | P14 |
| **Figure S12** | Marital status-stratification trends in prevalence of from Korean adults, 2009-2022 | P15 |
| **Figure S13** | Educational background-stratification trends in prevalence of from Korean adults, 2009-2022 | P16 |
| **Figure S14** | Trends in overall hypertension among Korean, 2009-2022 (2-3 years analysis) | P17 |
| **Table S1** | General characteristics of South Korean adults, 2009-2022 (2-3 years analysis) | P18 |
| **Table S2** | Nationwide trends in prevalence of hypertension and receiving treatment for hypertension, 2009-2022 (2-3 years analysis) | P22 |
| **Table S3** | Nationwide trends in prevalence of hypertension and receiving treatment for hypertension, 2009-2022 (unweighted) | P28 |

**Figure S1.** Study flow.

**
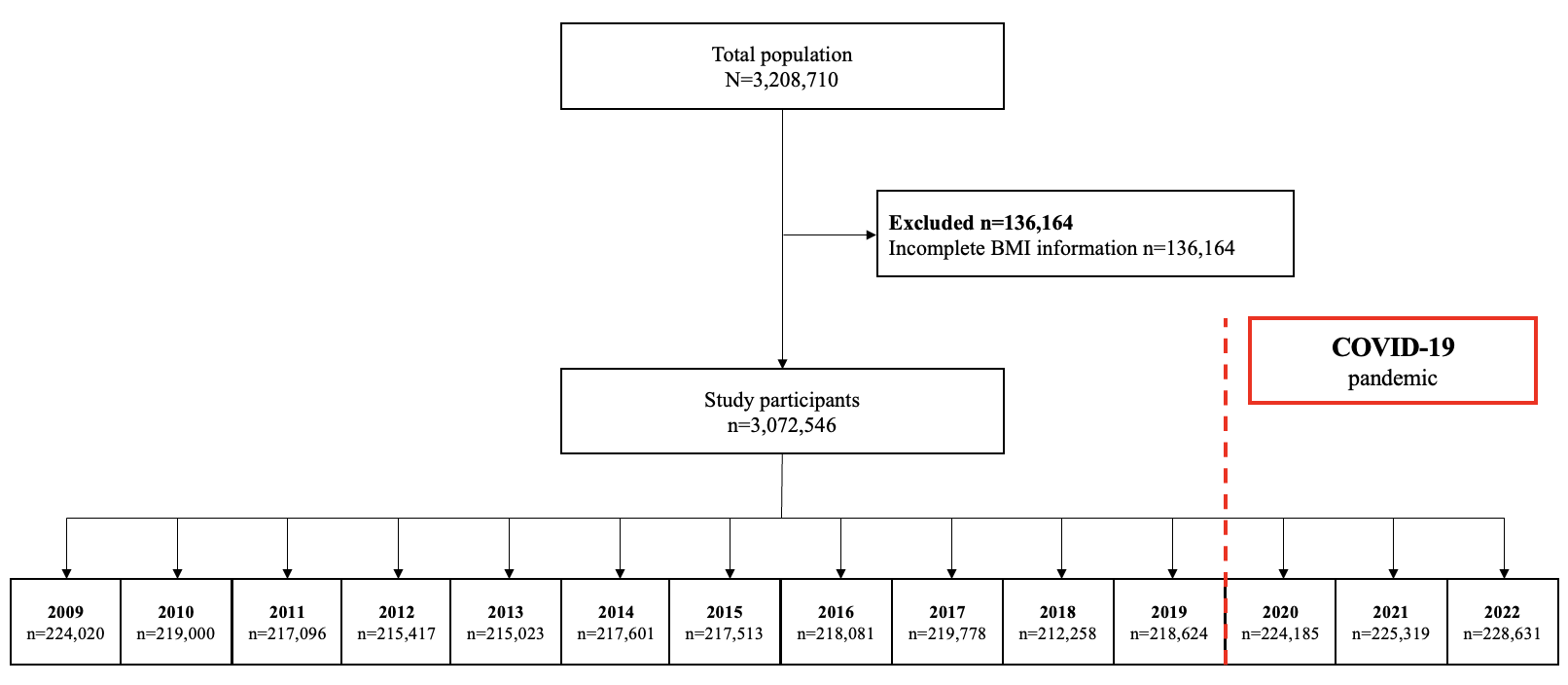
**

BMI; body mass index

**Figure S2.** Age-stratification trends in prevalence of from Korean adults, 2009-2022.

**
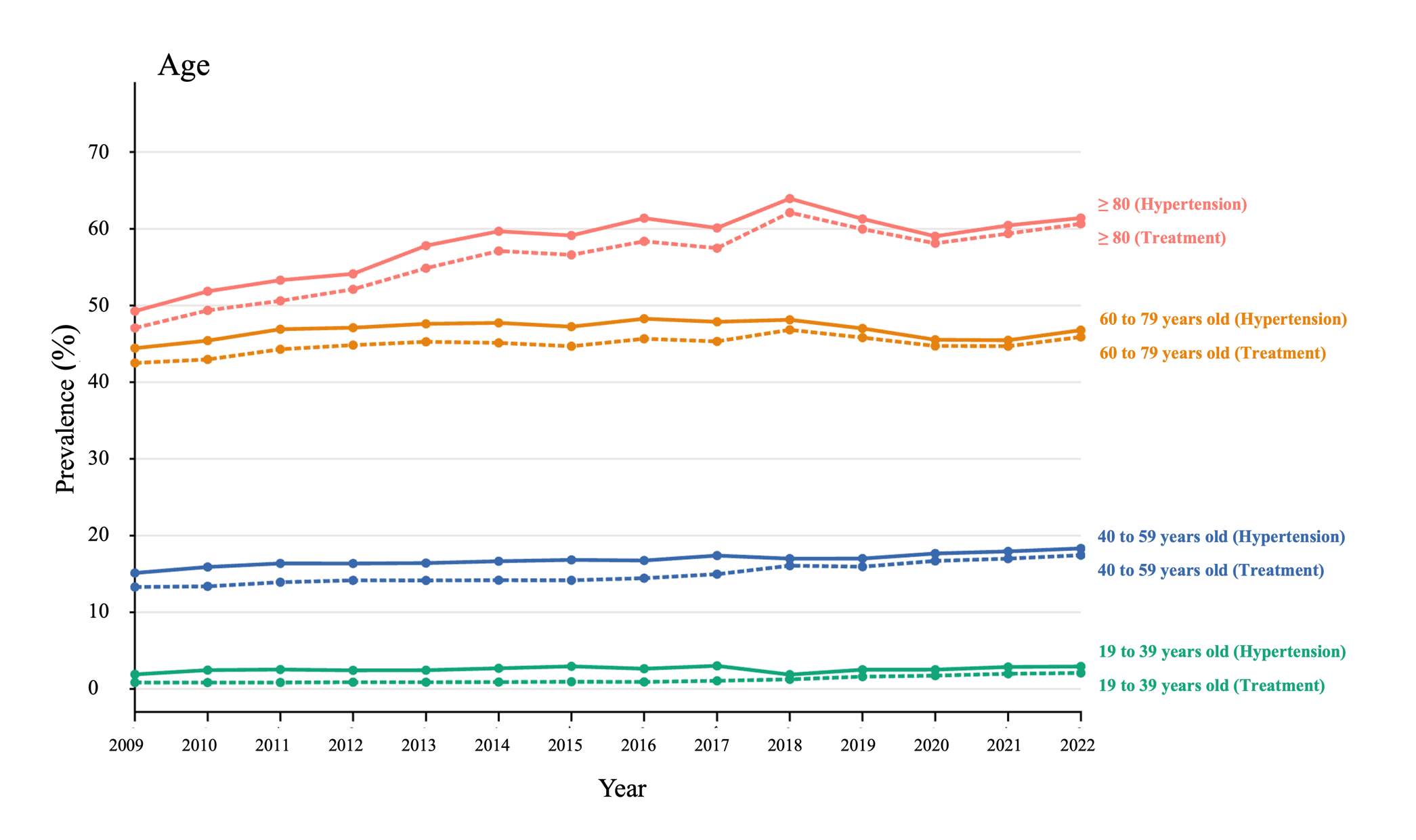
**

**Figure S3.** Sex-stratification trends in prevalence of from Korean adults, 2009-2022.


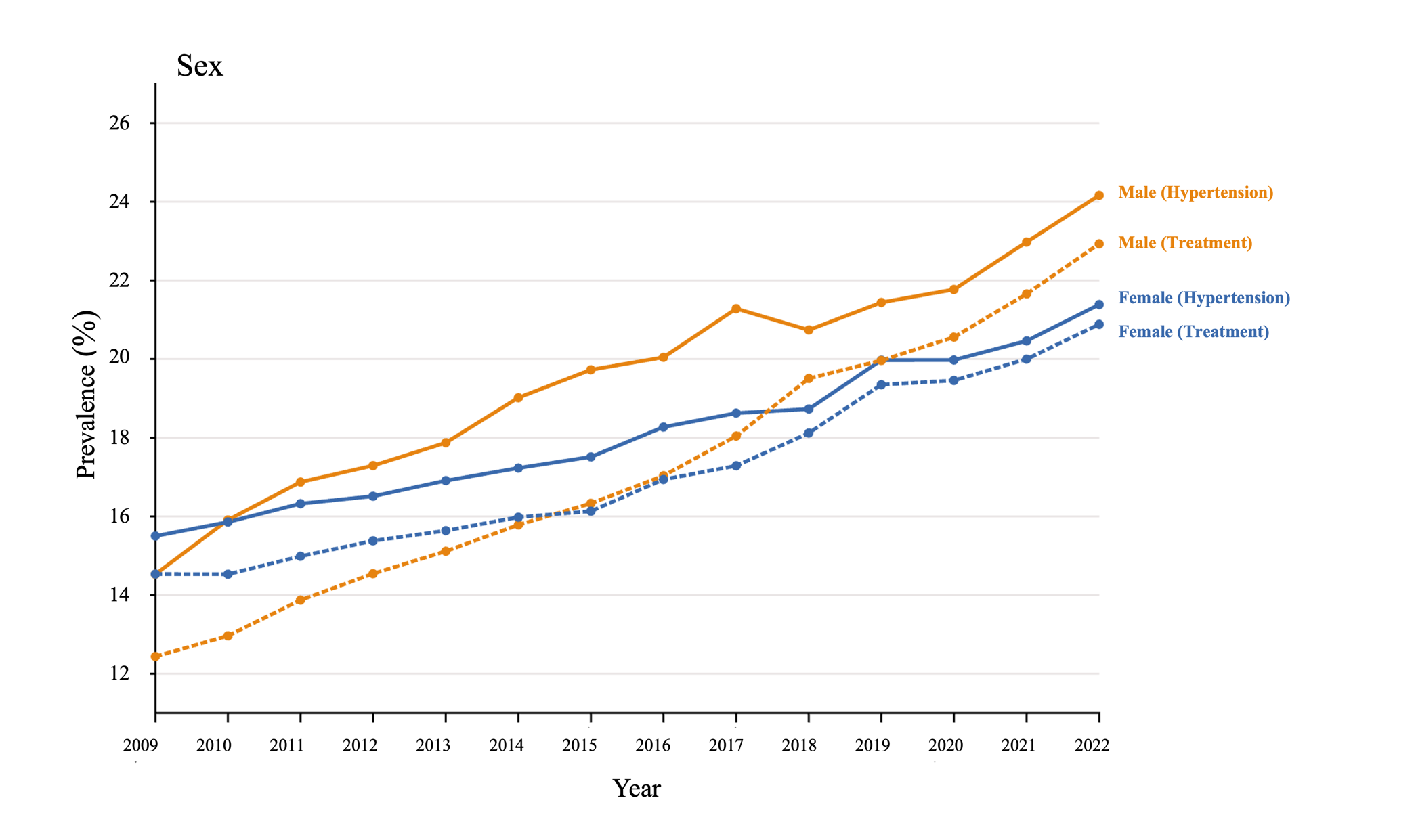


**Figure S4.** BMI-stratification trends in prevalence of from Korean adults, 2009-2022.


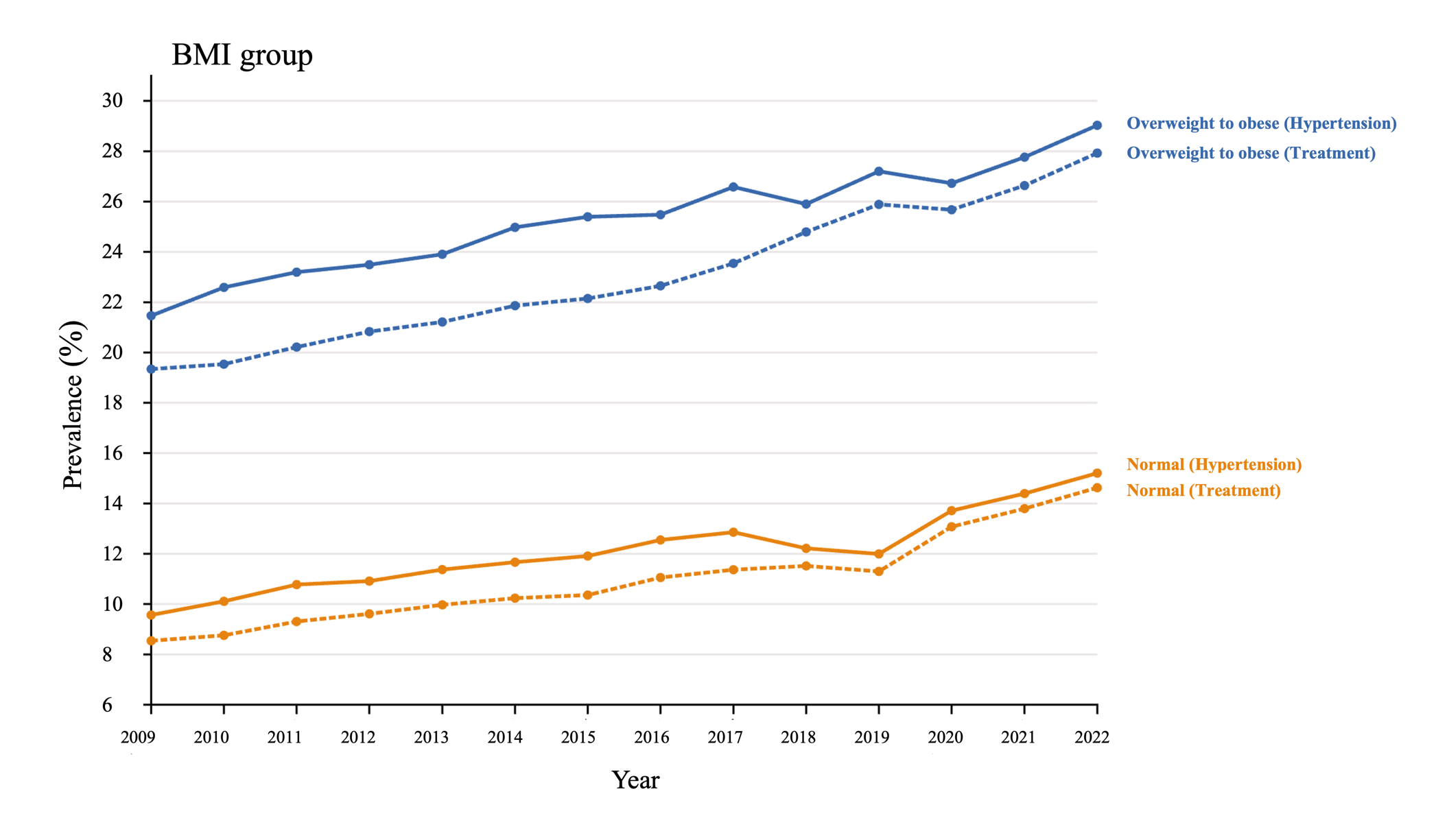


BMI; body mass index

**Figure S5.** Smoking status-stratification trends in prevalence of from Korean adults, 2009-2022.


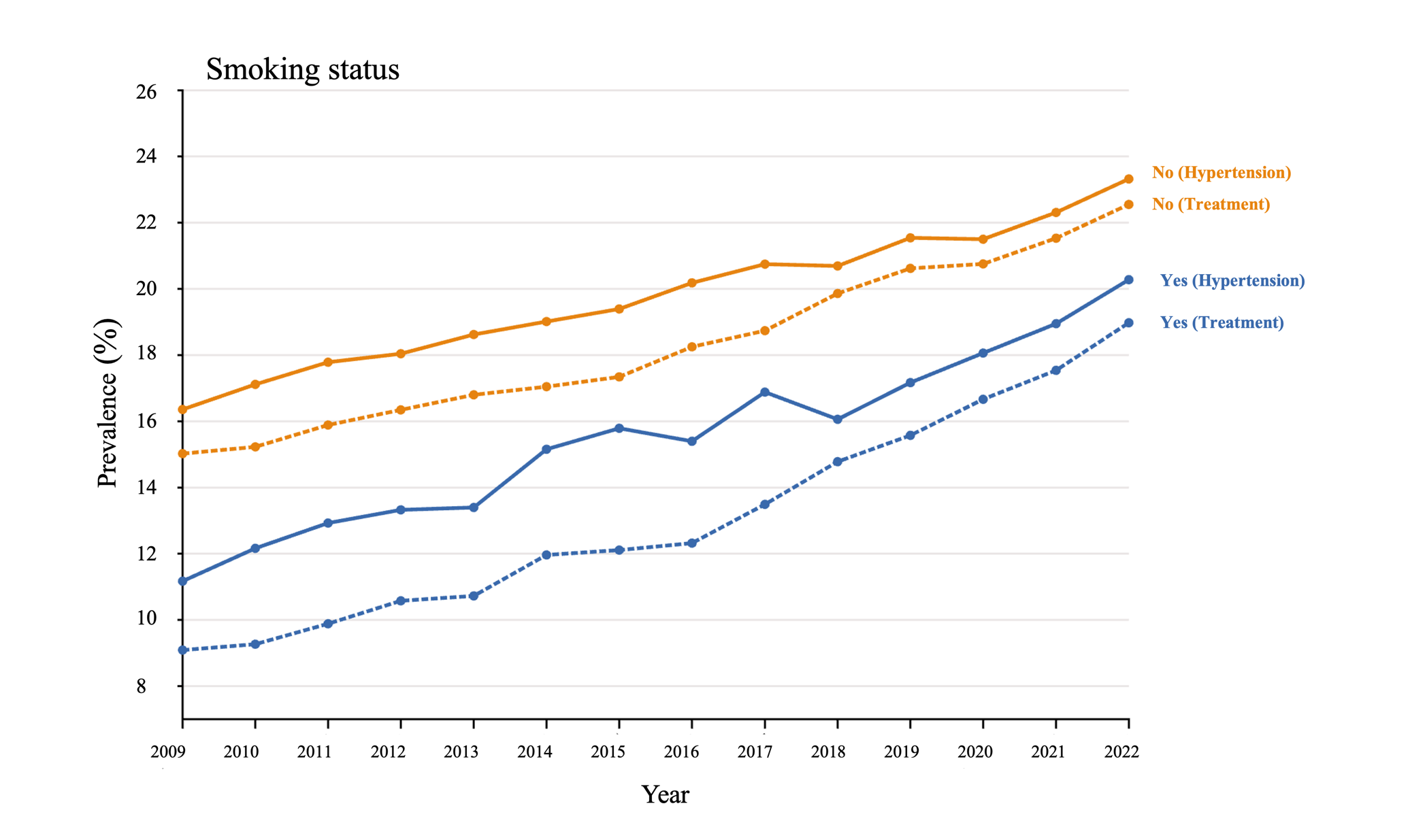


**Figure S6.** Economic level of family-stratification trends in prevalence of from Korean adults, 2009-2022.


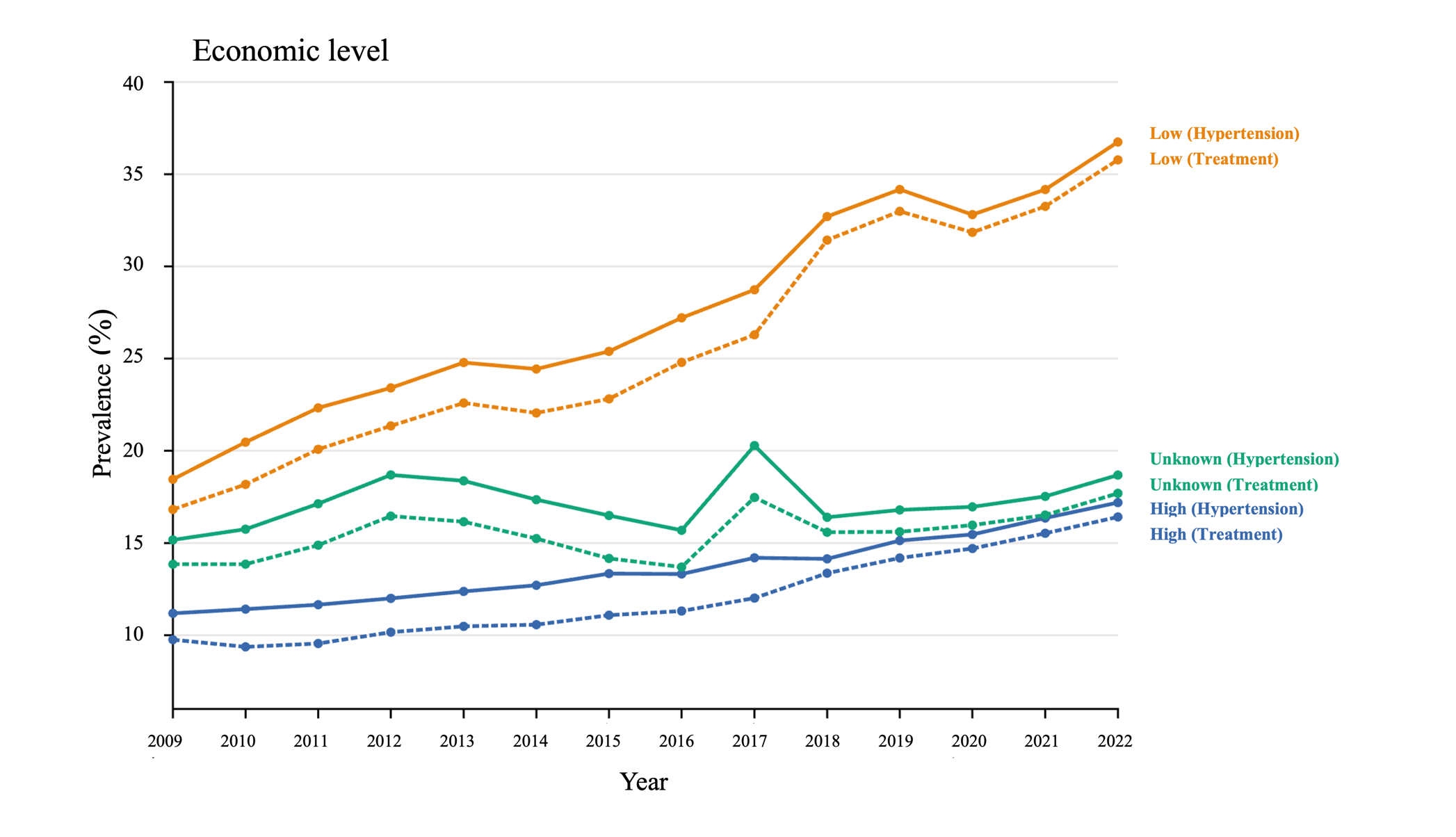


**Figure S7.** Region of residence-stratification trends in prevalence of from Korean adults, 2009-2022.


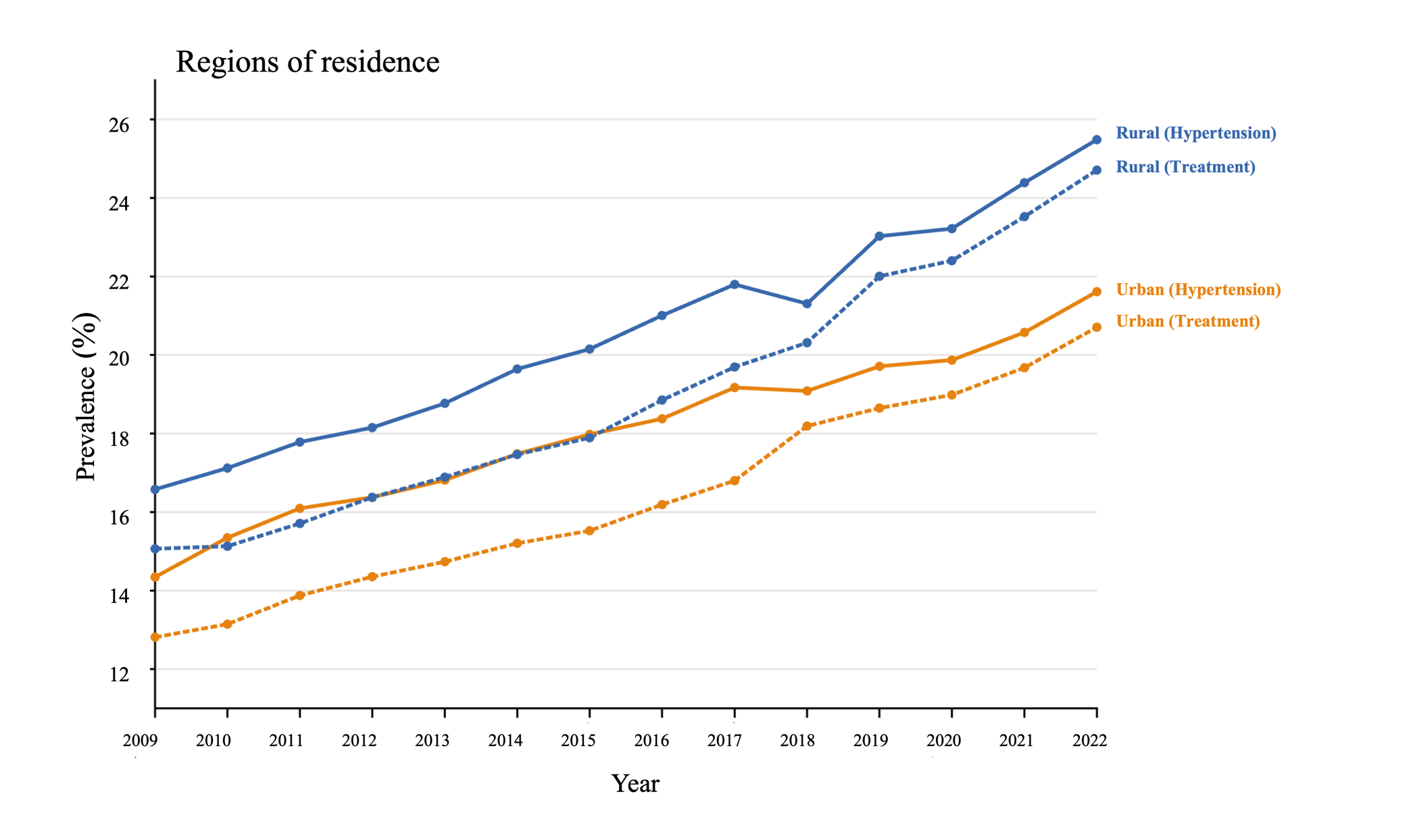


**Figure S8.** Occupation status-stratification trends in prevalence of from Korean adults, 2009-2022.


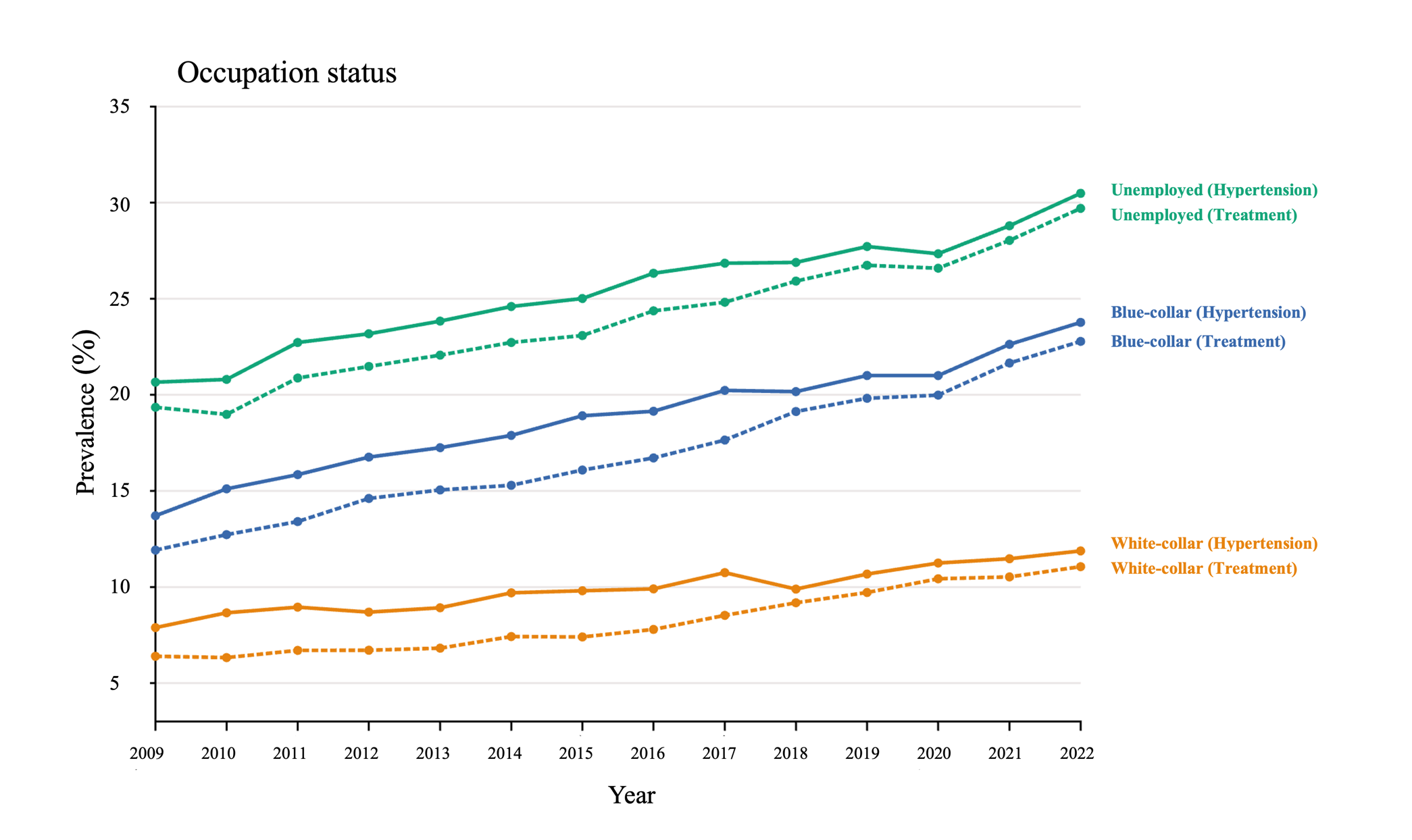


**Figure S9.** Alcohol consumption frequency-stratification trends in prevalence of from Korean adults, 2009-2022.


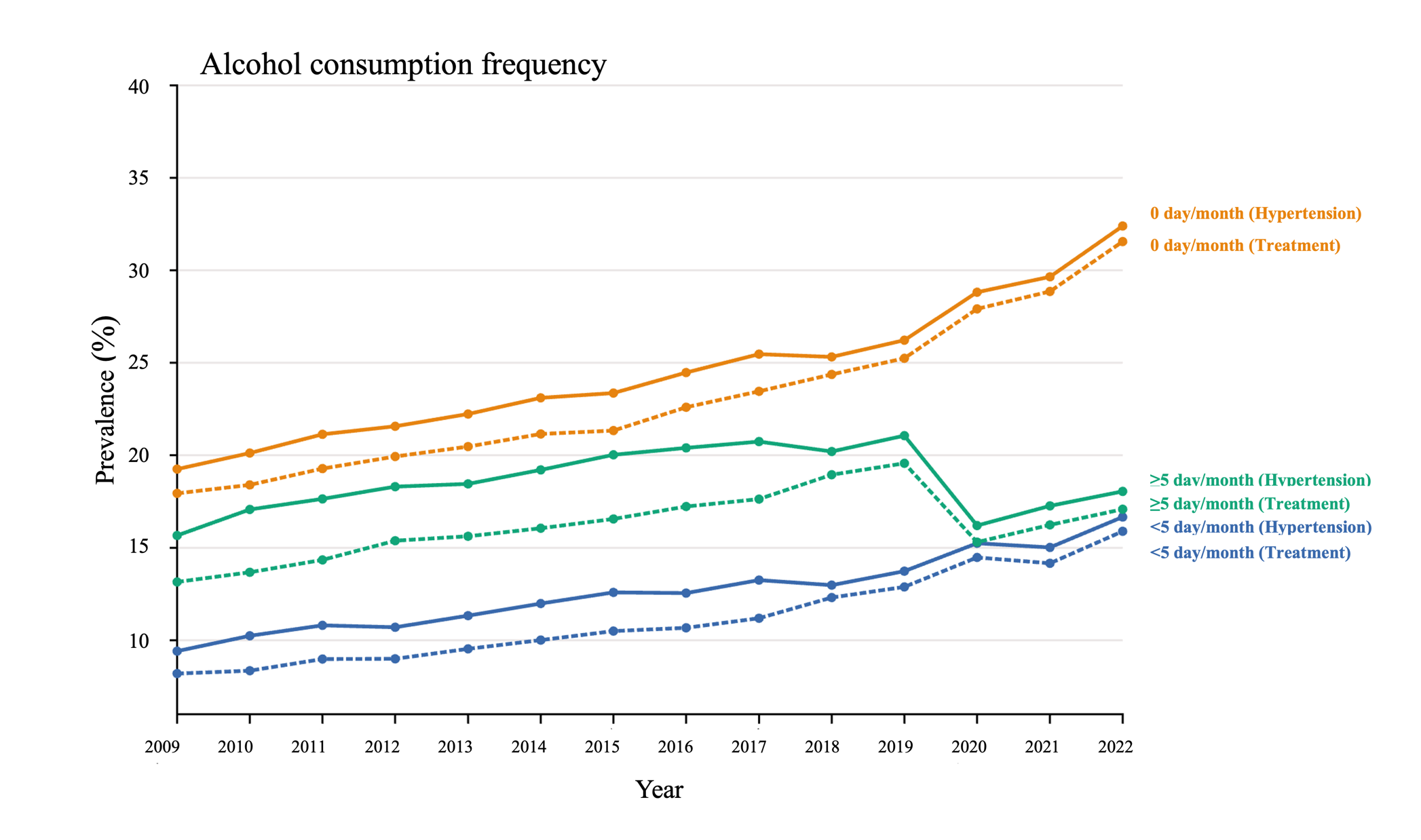


**Figure S10.** Basic livelihood security recipient-stratification trends in prevalence of from Korean adults, 2009-2022.


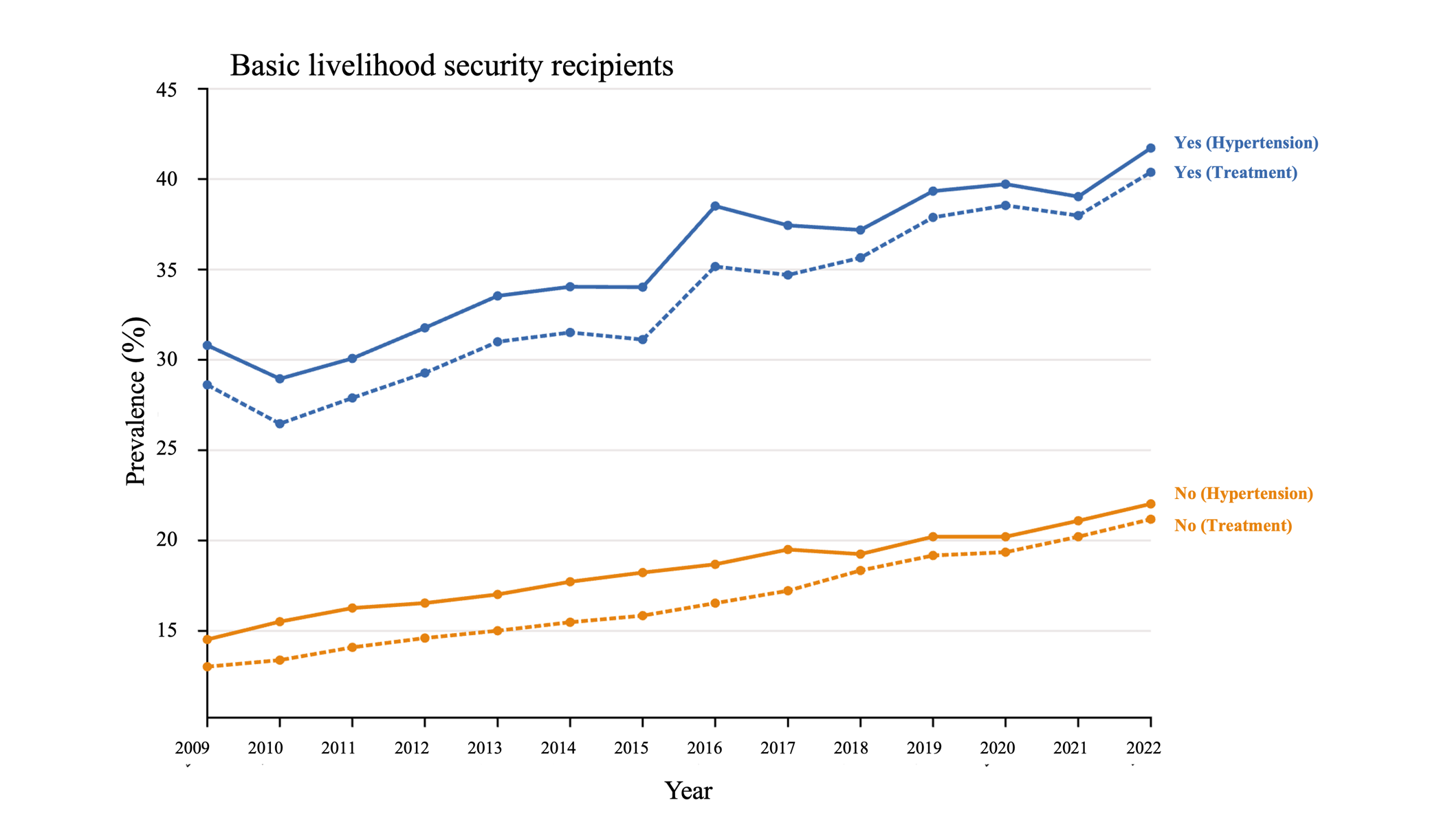


**Figure S11.** Depression status-stratification trends in prevalence of from Korean adults, 2009-2022.


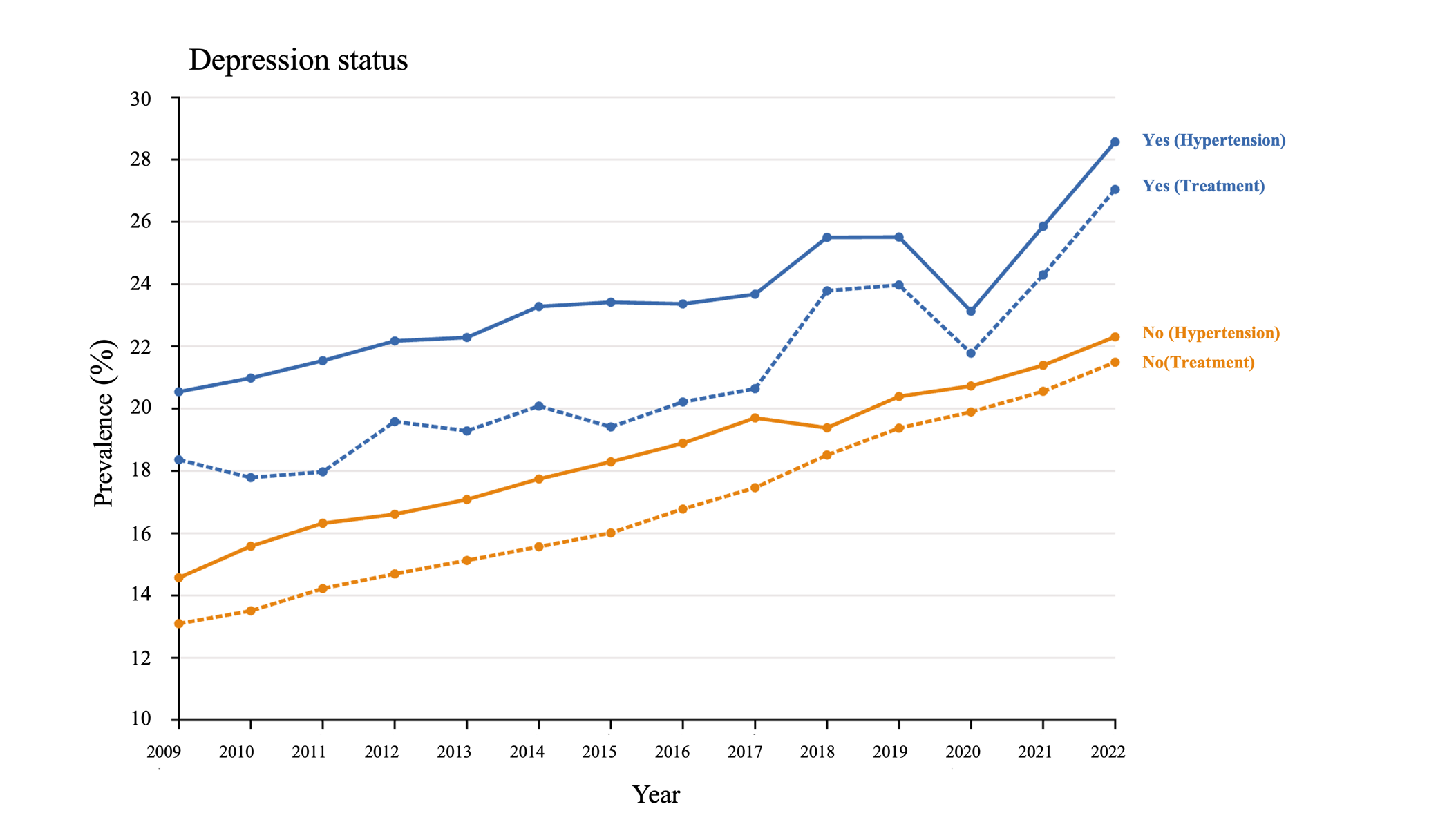


**Figure S12.** Marital status-stratification trends in prevalence of from Korean adults, 2009-2022.


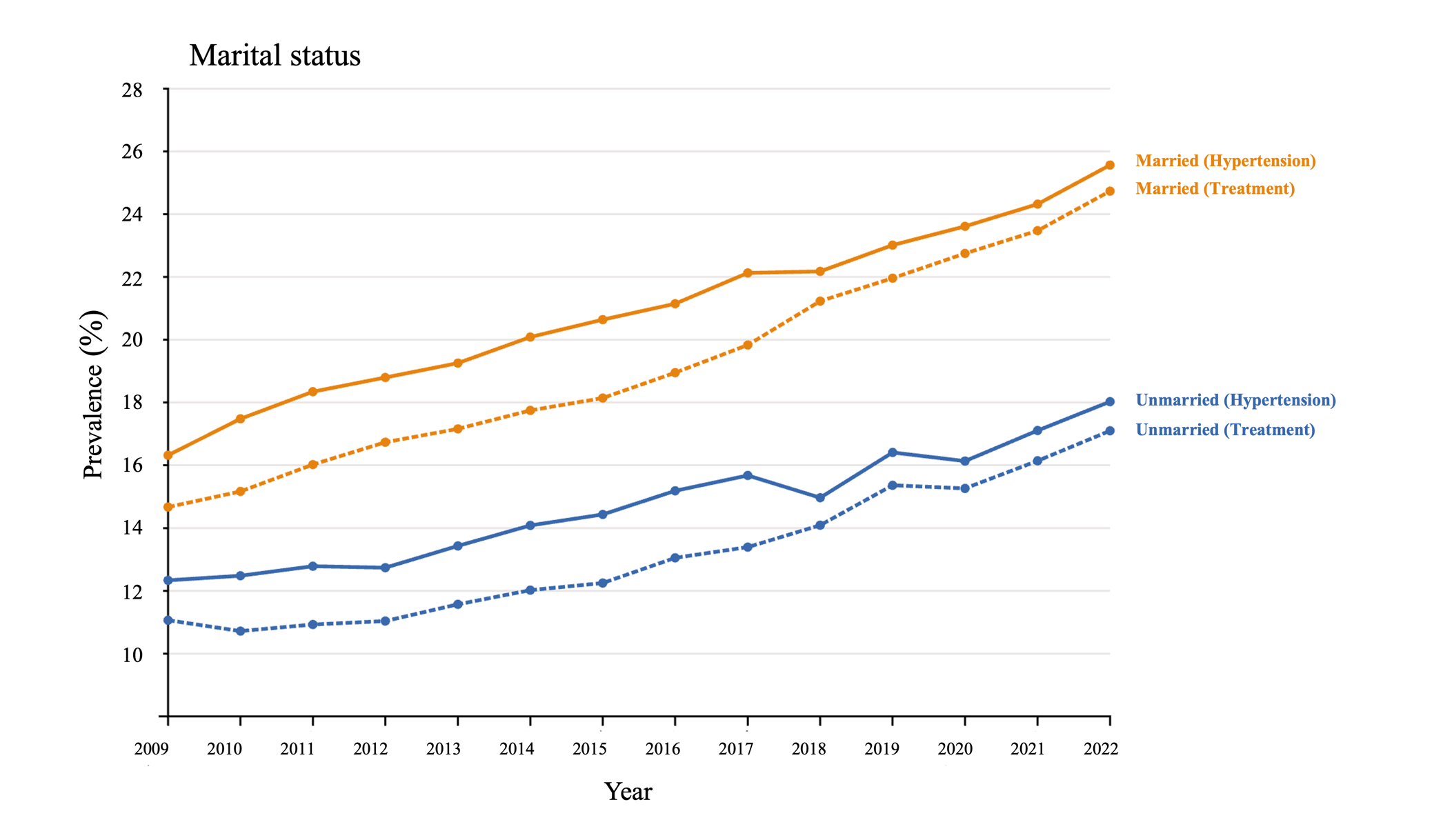


**Figure S13.** Educational background-stratification trends in prevalence of from Korean adults, 2009-2022.


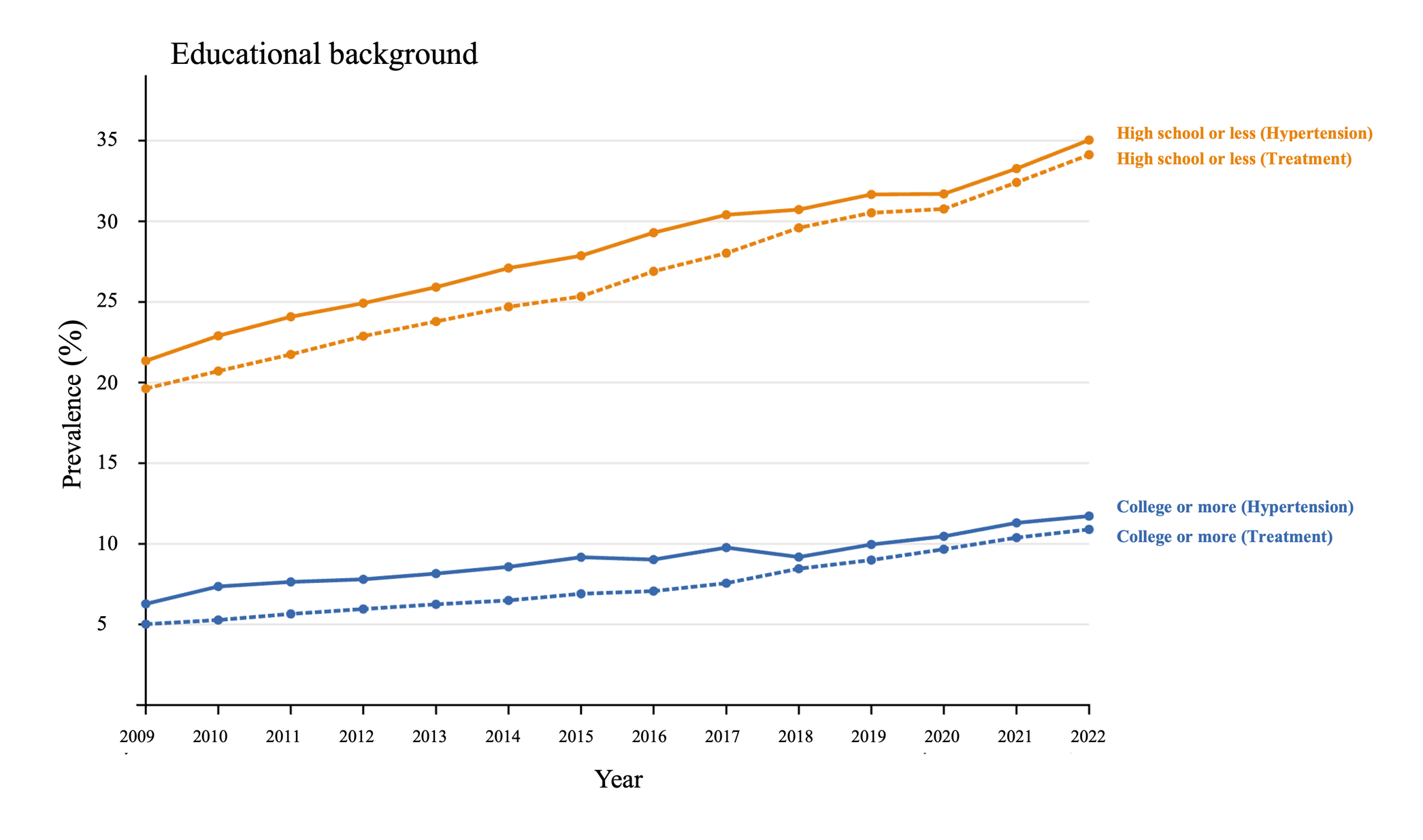


**Figure S14.** Trends in overall hypertension among Korean, 2009-2022 (2-3 years analysis)

**
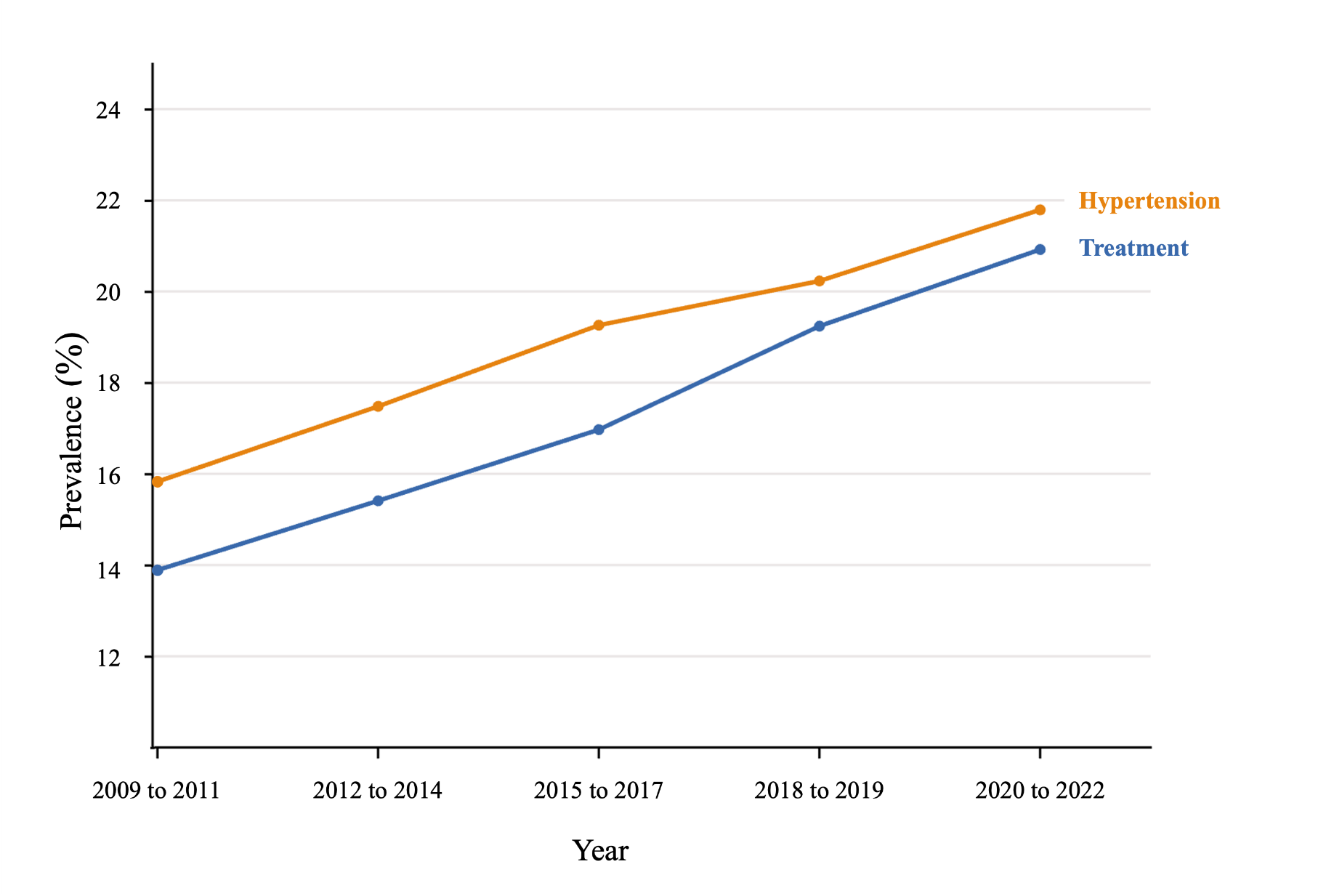
**

**Table S1.** General characteristics of South Korean adults, 2009-2022 (2-3 years analysis)

| Variables, n (%) | Total | 2009 to 2011 | 2012 to 2014 | 2015 to 2017 | 2018 to 2019 | 2020 to 2022 |
| --- | --- | --- | --- | --- | --- | --- |
| **Overall** | 3,072,546 | 660,116 | 648,041 | 655,372 | 430,882 | 678,135 |
| **Age, years-old** |  |  |  |  |  |  |
| 19 to 39 | 794,239 (25.85) | 202,154 (30.62) | 178,962 (27.62) | 166,153 (25.35) | 98,144 (22.78) | 148,826 (21.95) |
| 40 to 59 | 1,179,388 (38.38) | 265,060 (40.15) | 265,486 (40.97) | 255,864 (39.04) | 157,154 (36.47) | 235,824 (34.78) |
| 60 to 79 | 948,097 (30.86) | 173,044 (26.21) | 181,836 (28.06) | 202,211 (30.85) | 148,523 (34.47) | 242,483 (35.76) |
| ≥80 | 150,822 (4.91) | 19,858 (3.01) | 21,757 (3.36) | 31,144 (4.75) | 27,061 (6.28) | 51,002 (7.52) |
| **Sex** |  |  |  |  |  |  |
| Men | 1,426,379 (46.42) | 309,017 (46.81) | 302,842 (46.73) | 303,925 (46.37) | 197,925 (45.93) | 312,670 (46.11) |
| Women | 1,646,167 (53.58) | 351,099 (53.19) | 345,199 (53.27) | 351,447 (53.63) | 232,957 (54.07) | 365,465 (53.89) |
| **Region of residence** |  |  |  |  |  |  |
| Urban | 1,512,260 (49.22) | 322,751 (48.89) | 322,847 (49.82) | 323,472 (49.36) | 212,338 (49.28) | 330,852 (48.79) |
| Rural | 1,560,286 (50.78) | 337,365 (51.11) | 325,194 (50.18) | 331,900 (50.64) | 218,544 (50.72) | 347,283 (51.21) |
| **Basic livelihood security recipient** |  |  |  |  |  |  |
| No | 2,966,487 (96.55) | 635,821 (96.32) | 628,528 (96.99) | 634,960 (96.89) | 417,024 (96.78) | 650,154 (95.87) |
| Yes | 106,059 (3.45) | 24,295 (3.68) | 19,513 (3.01) | 20,412 (3.11) | 13,858 (3.22) | 27,981 (4.13) |
| **Economic level of family** |  |  |  |  |  |  |
| Low | 1,462,162 (47.59) | 364,252 (55.18) | 334,543 (51.62) | 349,969 (53.40) | 163,045 (37.84) | 250,353 (36.92) |
| High | 1,324,566 (43.11) | 253,377 (38.38) | 292,658 (45.16) | 299,340 (45.67) | 190,867 (44.30) | 288,324 (42.52) |
| Unknown | 285,818 (9.30) | 42,487 (6.44) | 20,840 (3.22) | 6,063 (0.93) | 76,970 (17.86) | 139,458 (20.56) |
| **Smoking status** |  |  |  |  |  |  |
| No | 2,475,936 (80.58) | 509,105 (77.12) | 510,986 (78.85) | 533,468 (81.40) | 355,951 (82.61) | 566,426 (83.53) |
| Yes | 596,610 (19.42) | 151,011 (22.88) | 137,055 (21.15) | 121,904 (18.60) | 74,931 (17.39) | 111,709 (16.47) |
| **Alcohol consumption frequency, days/month** |  |  |  |  |  |  |
| 0 | 1,452,795 (47.28) | 317,975 (48.17) | 303,323 (46.81) | 304,801 (46.51) | 210,191 (48.78) | 316,505 (46.67) |
| <5 | 890,138 (28.97) | 203,506 (30.83) | 201,170 (31.04) | 202,648 (30.92) | 126,862 (29.44) | 155,952 (23.00) |
| ≥5 | 729,613 (23.75) | 151,011 (22.88) | 137,055 (21.15) | 121,904 (18.60) | 74,931 (17.39) | 111,709 (16.47) |
| **BMI group** |  |  |  |  |  |  |
| Normal | 1,486,141 (48.37) | 348,864 (52.85) | 331,309 (51.12) | 317,287 (48.41) | 183,104 (42.50) | 305,577 (45.06) |
| Overweight to obese | 1,586,405 (51.63) | 311,252 (47.15) | 316,732 (48.88) | 338,085 (51.59) | 247,778 (57.50) | 372,558 (54.94) |
| **Depression status** |  |  |  |  |  |  |
| No | 2,882,342 (93.81) | 620,302 (93.97) | 609,773 (94.09) | 614,478 (93.76) | 405,619 (94.14) | 632,170 (93.22) |
| Yes | 190,204 (6.19) | 39,814 (6.03) | 38,268 (5.91) | 40,894 (6.24) | 25,263 (5.86) | 45,965 (6.78) |
| **Educational background** |  |  |  |  |  |  |
| High school or lower | 1,958,634 (63.75) | 444,564 (67.35) | 418,027 (64.51) | 411,596 (62.80) | 271,672 (63.05) | 412,775 (60.87) |
| College or higher | 1,113,912 (36.25) | 215,552 (32.65) | 230,014 (35.49) | 243,776 (37.20) | 159,210 (36.95) | 265,360 (39.13) |
| **Occupation status** |  |  |  |  |  |  |
| White-collar | 608,683 (19.81) | 123,981 (18.78) | 130,045 (20.07) | 132,613 (20.23) | 84,146 (19.53) | 137,898 (20.33) |
| Blue-collar | 1,323,333 (43.07) | 276,157 (41.83) | 285,818 (44.10) | 289,784 (44.22) | 186,725 (43.34) | 284,849 (42.00) |
| Unemployed | 1,140,530 (37.12) | 259,978 (39.38) | 232,178 (35.83) | 232,975 (35.55) | 160,011 (37.14) | 255,388 (37.66) |
| **Marital status** |  |  |  |  |  |  |
| Married | 2,145,535 (69.83) | 473,453 (71.72) | 465,972 (71.90) | 459,296 (70.08) | 297,130 (68.96) | 449,684 (66.31) |
| Unmarried | 927,011 (30.17) | 186,663 (28.28) | 182,069 (28.10) | 196,076 (29.92) | 133,752 (31.04) | 228,451 (33.69) |
| **Hypertension** |  |  |  |  |  |  |
| No | 2,310,650 (75.20) | 524,961 (79.53) | 500,623 (77.25) | 489,316 (74.66) | 311,835 (72.37) | 483,915 (71.36) |
| Yes | 761,896 (24.80) | 135,155 (20.47) | 147,418 (22.75) | 166,056 (25.34) | 119,047 (27.63) | 194,220 (28.64) |
| **Hypertension treatment** |  |  |  |  |  |  |
| No | 2,360,282 (76.82) | 537,409 (81.41) | 513,541 (79.25) | 503,889 (76.89) | 316,072 (73.35) | 489,371 (72.16) |
| Yes | 712,264 (23.18) | 122,707 (18.59) | 134,500 (20.75) | 151,483 (23.11) | 114,810 (26.65) | 188,764 (27.84) |

BMI, body mass index.

**Table S2.** Nationwide trends in prevalence of hypertension and receiving treatment for hypertension, 2009-2022 (2-3 years analysis)

|  |  |  | **Before pandemic** | | | | **During pandemic** |
| --- | --- | --- | --- | --- | --- | --- | --- |
| **Weighted % (95% CI)** |  | **Total** | **2009 to 2011** | **2012 to 2014** | **2015 to 2017** | **2018 to 2019** | **2020 to 2022** |
| **Overall** | Hypertension | 19.01 (18.94 to 19.07) | 15.83 (15.71 to 15.96) | 17.48 (17.35 to 17.62) | 19.26 (19.12 to 19.40) | 20.23 (20.05 to 20.41) | 21.79 (21.65 to 21.94) |
|  | Treatment | 17.39 (17.32 to 17.45) | 13.89 (13.77 to 14.01) | 15.41 (15.29 to 15.54) | 16.97 (16.84 to 17.10) | 19.24 (19.07 to 19.42) | 20.92 (20.78 to 21.06) |
| **Age, years-old** |  |  |  |  |  |  |  |
| 19 to 39 | Hypertension | 2.53 (2.48 to 2.57) | 2.29 (2.21 to 2.37) | 2.52 (2.43 to 2.61) | 2.87 (2.77 to 2.97) | 2.19 (2.08 to 2.30) | 2.77 (2.67 to 2.87) |
|  | Treatment | 1.20 (1.17 to 1.23) | 0.84 (0.79 to 0.89) | 0.89 (0.84 to 0.94) | 0.97 (0.92 to 1.03) | 1.43 (1.34 to 1.52) | 1.93 (1.85 to 2.02) |
| 40 to 59 | Hypertension | 16.87 (16.77 to 16.96) | 15.81 (15.62 to 15.99) | 16.48 (16.29 to 16.67) | 16.99 (16.80 to 17.18) | 16.99 (16.74 to 17.25) | 17.97 (17.77 to 18.18) |
|  | Treatment | 15.08 (14.99 to 15.17) | 13.53 (13.36 to 13.71) | 14.17 (13.99 to 14.34) | 14.53 (14.35 to 14.71) | 16.01 (15.76 to 16.25) | 17.04 (16.84 to 17.24) |
| 60 to 79 | Hypertension | 46.87 (46.72 to 47.02) | 45.59 (45.24 to 45.93) | 47.49 (47.15 to 47.82) | 47.80 (47.48 to 48.12) | 47.54 (47.17 to 47.91) | 45.94 (45.66 to 46.23) |
|  | Treatment | 45.10 (44.96 to 45.25) | 43.26 (42.91 to 43.60) | 45.07 (44.74 to 45.41) | 45.23 (44.91 to 45.55) | 46.29 (45.92 to 46.66) | 45.12 (44.83 to 45.41) |
| ≥80 | Hypertension | 59.44 (59.06 to 59.81) | 51.40 (50.37 to 52.42) | 57.40 (56.43 to 58.38) | 60.25 (59.43 to 61.06) | 62.45 (61.60 to 63.31) | 60.37 (59.74 to 61.00) |
|  | Treatment | 57.64 (57.27 to 58.02) | 48.95 (47.92 to 49.97) | 54.88 (53.90 to 55.87) | 57.52 (56.69 to 58.34) | 60.90 (60.03 to 61.77) | 59.46 (58.83 to 60.09) |
| **Sex** |  |  |  |  |  |  |  |
| Men | Hypertension | 19.75 (19.67 to 19.84) | 15.77 (15.61 to 15.94) | 18.07 (17.89 to 18.25) | 20.36 (20.18 to 20.55) | 21.09 (20.86 to 21.33) | 22.98 (22.79 to 23.18) |
|  | Treatment | 17.49 (17.41 to 17.57) | 13.09 (12.94 to 13.24) | 15.15 (14.99 to 15.32) | 17.15 (16.98 to 17.32) | 19.74 (19.51 to 19.97) | 21.73 (21.54 to 21.92) |
| Women | Hypertension | 18.25 (18.17 to 18.34) | 15.89 (15.73 to 16.06) | 16.89 (16.72 to 17.06) | 18.15 (17.97 to 18.32) | 19.36 (19.14 to 19.58) | 20.62 (20.43 to 20.80) |
|  | Treatment | 17.29 (17.21 to 17.37) | 14.68 (14.53 to 14.84) | 15.67 (15.51 to 15.83) | 16.80 (16.63 to 16.96) | 18.74 (18.52 to 18.96) | 20.12 (19.94 to 20.30) |
| **Region of residence** |  |  |  |  |  |  |  |
| Urban | Hypertension | 18.23 (18.15 to 18.32) | 15.26 (15.10 to 15.42) | 16.90 (16.74 to 17.06) | 18.52 (18.35 to 18.69) | 19.40 (19.18 to 19.62) | 20.70 (20.52 to 20.87) |
|  | Treatment | 16.59 (16.51 to 16.66) | 13.28 (13.13 to 13.42) | 14.77 (14.62 to 14.92) | 16.18 (16.02 to 16.34) | 18.42 (18.21 to 18.63) | 19.80 (19.62 to 19.97) |
| Rural | Hypertension | 20.81 (20.70 to 20.93) | 17.16 (16.95 to 17.37) | 18.86 (18.64 to 19.09) | 21.00 (20.76 to 21.23) | 22.18 (21.87 to 22.50) | 24.37 (24.11 to 24.64) |
|  | Treatment | 19.27 (19.15 to 19.38) | 15.30 (15.10 to 15.50) | 16.92 (16.70 to 17.13) | 18.83 (18.60 to 19.05) | 21.18 (20.87 to 21.49) | 23.56 (23.30 to 23.81) |
| **Basic livelihood security recipient** |  |  |  |  |  |  |  |
| No | Hypertension | 18.52 (18.45 to 18.58) | 15.44 (15.31 to 15.56) | 17.10 (16.97 to 17.23) | 18.82 (18.68 to 18.95) | 19.74 (19.56 to 19.92) | 21.12 (20.97 to 21.27) |
|  | Treatment | 16.91 (16.85 to 16.98) | 13.50 (13.38 to 13.62) | 15.04 (14.92 to 15.16) | 16.55 (16.42 to 16.68) | 18.77 (18.59 to 18.94) | 20.26 (20.12 to 20.40) |
| Yes | Hypertension | 36.10 (35.67 to 36.53) | 29.97 (29.12 to 30.83) | 33.14 (32.20 to 34.08) | 36.65 (35.68 to 37.62) | 38.24 (37.08 to 39.40) | 40.22 (39.40 to 41.03) |
|  | Treatment | 34.10 (33.68 to 34.52) | 27.69 (26.86 to 28.52) | 30.62 (29.71 to 31.53) | 33.66 (32.72 to 34.60) | 36.74 (35.60 to 37.89) | 39.02 (38.21 to 39.83) |
| **Economic level of family** |  |  |  |  |  |  |  |
| Low | Hypertension | 27.09 (26.98 to 27.20) | 20.30 (20.10 to 20.49) | 24.22 (24.01 to 24.44) | 27.08 (26.85 to 27.30) | 33.44 (33.08 to 33.79) | 34.54 (34.25 to 34.82) |
|  | Treatment | 25.22 (25.11 to 25.33) | 18.26 (18.08 to 18.45) | 22.00 (21.80 to 22.21) | 24.60 (24.38 to 24.81) | 32.21 (31.86 to 32.56) | 33.59 (33.31 to 33.87) |
| High | Hypertension | 13.80 (13.73 to 13.88) | 11.43 (11.27 to 11.58) | 12.36 (12.21 to 12.52) | 13.64 (13.48 to 13.79) | 14.64 (14.43 to 14.84) | 16.37 (16.20 to 16.54) |
|  | Treatment | 12.30 (12.22 to 12.37) | 9.56 (9.42 to 9.71) | 10.41 (10.27 to 10.55) | 11.49 (11.35 to 11.64) | 13.78 (13.58 to 13.98) | 15.58 (15.41 to 15.75) |
| Unknown | Hypertension | 17.13 (16.93 to 17.33) | 16.11 (15.65 to 16.58) | 18.40 (17.69 to 19.10) | 17.35 (16.10 to 18.61) | 16.60 (16.22 to 16.99) | 17.69 (17.40 to 17.98) |
|  | Treatment | 15.87 (15.68 to 16.06) | 14.21 (13.77 to 14.65) | 16.19 (15.53 to 16.84) | 15.00 (13.82 to 16.18) | 15.60 (15.23 to 15.97) | 16.69 (16.41 to 16.98) |
| **Smoking status** |  |  |  |  |  |  |  |
| No | Hypertension | 19.99 (19.92 to 20.07) | 17.09 (16.94 to 17.24) | 18.57 (18.42 to 18.72) | 20.12 (19.96 to 20.28) | 21.13 (20.93 to 21.32) | 22.39 (22.22 to 22.55) |
|  | Treatment | 18.58 (18.51 to 18.66) | 15.38 (15.24 to 15.52) | 16.74 (16.59 to 16.88) | 18.12 (17.97 to 18.27) | 20.25 (20.05 to 20.44) | 21.62 (21.46 to 21.78) |
| Yes | Hypertension | 15.37 (15.25 to 15.49) | 12.07 (11.86 to 12.28) | 13.96 (13.72 to 14.19) | 16.02 (15.76 to 16.28) | 16.60 (16.26 to 16.94) | 19.11 (18.81 to 19.41) |
|  | Treatment | 12.99 (12.87 to 13.10) | 9.41 (9.22 to 9.59) | 11.09 (10.88 to 11.29) | 12.64 (12.40 to 12.87) | 15.17 (14.84 to 15.49) | 17.74 (17.45 to 18.03) |
| **Alcohol consumption frequency, days/month** |  |  |  |  |  |  |  |
| 0 | Hypertension | 24.63 (24.52 to 24.73) | 20.15 (19.95 to 20.34) | 22.30 (22.09 to 22.51) | 24.45 (24.23 to 24.67) | 25.78 (25.51 to 26.06) | 30.23 (29.99 to 30.47) |
|  | Treatment | 23.20 (23.10 to 23.30) | 18.52 (18.33 to 18.72) | 20.52 (20.31 to 20.72) | 22.48 (22.27 to 22.70) | 24.82 (24.56 to 25.09) | 29.39 (29.15 to 29.62) |
| <5 | Hypertension | 12.56 (12.47 to 12.65) | 10.16 (9.99 to 10.32) | 11.35 (11.18 to 11.52) | 12.80 (12.62 to 12.98) | 13.36 (13.12 to 13.59) | 15.68 (15.46 to 15.91) |
|  | Treatment | 11.12 (11.04 to 11.21) | 8.52 (8.37 to 8.66) | 9.52 (9.37 to 9.68) | 10.79 (10.62 to 10.96) | 12.60 (12.37 to 12.83) | 14.88 (14.66 to 15.10) |
| ≥5 | Hypertension | 18.62 (18.50 to 18.74) | 16.82 (16.56 to 17.08) | 18.67 (18.41 to 18.93) | 20.39 (20.12 to 20.66) | 20.62 (20.28 to 20.97) | 17.19 (16.98 to 17.39) |
|  | Treatment | 16.47 (16.35 to 16.58) | 13.74 (13.51 to 13.98) | 15.70 (15.46 to 15.94) | 17.15 (16.90 to 17.40) | 19.25 (18.92 to 19.58) | 16.22 (16.02 to 16.43) |
| **BMI group** |  |  |  |  |  |  |  |
| Normal | Hypertension | 12.07 (12.00 to 12.14) | 10.15 (10.01 to 10.28) | 11.32 (11.18 to 11.46) | 12.44 (12.29 to 12.59) | 12.11 (11.91 to 12.30) | 14.45 (14.28 to 14.61) |
|  | Treatment | 10.96 (10.89 to 11.03) | 8.87 (8.74 to 8.99) | 9.94 (9.81 to 10.07) | 10.93 (10.78 to 11.07) | 11.41 (11.22 to 11.60) | 13.84 (13.68 to 14.01) |
| Overweight to obese | Hypertension | 25.57 (25.47 to 25.66) | 22.42 (22.22 to 22.62) | 24.14 (23.93 to 24.34) | 25.83 (25.63 to 26.04) | 26.57 (26.32 to 26.82) | 27.85 (27.65 to 28.05) |
|  | Treatment | 23.47 (23.37 to 23.56) | 19.70 (19.51 to 19.89) | 21.31 (21.12 to 21.51) | 22.80 (22.60 to 22.99) | 25.36 (25.11 to 25.60) | 26.75 (26.55 to 26.95) |
| **Depression status** |  |  |  |  |  |  |  |
| No | Hypertension | 18.68 (18.61 to 18.75) | 15.49 (15.37 to 15.62) | 17.15 (17.01 to 17.28) | 18.97 (18.83 to 19.11) | 19.89 (19.71 to 20.07) | 21.48 (21.33 to 21.63) |
|  | Treatment | 17.12 (17.05 to 17.18) | 13.61 (13.49 to 13.73) | 15.13 (15.01 to 15.26) | 16.76 (16.63 to 16.89) | 18.95 (18.77 to 19.12) | 20.65 (20.51 to 20.80) |
| Yes | Hypertension | 23.85 (23.59 to 24.10) | 20.97 (20.45 to 21.48) | 22.64 (22.09 to 23.19) | 23.49 (22.95 to 24.03) | 25.51 (24.80 to 26.21) | 26.07 (25.54 to 26.59) |
|  | Treatment | 21.41 (21.17 to 21.65) | 18.08 (17.60 to 18.56) | 19.68 (19.17 to 20.20) | 20.09 (19.59 to 20.59) | 23.88 (23.20 to 24.57) | 24.58 (24.06 to 25.09) |
| **Educational background** |  |  |  |  |  |  |  |
| High school or less | Hypertension | 28.45 (28.35 to 28.54) | 22.74 (22.56 to 22.92) | 25.98 (25.79 to 26.18) | 29.19 (28.99 to 29.40) | 31.20 (30.94 to 31.46) | 33.33 (33.12 to 33.55) |
|  | Treatment | 26.70 (26.60 to 26.79) | 20.67 (20.50 to 20.84) | 23.79 (23.60 to 23.98) | 26.76 (26.56 to 26.96) | 30.07 (29.81 to 30.32) | 32.43 (32.22 to 32.64) |
| College or more | Hypertension | 9.20 (9.13 to 9.27) | 7.11 (6.97 to 7.25) | 8.19 (8.04 to 8.33) | 9.33 (9.18 to 9.47) | 9.57 (9.39 to 9.75) | 11.17 (11.02 to 11.33) |
|  | Treatment | 7.72 (7.66 to 7.79) | 5.32 (5.21 to 5.44) | 6.24 (6.12 to 6.36) | 7.18 (7.06 to 7.31) | 8.73 (8.55 to 8.90) | 10.33 (10.18 to 10.47) |
| **Occupation status** |  |  |  |  |  |  |  |
| White-collar | Hypertension | 10.00 (9.90 to 10.09) | 8.50 (8.31 to 8.70) | 9.11 (8.92 to 9.31) | 10.16 (9.96 to 10.36) | 10.28 (10.02 to 10.53) | 11.54 (11.34 to 11.75) |
|  | Treatment | 8.41 (8.32 to 8.50) | 6.48 (6.31 to 6.65) | 6.99 (6.82 to 7.16) | 7.92 (7.74 to 8.10) | 9.44 (9.20 to 9.69) | 10.68 (10.48 to 10.88) |
| Blue-collar | Hypertension | 19.03 (18.94 to 19.13) | 14.89 (14.71 to 15.07) | 17.30 (17.11 to 17.49) | 19.43 (19.23 to 19.64) | 20.59 (20.33 to 20.86) | 22.48 (22.26 to 22.69) |
|  | Treatment | 17.19 (17.10 to 17.29) | 12.69 (12.52 to 12.86) | 14.98 (14.81 to 15.16) | 16.82 (16.63 to 17.01) | 19.48 (19.23 to 19.74) | 21.48 (21.27 to 21.70) |
| Unemployed | Hypertension | 25.55 (25.43 to 25.66) | 21.35 (21.13 to 21.57) | 23.89 (23.65 to 24.12) | 26.07 (25.82 to 26.32) | 27.31 (27.00 to 27.62) | 28.86 (28.61 to 29.11) |
|  | Treatment | 24.13 (24.02 to 24.25) | 19.70 (19.49 to 19.91) | 22.10 (21.87 to 22.34) | 24.10 (23.86 to 24.34) | 26.34 (26.03 to 26.64) | 28.09 (27.84 to 28.34) |
| **Marital status** |  |  |  |  |  |  |  |
| Married | Hypertension | 21.08 (20.99 to 21.16) | 17.39 (17.22 to 17.55) | 19.38 (19.22 to 19.55) | 21.31 (21.13 to 21.49) | 22.60 (22.37 to 22.82) | 24.50 (24.31 to 24.70) |
|  | Treatment | 19.39 (19.31 to 19.47) | 15.29 (15.14 to 15.44) | 17.22 (17.06 to 17.38) | 18.98 (18.81 to 19.15) | 21.60 (21.37 to 21.82) | 23.66 (23.47 to 23.85) |
| Unmarried | Hypertension | 14.93 (14.83 to 15.02) | 12.53 (12.35 to 12.72) | 13.44 (13.24 to 13.63) | 15.11 (14.91 to 15.32) | 15.71 (15.46 to 15.96) | 17.10 (16.90 to 17.30) |
|  | Treatment | 13.44 (13.35 to 13.53) | 10.91 (10.74 to 11.07) | 11.56 (11.38 to 11.73) | 12.91 (12.73 to 13.10) | 14.74 (14.50 to 14.98) | 16.18 (15.98 to 16.38) |

BMI, body mass index; CI, confidence interval.

**Table S3.** Nationwide trends in prevalence of hypertension and receiving treatment for hypertension, 2009-2022 (unweighted)

|  |  |  | **Before COVID-19 pandemic** | | | | | | | | | | | **During COVID-19 pandemic** | | |
| --- | --- | --- | --- | --- | --- | --- | --- | --- | --- | --- | --- | --- | --- | --- | --- | --- |
| **Weighted % (95% CI)** |  | **Total** | **2009** | **2010** | **2011** | **2012** | **2013** | **2014** | **2015** | **2016** | **2017** | **2018** | **2019** | **2020** | **2021** | **2022** |
| **Overall** | Hypertension | 24.80 (24.75 to 24.85) | 19.29 (19.13 to 19.46) | 20.52 (20.35 to 20.68) | 21.65 (21.48 to 21.82) | 21.88 (21.71 to 22.06) | 22.97 (22.79 to 23.15) | 23.39 (23.21 to 23.57) | 24.48 (24.30 to 24.66) | 25.05 (24.87 to 25.23) | 26.47 (26.28 to 26.65) | 26.71 (26.52 to 26.90) | 28.52 (28.33 to 28.71) | 27.36 (27.17 to 27.54) | 28.45 (28.26 to 28.63) | 30.09 (29.90 to 30.28) |
|  | Treatment | 23.18 (23.13 to 23.23) | 17.78 (17.62 to 17.94) | 18.45 (18.29 to 18.61) | 19.56 (19.40 to 19.73) | 20.06 (19.89 to 20.22) | 21.00 (20.83 to 21.17) | 21.20 (21.03 to 21.38) | 22.21 (22.04 to 22.39) | 22.89 (22.72 to 23.07) | 24.23 (24.05 to 24.41) | 25.74 (25.55 to 25.93) | 27.52 (27.34 to 27.71) | 26.57 (26.39 to 26.75) | 27.64 (27.45 to 27.82) | 29.27 (29.09 to 29.46) |
| **Age, years-old** |  |  |  |  |  |  |  |  |  |  |  |  |  |  |  |  |
| 19 to 39 | Hypertension | 2.50 (2.47 to 2.54) | 1.94 (1.84 to 2.05) | 2.42 (2.31 to 2.54) | 2.42 (2.30 to 2.54) | 2.26 (2.15 to 2.38) | 2.36 (2.24 to 2.48) | 2.69 (2.56 to 2.82) | 2.89 (2.75 to 3.03) | 2.68 (2.55 to 2.81) | 2.92 (2.78 to 3.06) | 1.96 (1.84 to 2.08) | 2.52 (2.38 to 2.67) | 2.44 (2.31 to 2.58) | 2.86 (2.72 to 3.01) | 2.91 (2.75 to 3.06) |
|  | Treatment | 1.22 (1.20 to 1.25) | 0.95 (0.88 to 1.02) | 0.94 (0.87 to 1.02) | 0.86 (0.79 to 0.93) | 0.92 (0.85 to 1.00) | 0.95 (0.87 to 1.03) | 1.00 (0.92 to 1.08) | 1.01 (0.93 to 1.09) | 0.99 (0.91 to 1.07) | 1.15 (1.06 to 1.24) | 1.37 (1.27 to 1.47) | 1.70 (1.58 to 1.81) | 1.73 (1.62 to 1.84) | 2.01 (1.89 to 2.13) | 2.14 (2.01 to 2.27) |
| 40 to 59 | Hypertension | 17.50 (17.43 to 17.57) | 15.88 (15.64 to 16.12) | 16.39 (16.14 to 16.63) | 16.96 (16.72 to 17.21) | 16.87 (16.62 to 17.12) | 17.16 (16.91 to 17.41) | 17.30 (17.05 to 17.55) | 17.48 (17.23 to 17.73) | 17.66 (17.40 to 17.92) | 18.23 (17.97 to 18.50) | 17.83 (17.56 to 18.09) | 17.82 (17.55 to 18.09) | 18.19 (17.92 to 18.45) | 18.58 (18.30 to 18.85) | 19.13 (18.85 to 19.40) |
|  | Treatment | 15.73 (15.66 to 15.80) | 14.13 (13.90 to 14.36) | 14.03 (13.80 to 14.26) | 14.68 (14.44 to 14.91) | 14.87 (14.64 to 15.11) | 15.08 (14.84 to 15.31) | 14.98 (14.75 to 15.22) | 15.02 (14.78 to 15.26) | 15.44 (15.19 to 15.68) | 15.88 (15.64 to 16.13) | 16.91 (16.65 to 17.17) | 16.83 (16.57 to 17.10) | 17.32 (17.06 to 17.58) | 17.72 (17.45 to 17.99) | 18.27 (17.99 to 18.54) |
| 60 to 79 | Hypertension | 47.15 (47.05 to 47.25) | 43.11 (42.70 to 43.52) | 44.57 (44.17 to 44.98) | 45.96 (45.56 to 46.36) | 46.22 (45.82 to 46.62) | 47.04 (46.65 to 47.44) | 47.70 (47.30 to 48.09) | 47.61 (47.23 to 47.99) | 48.31 (47.93 to 48.69) | 48.51 (48.14 to 48.88) | 48.83 (48.46 to 49.19) | 48.26 (47.91 to 48.61) | 46.84 (46.49 to 47.19) | 47.29 (46.94 to 47.64) | 48.14 (47.81 to 48.48) |
|  | Treatment | 45.46 (45.36 to 45.56) | 41.36 (40.95 to 41.76) | 42.31 (41.91 to 42.72) | 43.66 (43.26 to 44.06) | 44.17 (43.77 to 44.57) | 44.81 (44.41 to 45.20) | 45.27 (44.88 to 45.67) | 45.28 (44.89 to 45.66) | 45.92 (45.54 to 46.30) | 46.12 (45.75 to 46.48) | 47.60 (47.23 to 47.97) | 47.17 (46.82 to 47.52) | 46.09 (45.74 to 46.44) | 46.59 (46.24 to 46.93) | 47.33 (46.99 to 47.67) |
| ≥80 | Hypertension | 58.75 (58.50 to 59.00) | 47.37 (46.18 to 48.56) | 50.96 (49.75 to 52.17) | 52.69 (51.48 to 53.90) | 53.71 (52.50 to 54.92) | 55.86 (54.72 to 57.01) | 57.88 (56.80 to 58.97) | 58.16 (57.14 to 59.18) | 59.72 (58.77 to 60.67) | 59.87 (58.99 to 60.75) | 63.71 (62.82 to 64.60) | 60.77 (60.01 to 61.53) | 59.82 (59.04 to 60.59) | 61.21 (60.47 to 61.95) | 62.21 (61.52 to 62.90) |
|  | Treatment | 57.04 (56.79 to 57.29) | 45.45 (44.27 to 46.64) | 48.55 (47.34 to 49.76) | 50.11 (48.90 to 51.31) | 51.67 (50.46 to 52.88) | 53.10 (51.95 to 54.26) | 55.33 (54.24 to 56.42) | 55.77 (54.75 to 56.80) | 57.05 (56.10 to 58.01) | 57.13 (56.23 to 58.02) | 62.23 (61.33 to 63.13) | 59.68 (58.92 to 60.45) | 58.97 (58.20 to 59.75) | 60.27 (59.53 to 61.01) | 61.41 (60.71 to 62.10) |
| **Sex** |  |  |  |  |  |  |  |  |  |  |  |  |  |  |  |  |
| Men | Hypertension | 24.79 (24.72 to 24.86) | 18.00 (17.77 to 18.23) | 20.01 (19.77 to 20.26) | 21.47 (21.21 to 21.72) | 21.87 (21.61 to 22.12) | 23.17 (22.91 to 23.43) | 23.67 (23.41 to 23.93) | 24.84 (24.57 to 25.10) | 25.39 (25.12 to 25.66) | 27.04 (26.76 to 27.31) | 27.17 (26.89 to 27.45) | 28.08 (27.80 to 28.36) | 27.36 (27.08 to 27.63) | 28.67 (28.40 to 28.95) | 30.39 (30.11 to 30.67) |
|  | Treatment | 22.56 (22.49 to 22.63) | 15.97 (15.75 to 16.19) | 17.20 (16.97 to 17.43) | 18.58 (18.34 to 18.82) | 19.34 (19.09 to 19.58) | 20.54 (20.29 to 20.79) | 20.62 (20.37 to 20.87) | 21.68 (21.43 to 21.93) | 22.44 (22.18 to 22.70) | 23.92 (23.66 to 24.19) | 25.89 (25.62 to 26.17) | 26.70 (26.42 to 26.97) | 26.24 (25.97 to 26.51) | 27.48 (27.21 to 27.75) | 29.25 (28.98 to 29.53) |
| Women | Hypertension | 24.80 (24.74 to 24.87) | 20.45 (20.22 to 20.68) | 20.96 (20.72 to 21.19) | 21.81 (21.57 to 22.05) | 21.89 (21.65 to 22.13) | 22.79 (22.55 to 23.03) | 23.14 (22.89 to 23.38) | 24.18 (23.93 to 24.42) | 24.75 (24.51 to 25.00) | 25.98 (25.73 to 26.23) | 26.31 (26.05 to 26.57) | 28.88 (28.62 to 29.14) | 27.36 (27.11 to 27.61) | 28.25 (28.00 to 28.51) | 29.83 (29.58 to 30.09) |
|  | Treatment | 23.72 (23.65 to 23.78) | 19.39 (19.17 to 19.62) | 19.56 (19.33 to 19.78) | 20.41 (20.18 to 20.64) | 20.68 (20.45 to 20.92) | 21.41 (21.17 to 21.65) | 21.72 (21.48 to 21.95) | 22.68 (22.44 to 22.92) | 23.28 (23.04 to 23.53) | 24.48 (24.24 to 24.73) | 25.61 (25.35 to 25.86) | 28.21 (27.95 to 28.46) | 26.84 (26.60 to 27.09) | 27.78 (27.53 to 28.03) | 29.29 (29.03 to 29.54) |
| **Region of residence** |  |  |  |  |  |  |  |  |  |  |  |  |  |  |  |  |
| Urban | Hypertension | 21.24 (21.18 to 21.31) | 16.85 (16.63 to 17.07) | 18.17 (17.94 to 18.40) | 18.73 (18.50 to 18.97) | 18.97 (18.73 to 19.20) | 19.74 (19.50 to 19.98) | 20.21 (19.97 to 20.45) | 20.98 (20.74 to 21.23) | 21.26 (21.02 to 21.50) | 22.38 (22.13 to 22.63) | 23.20 (22.95 to 23.45) | 23.97 (23.71 to 24.22) | 23.00 (22.75 to 23.25) | 23.93 (23.68 to 24.19) | 25.81 (25.55 to 26.07) |
|  | Treatment | 19.58 (19.52 to 19.64) | 15.32 (15.11 to 15.54) | 16.01 (15.79 to 16.23) | 16.58 (16.35 to 16.80) | 17.01 (16.79 to 17.24) | 17.76 (17.53 to 17.99) | 18.00 (17.77 to 18.23) | 18.65 (18.41 to 18.88) | 19.07 (18.84 to 19.31) | 20.12 (19.88 to 20.36) | 22.25 (22.00 to 22.50) | 22.94 (22.68 to 23.19) | 22.17 (21.92 to 22.41) | 23.11 (22.86 to 23.36) | 24.90 (24.64 to 25.15) |
| Rural | Hypertension | 28.24 (28.17 to 28.31) | 21.63 (21.39 to 21.87) | 22.75 (22.50 to 22.99) | 24.46 (24.21 to 24.72) | 24.77 (24.52 to 25.03) | 26.21 (25.94 to 26.47) | 26.52 (26.26 to 26.78) | 27.92 (27.66 to 28.19) | 28.74 (28.48 to 29.01) | 30.42 (30.15 to 30.69) | 30.25 (29.98 to 30.53) | 32.78 (32.51 to 33.06) | 31.52 (31.25 to 31.78) | 32.73 (32.46 to 33.00) | 34.17 (33.90 to 34.44) |
|  | Treatment | 26.67 (26.60 to 26.74) | 20.12 (19.89 to 20.36) | 20.77 (20.53 to 21.01) | 22.44 (22.19 to 22.68) | 23.07 (22.82 to 23.32) | 24.25 (24.00 to 24.51) | 24.36 (24.10 to 24.61) | 25.72 (25.46 to 25.98) | 26.61 (26.35 to 26.87) | 28.20 (27.93 to 28.46) | 29.26 (28.99 to 29.54) | 31.82 (31.55 to 32.09) | 30.77 (30.50 to 31.04) | 31.94 (31.67 to 32.21) | 33.44 (33.17 to 33.71) |
| **Basic livelihood security recipient** |  |  |  |  |  |  |  |  |  |  |  |  |  |  |  |  |
| No | Hypertension | 24.23 (24.18 to 24.28) | 18.61 (18.45 to 18.78) | 20.02 (19.85 to 20.19) | 21.17 (20.99 to 21.34) | 21.42 (21.24 to 21.59) | 22.51 (22.33 to 22.69) | 22.89 (22.71 to 23.07) | 23.97 (23.79 to 24.15) | 24.51 (24.32 to 24.69) | 25.94 (25.75 to 26.12) | 26.15 (25.96 to 26.34) | 27.98 (27.79 to 28.17) | 26.69 (26.51 to 26.88) | 27.78 (27.59 to 27.97) | 29.35 (29.16 to 29.54) |
|  | Treatment | 22.63 (22.58 to 22.68) | 17.11 (16.95 to 17.27) | 17.96 (17.79 to 18.12) | 19.09 (18.92 to 19.25) | 19.60 (19.43 to 19.77) | 20.56 (20.38 to 20.73) | 20.71 (20.54 to 20.88) | 21.72 (21.54 to 21.89) | 22.38 (22.20 to 22.55) | 23.71 (23.53 to 23.89) | 25.20 (25.02 to 25.39) | 26.99 (26.81 to 27.18) | 25.92 (25.73 to 26.10) | 26.98 (26.80 to 27.17) | 28.55 (28.36 to 28.74) |
| Yes | Hypertension | 40.63 (40.33 to 40.92) | 34.95 (33.98 to 35.91) | 33.76 (32.72 to 34.79) | 36.26 (35.13 to 37.39) | 36.69 (35.52 to 37.85) | 38.66 (37.45 to 39.88) | 38.84 (37.68 to 40.00) | 40.25 (39.09 to 41.41) | 42.35 (41.16 to 43.53) | 42.95 (41.78 to 44.12) | 43.37 (42.20 to 44.55) | 44.88 (43.71 to 46.05) | 43.77 (42.73 to 44.82) | 44.07 (43.05 to 45.08) | 46.19 (45.22 to 47.17) |
|  | Treatment | 38.68 (38.38 to 38.97) | 33.09 (32.14 to 34.05) | 31.57 (30.55 to 32.59) | 33.91 (32.80 to 35.02) | 34.44 (33.29 to 35.59) | 36.06 (34.86 to 37.26) | 36.51 (35.36 to 37.65) | 37.44 (36.30 to 38.58) | 39.27 (38.10 to 40.45) | 40.29 (39.13 to 41.45) | 41.77 (40.60 to 42.94) | 43.55 (42.39 to 44.71) | 42.66 (41.62 to 43.70) | 43.06 (42.04 to 44.07) | 45.00 (44.03 to 45.97) |
| **Economic level of family** |  |  |  |  |  |  |  |  |  |  |  |  |  |  |  |  |
| Low | Hypertension | 33.02 (32.95 to 33.10) | 23.38 (23.15 to 23.61) | 25.70 (25.45 to 25.95) | 28.06 (27.80 to 28.32) | 29.19 (28.92 to 29.46) | 31.25 (30.97 to 31.53) | 30.19 (29.93 to 30.44) | 31.98 (31.71 to 32.24) | 33.55 (33.28 to 33.82) | 35.66 (35.39 to 35.94) | 39.96 (39.62 to 40.30) | 41.82 (41.49 to 42.16) | 39.58 (39.26 to 39.91) | 41.22 (40.88 to 41.55) | 43.92 (43.58 to 44.26) |
|  | Treatment | 31.23 (31.15 to 31.31) | 21.78 (21.56 to 22.00) | 23.56 (23.32 to 23.80) | 25.91 (25.65 to 26.16) | 27.28 (27.01 to 27.54) | 29.16 (28.88 to 29.43) | 27.92 (27.67 to 28.17) | 29.62 (29.36 to 29.87) | 31.26 (30.99 to 31.53) | 33.32 (33.04 to 33.59) | 38.75 (38.41 to 39.09) | 40.75 (40.41 to 41.08) | 38.75 (38.43 to 39.08) | 40.39 (40.06 to 40.73) | 43.06 (42.72 to 43.40) |
| High | Hypertension | 15.95 (15.89 to 16.02) | 12.89 (12.66 to 13.12) | 13.29 (13.06 to 13.53) | 13.37 (13.14 to 13.59) | 13.75 (13.54 to 13.97) | 14.43 (14.21 to 14.64) | 14.66 (14.43 to 14.89) | 15.09 (14.87 to 15.32) | 15.26 (15.04 to 15.48) | 16.48 (16.25 to 16.70) | 16.86 (16.62 to 17.10) | 18.35 (18.10 to 18.60) | 18.10 (17.85 to 18.34) | 19.10 (18.84 to 19.35) | 20.66 (20.41 to 20.91) |
|  | Treatment | 14.45 (14.39 to 14.51) | 11.51 (11.29 to 11.72) | 11.32 (11.11 to 11.54) | 11.35 (11.14 to 11.56) | 12.05 (11.85 to 12.26) | 12.62 (12.41 to 12.82) | 12.58 (12.37 to 12.79) | 12.95 (12.74 to 13.17) | 13.27 (13.06 to 13.48) | 14.36 (14.15 to 14.58) | 16.08 (15.85 to 16.31) | 17.46 (17.22 to 17.70) | 17.39 (17.15 to 17.64) | 18.36 (18.11 to 18.61) | 19.92 (19.68 to 20.17) |
| **Smoking status** |  |  |  |  |  |  |  |  |  |  |  |  |  |  |  |  |
| No | Hypertension | 26.32 (26.27 to 26.38) | 21.04 (20.85 to 21.24) | 22.12 (21.92 to 22.32) | 23.28 (23.08 to 23.48) | 23.49 (23.29 to 23.69) | 24.57 (24.37 to 24.78) | 24.81 (24.60 to 25.01) | 25.82 (25.62 to 26.02) | 26.54 (26.33 to 26.74) | 27.81 (27.60 to 28.02) | 28.17 (27.96 to 28.39) | 29.97 (29.76 to 30.19) | 28.52 (28.31 to 28.72) | 29.53 (29.33 to 29.74) | 31.25 (31.05 to 31.46) |
|  | Treatment | 24.88 (24.82 to 24.93) | 19.70 (19.51 to 19.89) | 20.27 (20.08 to 20.46) | 21.41 (21.22 to 21.61) | 21.85 (21.65 to 22.05) | 22.75 (22.55 to 22.95) | 22.83 (22.63 to 23.03) | 23.80 (23.60 to 24.00) | 24.57 (24.37 to 24.77) | 25.80 (25.59 to 26.00) | 27.29 (27.08 to 27.49) | 29.06 (28.85 to 29.27) | 27.83 (27.62 to 28.03) | 28.82 (28.62 to 29.03) | 30.50 (30.30 to 30.71) |
| Yes | Hypertension | 18.47 (18.37 to 18.56) | 13.77 (13.48 to 14.06) | 15.04 (14.72 to 15.35) | 15.82 (15.50 to 16.15) | 16.06 (15.73 to 16.40) | 16.94 (16.59 to 17.28) | 17.99 (17.64 to 18.34) | 18.73 (18.35 to 19.11) | 18.71 (18.33 to 19.08) | 20.34 (19.94 to 20.73) | 20.09 (19.68 to 20.49) | 21.27 (20.85 to 21.69) | 21.51 (21.09 to 21.93) | 22.87 (22.44 to 23.30) | 24.21 (23.78 to 24.65) |
|  | Treatment | 16.15 (16.06 to 16.24) | 11.71 (11.44 to 11.98) | 12.24 (11.96 to 12.53) | 12.95 (12.65 to 13.25) | 13.55 (13.24 to 13.86) | 14.41 (14.09 to 14.73) | 15.03 (14.70 to 15.36) | 15.39 (15.05 to 15.74) | 15.74 (15.39 to 16.09) | 17.06 (16.69 to 17.43) | 18.74 (18.35 to 19.13) | 19.88 (19.47 to 20.29) | 20.24 (19.83 to 20.65) | 21.56 (21.13 to 21.98) | 23.06 (22.63 to 23.48) |
| **Alcohol consumption frequency, days/month** |  |  |  |  |  |  |  |  |  |  |  |  |  |  |  |  |
| 0 | Hypertension | 31.03 (30.95 to 31.10) | 24.04 (23.78 to 24.29) | 25.37 (25.10 to 25.63) | 26.83 (26.56 to 27.10) | 27.05 (26.78 to 27.32) | 28.44 (28.17 to 28.72) | 29.16 (28.88 to 29.44) | 30.14 (29.86 to 30.43) | 31.14 (30.85 to 31.42) | 32.80 (32.51 to 33.08) | 32.98 (32.69 to 33.27) | 34.80 (34.51 to 35.08) | 35.40 (35.11 to 35.69) | 36.63 (36.34 to 36.91) | 39.54 (39.24 to 39.84) |
|  | Treatment | 29.57 (29.50 to 29.65) | 22.75 (22.50 to 22.99) | 23.63 (23.37 to 23.88) | 25.01 (24.75 to 25.28) | 25.47 (25.20 to 25.73) | 26.64 (26.37 to 26.91) | 27.17 (26.89 to 27.45) | 28.15 (27.87 to 28.43) | 29.17 (28.89 to 29.45) | 30.76 (30.48 to 31.04) | 31.98 (31.69 to 32.27) | 33.86 (33.58 to 34.14) | 34.62 (34.33 to 34.90) | 35.89 (35.60 to 36.17) | 38.75 (38.45 to 39.05) |
| <5 | Hypertension | 16.35 (16.27 to 16.42) | 12.24 (11.99 to 12.48) | 13.15 (12.90 to 13.40) | 13.86 (13.60 to 14.13) | 13.83 (13.57 to 14.10) | 14.85 (14.58 to 15.12) | 15.23 (14.96 to 15.50) | 16.36 (16.08 to 16.64) | 16.14 (15.86 to 16.42) | 17.48 (17.19 to 17.77) | 17.79 (17.50 to 18.09) | 18.97 (18.67 to 19.28) | 19.82 (19.47 to 20.17) | 19.82 (19.47 to 20.17) | 22.41 (22.07 to 22.76) |
|  | Treatment | 14.89 (14.82 to 14.97) | 10.96 (10.72 to 11.19) | 11.29 (11.06 to 11.53) | 12.08 (11.84 to 12.33) | 12.19 (11.94 to 12.44) | 13.15 (12.89 to 13.41) | 13.33 (13.07 to 13.58) | 14.34 (14.08 to 14.60) | 14.31 (14.04 to 14.57) | 15.51 (15.24 to 15.79) | 17.06 (16.77 to 17.36) | 18.16 (17.86 to 18.46) | 19.12 (18.78 to 19.47) | 19.03 (18.68 to 19.37) | 21.66 (21.32 to 22.00) |
| ≥5 | Hypertension | 22.70 (22.60 to 22.79) | 18.47 (18.11 to 18.82) | 20.30 (19.93 to 20.67) | 21.42 (21.06 to 21.79) | 21.97 (21.59 to 22.35) | 22.64 (22.27 to 23.02) | 23.05 (22.68 to 23.42) | 24.24 (23.86 to 24.61) | 24.75 (24.37 to 25.14) | 25.38 (25.00 to 25.76) | 25.44 (25.05 to 25.83) | 26.63 (26.23 to 27.04) | 20.41 (20.11 to 20.71) | 21.32 (21.00 to 21.63) | 22.81 (22.50 to 23.12) |
|  | Treatment | 20.56 (20.47 to 20.66) | 16.06 (15.73 to 16.40) | 17.16 (16.81 to 17.50) | 18.34 (18.00 to 18.69) | 19.34 (18.98 to 19.70) | 19.96 (19.60 to 20.32) | 20.10 (19.75 to 20.45) | 21.07 (20.72 to 21.43) | 21.75 (21.38 to 22.12) | 22.34 (21.97 to 22.71) | 24.21 (23.83 to 24.59) | 25.24 (24.84 to 25.64) | 19.55 (19.25 to 19.84) | 20.38 (20.07 to 20.68) | 21.91 (21.60 to 22.21) |
| **BMI group** |  |  |  |  |  |  |  |  |  |  |  |  |  |  |  |  |
| Normal | Hypertension | 17.69 (17.62 to 17.75) | 13.84 (13.65 to 14.04) | 14.64 (14.43 to 14.84) | 15.61 (15.40 to 15.82) | 15.66 (15.44 to 15.87) | 16.73 (16.51 to 16.95) | 16.74 (16.52 to 16.96) | 17.55 (17.33 to 17.78) | 18.28 (18.05 to 18.51) | 19.37 (19.13 to 19.61) | 18.48 (18.23 to 18.73) | 18.70 (18.44 to 18.95) | 20.10 (19.86 to 20.35) | 21.13 (20.88 to 21.39) | 22.52 (22.26 to 22.77) |
|  | Treatment | 16.43 (16.37 to 16.49) | 12.71 (12.52 to 12.90) | 13.16 (12.97 to 13.36) | 14.07 (13.86 to 14.27) | 14.28 (14.08 to 14.49) | 15.19 (14.98 to 15.41) | 15.13 (14.92 to 15.34) | 15.88 (15.66 to 16.10) | 16.59 (16.36 to 16.81) | 17.66 (17.43 to 17.90) | 17.64 (17.40 to 17.89) | 17.91 (17.66 to 18.16) | 19.44 (19.20 to 19.68) | 20.52 (20.27 to 20.76) | 21.86 (21.60 to 22.11) |
| Overweight to obese | Hypertension | 31.46 (31.39 to 31.53) | 25.56 (25.29 to 25.82) | 27.09 (26.82 to 27.36) | 28.27 (27.99 to 28.54) | 28.51 (28.24 to 28.79) | 29.52 (29.24 to 29.79) | 30.20 (29.92 to 30.47) | 31.23 (30.95 to 31.50) | 31.36 (31.09 to 31.64) | 32.93 (32.66 to 33.20) | 33.16 (32.89 to 33.43) | 35.37 (35.11 to 35.64) | 33.27 (33.01 to 33.53) | 34.48 (34.22 to 34.75) | 36.30 (36.03 to 36.57) |
|  | Treatment | 29.51 (29.43 to 29.58) | 23.60 (23.34 to 23.85) | 24.37 (24.11 to 24.63) | 25.58 (25.32 to 25.85) | 26.20 (25.94 to 26.47) | 27.10 (26.83 to 27.37) | 27.42 (27.16 to 27.69) | 28.38 (28.11 to 28.64) | 28.77 (28.51 to 29.04) | 30.20 (29.94 to 30.47) | 32.08 (31.82 to 32.35) | 34.24 (33.98 to 34.50) | 32.38 (32.12 to 32.64) | 33.52 (33.25 to 33.78) | 35.35 (35.09 to 35.62) |
| **Depression status** |  |  |  |  |  |  |  |  |  |  |  |  |  |  |  |  |
| No | Hypertension | 24.48 (24.43 to 24.53) | 18.83 (18.66 to 19.00) | 20.19 (20.02 to 20.36) | 21.42 (21.24 to 21.60) | 21.59 (21.41 to 21.77) | 22.65 (22.47 to 22.83) | 23.00 (22.81 to 23.18) | 24.14 (23.95 to 24.32) | 24.78 (24.59 to 24.97) | 26.24 (26.05 to 26.43) | 26.38 (26.19 to 26.58) | 28.22 (28.02 to 28.41) | 27.23 (27.04 to 27.42) | 28.16 (27.96 to 28.35) | 29.66 (29.46 to 29.85) |
|  | Treatment | 22.92 (22.87 to 22.96) | 17.36 (17.20 to 17.53) | 18.18 (18.02 to 18.35) | 19.41 (19.24 to 19.58) | 19.81 (19.64 to 19.98) | 20.74 (20.56 to 20.91) | 20.88 (20.70 to 21.06) | 21.98 (21.80 to 22.16) | 22.68 (22.50 to 22.87) | 24.04 (23.86 to 24.23) | 25.46 (25.27 to 25.65) | 27.26 (27.06 to 27.45) | 26.47 (26.28 to 26.66) | 27.39 (27.20 to 27.58) | 28.87 (28.68 to 29.07) |
| Yes | Hypertension | 29.61 (29.41 to 29.82) | 25.02 (24.37 to 25.68) | 26.14 (25.36 to 26.93) | 25.99 (25.17 to 26.81) | 27.37 (26.53 to 28.21) | 28.19 (27.39 to 28.98) | 28.67 (27.94 to 29.39) | 29.47 (28.71 to 30.22) | 29.21 (28.44 to 29.99) | 29.95 (29.18 to 30.72) | 32.15 (31.32 to 32.98) | 33.21 (32.41 to 34.01) | 29.43 (28.64 to 30.23) | 32.24 (31.51 to 32.96) | 35.38 (34.67 to 36.09) |
|  | Treatment | 27.22 (27.02 to 27.42) | 22.93 (22.29 to 23.57) | 23.08 (22.33 to 23.83) | 22.50 (21.72 to 23.28) | 24.72 (23.91 to 25.53) | 25.33 (24.56 to 26.10) | 25.54 (24.85 to 26.24) | 25.63 (24.91 to 26.35) | 26.10 (25.35 to 26.85) | 26.97 (26.23 to 27.72) | 30.42 (29.60 to 31.24) | 31.70 (30.91 to 32.50) | 28.18 (27.40 to 28.97) | 30.94 (30.22 to 31.65) | 34.14 (33.43 to 34.84) |
| **Educational background** |  |  |  |  |  |  |  |  |  |  |  |  |  |  |  |  |
| High school or less | Hypertension | 33.00 (32.93 to 33.06) | 24.84 (24.62 to 25.05) | 26.65 (26.42 to 26.88) | 28.16 (27.93 to 28.39) | 28.89 (28.65 to 29.12) | 30.46 (30.22 to 30.70) | 31.38 (31.14 to 31.63) | 32.69 (32.44 to 32.94) | 34.07 (33.82 to 34.32) | 35.73 (35.47 to 35.98) | 36.42 (36.17 to 36.68) | 37.96 (37.71 to 38.22) | 37.04 (36.78 to 37.29) | 38.80 (38.54 to 39.06) | 40.70 (40.44 to 40.96) |
|  | Treatment | 31.31 (31.25 to 31.38) | 23.19 (22.97 to 23.40) | 24.53 (24.31 to 24.75) | 26.01 (25.78 to 26.23) | 27.01 (26.78 to 27.25) | 28.40 (28.16 to 28.64) | 29.11 (28.87 to 29.35) | 30.41 (30.16 to 30.65) | 31.78 (31.53 to 32.02) | 33.43 (33.18 to 33.68) | 35.33 (35.07 to 35.59) | 36.93 (36.68 to 37.19) | 36.23 (35.97 to 36.48) | 38.02 (37.77 to 38.28) | 39.88 (39.63 to 40.14) |
| College or more | Hypertension | 10.38 (10.32 to 10.43) | 7.15 (6.96 to 7.34) | 8.26 (8.06 to 8.46) | 8.61 (8.41 to 8.82) | 8.66 (8.46 to 8.86) | 9.22 (9.01 to 9.42) | 9.53 (9.33 to 9.73) | 10.28 (10.07 to 10.50) | 10.03 (9.83 to 10.24) | 10.99 (10.78 to 11.21) | 10.78 (10.57 to 11.00) | 11.77 (11.54 to 11.99) | 11.78 (11.56 to 11.99) | 12.74 (12.52 to 12.96) | 13.74 (13.51 to 13.96) |
|  | Treatment | 8.88 (8.83 to 8.93) | 5.94 (5.76 to 6.11) | 6.30 (6.12 to 6.48) | 6.65 (6.47 to 6.83) | 6.92 (6.74 to 7.10) | 7.42 (7.23 to 7.60) | 7.49 (7.31 to 7.68) | 8.04 (7.85 to 8.23) | 8.10 (7.91 to 8.29) | 8.84 (8.65 to 9.04) | 10.01 (9.81 to 10.22) | 10.83 (10.61 to 11.04) | 11.02 (10.81 to 11.23) | 11.88 (11.66 to 12.09) | 12.91 (12.69 to 13.13) |
| **Occupation status** |  |  |  |  |  |  |  |  |  |  |  |  |  |  |  |  |
| White-collar | Hypertension | 10.78 (10.70 to 10.85) | 8.75 (8.48 to 9.01) | 9.30 (9.01 to 9.58) | 9.56 (9.28 to 9.85) | 9.31 (9.03 to 9.58) | 9.61 (9.33 to 9.89) | 10.26 (9.98 to 10.54) | 10.45 (10.16 to 10.74) | 10.50 (10.22 to 10.79) | 11.57 (11.28 to 11.87) | 10.90 (10.61 to 11.19) | 11.91 (11.60 to 12.23) | 12.17 (11.86 to 12.48) | 12.64 (12.34 to 12.94) | 13.35 (13.04 to 13.65) |
|  | Treatment | 9.20 (9.12 to 9.27) | 7.26 (7.02 to 7.51) | 7.12 (6.87 to 7.37) | 7.46 (7.20 to 7.71) | 7.47 (7.22 to 7.72) | 7.68 (7.43 to 7.94) | 8.11 (7.85 to 8.36) | 8.11 (7.85 to 8.36) | 8.49 (8.23 to 8.75) | 9.39 (9.12 to 9.66) | 10.14 (9.86 to 10.43) | 11.03 (10.73 to 11.33) | 11.43 (11.13 to 11.73) | 11.77 (11.47 to 12.06) | 12.58 (12.28 to 12.87) |
| Blue-collar | Hypertension | 24.56 (24.49 to 24.64) | 17.54 (17.30 to 17.78) | 19.03 (18.77 to 19.29) | 20.61 (20.35 to 20.86) | 21.41 (21.15 to 21.67) | 22.61 (22.35 to 22.88) | 22.74 (22.47 to 23.00) | 24.34 (24.06 to 24.61) | 24.95 (24.67 to 25.22) | 26.64 (26.36 to 26.92) | 26.74 (26.45 to 27.03) | 28.46 (28.18 to 28.75) | 27.67 (27.38 to 27.95) | 29.55 (29.26 to 29.84) | 31.10 (30.81 to 31.39) |
|  | Treatment | 22.83 (22.76 to 22.90) | 15.82 (15.58 to 16.05) | 16.76 (16.51 to 17.01) | 18.34 (18.09 to 18.58) | 19.51 (19.26 to 19.76) | 20.56 (20.31 to 20.82) | 20.38 (20.13 to 20.64) | 21.86 (21.60 to 22.12) | 22.67 (22.40 to 22.93) | 24.30 (24.03 to 24.57) | 25.73 (25.45 to 26.02) | 27.44 (27.16 to 27.73) | 26.81 (26.52 to 27.09) | 28.74 (28.45 to 29.02) | 30.23 (29.94 to 30.52) |
| Unemployed | Hypertension | 32.55 (32.46 to 32.64) | 26.29 (26.00 to 26.59) | 26.70 (26.41 to 26.98) | 29.26 (28.94 to 29.58) | 29.59 (29.27 to 29.92) | 30.87 (30.55 to 31.20) | 31.46 (31.14 to 31.78) | 32.62 (32.29 to 32.96) | 33.54 (33.20 to 33.87) | 34.69 (34.36 to 35.03) | 35.40 (35.06 to 35.73) | 36.91 (36.58 to 37.24) | 34.51 (34.19 to 34.82) | 36.08 (35.75 to 36.41) | 38.43 (38.10 to 38.76) |
|  | Treatment | 31.05 (30.97 to 31.14) | 24.99 (24.70 to 25.28) | 24.87 (24.59 to 25.14) | 27.40 (27.09 to 27.71) | 27.86 (27.54 to 28.18) | 28.98 (28.66 to 29.30) | 29.46 (29.14 to 29.77) | 30.65 (30.33 to 30.98) | 31.45 (31.12 to 31.77) | 32.54 (32.21 to 32.86) | 34.35 (34.02 to 34.69) | 35.89 (35.56 to 36.22) | 33.77 (33.46 to 34.08) | 35.32 (35.00 to 35.64) | 37.64 (37.32 to 37.97) |
| **Marital status** |  |  |  |  |  |  |  |  |  |  |  |  |  |  |  |  |
| Married | Hypertension | 25.41 (25.35 to 25.47) | 19.49 (19.29 to 19.68) | 20.90 (20.70 to 21.10) | 22.06 (21.86 to 22.27) | 22.43 (22.23 to 22.64) | 23.63 (23.41 to 23.84) | 24.03 (23.82 to 24.24) | 25.06 (24.85 to 25.28) | 25.69 (25.48 to 25.91) | 27.19 (26.96 to 27.41) | 27.66 (27.43 to 27.89) | 29.03 (28.80 to 29.26) | 28.74 (28.51 to 28.98) | 29.56 (29.33 to 29.79) | 31.27 (31.04 to 31.51) |
|  | Treatment | 23.76 (23.70 to 23.82) | 17.91 (17.73 to 18.10) | 18.72 (18.53 to 18.91) | 19.91 (19.71 to 20.10) | 20.55 (20.35 to 20.75) | 21.63 (21.43 to 21.84) | 21.81 (21.60 to 22.01) | 22.75 (22.54 to 22.96) | 23.55 (23.34 to 23.76) | 24.94 (24.72 to 25.16) | 26.69 (26.46 to 26.91) | 28.03 (27.80 to 28.26) | 27.96 (27.73 to 28.19) | 28.80 (28.57 to 29.03) | 30.48 (30.25 to 30.71) |
| Unmarried | Hypertension | 23.37 (23.29 to 23.46) | 18.83 (18.53 to 19.13) | 19.53 (19.22 to 19.85) | 20.57 (20.24 to 20.89) | 20.41 (20.08 to 20.73) | 21.28 (20.95 to 21.61) | 21.82 (21.50 to 22.14) | 23.08 (22.76 to 23.41) | 23.55 (23.23 to 23.88) | 24.82 (24.49 to 25.15) | 24.51 (24.18 to 24.84) | 27.43 (27.10 to 27.76) | 24.64 (24.33 to 24.95) | 26.24 (25.93 to 26.56) | 27.77 (27.45 to 28.08) |
|  | Treatment | 21.85 (21.76 to 21.93) | 17.45 (17.16 to 17.74) | 17.77 (17.47 to 18.07) | 18.65 (18.34 to 18.97) | 18.73 (18.41 to 19.04) | 19.39 (19.07 to 19.70) | 19.73 (19.42 to 20.04) | 20.92 (20.60 to 21.24) | 21.36 (21.05 to 21.68) | 22.59 (22.27 to 22.91) | 23.54 (23.21 to 23.87) | 26.45 (26.12 to 26.78) | 23.84 (23.54 to 24.15) | 25.33 (25.02 to 25.64) | 26.90 (26.58 to 27.21) |

BMI, body mass index; CI, confidence interval.
