# Supplementary material for: National and Regional Trends in the Prevalence of Hypertension in South Korea Amid the Pandemic, 2009-2022: Nationwide Study of Over 3 Million Individuals
Source: JMIR Public Health Surveill. 2024 Jul 30;10:e51891. doi: 10.2196/51891 (PMC11322715; doi:10.2196/51891)
Supplement: Multimedia Appendix 2 [file publichealth_v10i1e51891_app2.docx]

**Table S1.** General characteristics of South Korean adults, 2009-2022 (N=3,072,546).

| Variables | | Total, n (%) | 2009 (n=224,020), n (%) | 2010 (n=219,000), n (%) | 2011 (n=217,096), n (%) | 2012 (n=215,417), n (%) | 2013 (n=215,023), n (%) | 2014 (n=217,601), n (%) | 2015 (n=217,513), n (%) | 2016 (n=218,081), n (%) | 2017 (n=219,778), n (%) | 2018 (n=212,258), n (%) | 2019 (n=218,624), n (%) | 2020 (n=224,185), n (%) | 2021 (n=225,319), n (%) | 2022 (n=228,631), n (%) |  |
| --- | --- | --- | --- | --- | --- | --- | --- | --- | --- | --- | --- | --- | --- | --- | --- | --- | --- |
|  | | | | | | | | | | | | | | | | | |
| **Age (years)** | | | | | | | | | | | | | | | | | |
|  | 19-39 | 794,239 (25.85) | 71,681 (32.00) | 67,391 (30.77) | 63,082 (29.06) | 61,000 (28.32) | 58,513 (27.21) | 59,449 (27.32) | 56,453 (25.95) | 56,099 (25.72) | 53,601 (24.39) | 50,798 (23.93) | 47,346 (21.66) | 51,176 (22.83) | 50,220 (22.29) | 47,430 (20.75) |  |
|  | 40-59 | 1,179,388 (38.38) | 88,629 (39.56) | 87,630 (40.01) | 88,801 (40.90) | 88,943 (41.29) | 87,871 (40.87) | 88,672 (40.75) | 86,329 (39.69) | 85,815 (39.35) | 83,720 (38.09) | 79,990 (37.69) | 77,164 (35.30) | 80,079 (35.72) | 78,280 (34.74) | 77,465 (33.88) |  |
|  | 60-79 | 948,097 (30.86) | 56,978 (25.43) | 57,429 (26.22) | 58,637 (27.01) | 58,911 (27.35) | 61,423 (28.57) | 61,502 (28.26) | 65,709 (30.21) | 65,882 (30.21) | 70,620 (32.13) | 70,296 (33.12) | 78,227 (35.78) | 77,598 (34.61) | 80,060 (35.53) | 84,825 (37.10) |  |
|  | ≥80 | 150,822 (4.91) | 6732 (3.01) | 6550 (2.99) | 6576 (3.03) | 6563 (3.05) | 7216 (3.36) | 7978 (3.67) | 9022 (4.15) | 10,285 (4.72) | 11,837 (5.39) | 11,174 (5.26) | 15,887 (7.27) | 15,332 (6.84) | 16,759 (7.44) | 18,911 (8.27) |  |
| **Sex** | | | | | | | | | | | | | | | | | |
|  | Men | 1,426,379 (46.42) | 105,587 (47.13) | 102,590 (46.84) | 100,840 (46.45) | 100,612 (46.71) | 100,449 (46.72) | 101,781 (46.77) | 101,251 (46.55) | 101,410 (46.50) | 101,264 (46.08) | 98,959 (46.62) | 98,966 (45.27) | 103,186 (46.03) | 103,900 (46.11) | 105,584 (46.18) |  |
|  | Women | 1,646,167 (53.58) | 118,433 (52.87) | 116,410 (53.16) | 116,256 (53.55) | 114,805 (53.29) | 114,574 (53.28) | 115,820 (53.23) | 116,262 (53.45) | 116,671 (53.50) | 118,514 (53.92) | 113,299 (53.38) | 119,658 (54.73) | 120,999 (53.97) | 121,419 (53.89) | 123,047 (53.82) |  |
| **Region of residence** | | | | | | | | | | | | | | | | | |
|  | Urban | 1,512,260 (49.22) | 109,448 (48.86) | 106,792 (48.76) | 106,511 (49.06) | 107,255 (49.79) | 107,690 (50.08) | 107,902 (49.59) | 107,815 (49.57) | 107,622 (49.35) | 108,035 (49.16) | 106,600 (50.22) | 105,738 (48.37) | 109,532 (48.86) | 109,740 (48.70) | 111,580 (48.80) |  |
|  | Rural | 1,560,286 (50.78) | 114,572 (51.14) | 112,208 (51.24) | 110,585 (50.94) | 108,162 (50.21) | 107,333 (49.92) | 109,699 (50.41) | 109,698 (50.43) | 110,459 (50.65) | 111,743 (50.84) | 105,658 (49.78) | 112,886 (51.63) | 114,653 (51.14) | 115,579 (51.30) | 117,051 (51.20) |  |
| **Basic livelihood security recipient** | | | | | | | | | | | | | | | | | |
|  | No | 2,966,487 (96.55) | 214,674 (95.83) | 211,034 (96.36) | 210,113 (96.78) | 208,867 (96.96) | 208,875 (97.14) | 210,786 (96.87) | 210,619 (96.83) | 211,438 (96.95) | 212,903 (96.87) | 205,392 (96.77) | 211,632 (96.80) | 215,479 (96.12) | 216,117 (95.92) | 218,558 (95.59) |  |
|  | Yes | 106,059 (3.45) | 9346 (4.17) | 7966 (3.64) | 6983 (3.22) | 6550 (3.04) | 6148 (2.86) | 6815 (3.13) | 6894 (3.17) | 6643 (3.05) | 6875 (3.13) | 6866 (3.23) | 6992 (3.20) | 8706 (3.88) | 9202 (4.08) | 10,073 (4.41) |  |
| **Economic level of the family^a^** | | | | | | | | | | | | | | | | | |
|  | Low | 1,462,162 (47.59) | 133,075 (59.40) | 117,489 (53.65) | 113,688 (52.37) | 107,466 (49.89) | 105,721 (49.17) | 121,356 (55.77) | 120,228 (55.27) | 116,069 (53.22) | 113,672 (51.72) | 79,263 (37.34) | 83,782 (38.32) | 85,824 (38.28) | 83,161 (36.91) | 81,368 (35.59) |  |
|  | High | 1,324,566 (43.11) | 83,186 (37.13) | 82,461 (37.65) | 87,730 (40.41) | 96,381 (44.74) | 102,538 (47.69) | 93,739 (43.08) | 95,307 (43.82) | 99,847 (45.78) | 104,186 (47.41) | 95,654 (45.06) | 95,213 (43.55) | 92,191 (41.12) | 94,149 (41.78) | 101,984 (44.61) |  |
|  | Unknown | 285,818 (9.30) | 7759 (3.46) | 19,050 (8.70) | 15,678 (7.22) | 11,570 (5.37) | 6764 (3.15) | 2506 (1.15) | 1978 (0.91) | 2165 (0.99) | 1920 (0.87) | 37,341 (17.59) | 39,629 (18.13) | 46,170 (20.59) | 48,009 (21.31) | 45,279 (19.80) |  |
| **Smoking status** | | | | | | | | | | | | | | | | | |
|  | No | 2,475,936 (80.58) | 170,116 (75.94) | 169,367 (77.34) | 169,622 (78.13) | 168,834 (78.38) | 169,868 (79.00) | 172,284 (79.17) | 176,531 (81.16) | 176,642 (81.00) | 180,295 (82.04) | 173,867 (81.91) | 182,084 (83.29) | 187,004 (83.42) | 188,613 (83.71) | 190,809 (83.46) |  |
|  | Yes | 596,610 (19.42) | 53,904 (24.06) | 49,633 (22.66) | 47,474 (21.87) | 46,583 (21.62) | 45,155 (21.00) | 45,317 (20.83) | 40,982 (18.84) | 41,439 (19.00) | 39,483 (17.96) | 38,391 (18.09) | 36,540 (16.71) | 37,181 (16.58) | 36,706 (16.29) | 37,822 (16.54) |  |
| **Alcohol consumption frequency (days/month)** | | | | | | | | | | | | | | | | | |
|  | 0 | 1,452,795 (47.28) | 109,858 (49.04) | 105,828 (48.32) | 102,289 (47.12) | 102,713 (47.68) | 101,196 (47.06) | 99,414 (45.69) | 99,654 (45.82) | 101,720 (46.64) | 103,427 (47.06) | 100,285 (47.25) | 109,906 (50.27) | 105,853 (47.22) | 109,804 (48.73) | 100,848 (44.11) |  |
|  | <5 | 890,138 (28.97) | 68,490 (30.57) | 68,363 (31.22) | 66,653 (30.70) | 66,467 (30.86) | 66,391 (30.88) | 68,312 (31.39) | 67,951 (31.24) | 67,904 (31.14) | 66,793 (30.39) | 63,582 (29.96) | 63,280 (28.94) | 49,890 (22.25) | 49,535 (21.98) | 56,527 (24.72) |  |
|  | ≥5 | 729,613 (23.75) | 45,672 (20.39) | 44,809 (20.46) | 48,154 (22.18) | 46,237 (21.46) | 47,436 (22.06) | 49,875 (22.92) | 49,908 (22.94) | 48,457 (22.22) | 49,558 (22.55) | 48,391 (22.80) | 45,438 (20.78) | 68,442 (30.53) | 65,980 (29.28) | 71,256 (31.17) |  |
| **BMI (kg/m^2^) group** | | | | | | | | | | | | | | | | | |
|  | Normal | 1,486,141 (48.37) | 119,743 (53.45) | 115,630 (52.80) | 113,491 (52.28) | 111,095 (51.57) | 110,150 (51.23) | 110,064 (50.58) | 107,269 (49.32) | 105,256 (48.26) | 104,762 (47.67) | 93,244 (43.93) | 89,860 (41.10) | 100,708 (44.92) | 101,865 (45.21) | 103,004 (45.05) |  |
|  | Overweight to obese | 1,586,405 (51.63) | 104,277 (46.55) | 103,370 (47.20) | 103,605 (47.72) | 104,322 (48.43) | 104,873 (48.77) | 107,537 (49.42) | 110,244 (50.68) | 112,825 (51.74) | 115,016 (52.33) | 119,014 (56.07) | 128,764 (58.90) | 123,477 (55.08) | 123,454 (54.79) | 125,627 (54.95) |  |
| **Depression status** | | | | | | | | | | | | | | | | | |
|  | No | 2,882,342 (93.81) | 207,256 (92.52) | 206,963 (94.50) | 206,083 (94.93) | 204,551 (94.96) | 202,681 (94.26) | 202,541 (93.08) | 203,443 (93.53) | 204,871 (93.94) | 206,164 (93.81) | 200,202 (94.32) | 205,417 (93.96) | 211,621 (94.40) | 209,234 (92.86) | 211,315 (92.43) |  |
|  | Yes | 190,204 (6.19) | 16,764 (7.48) | 12,037 (5.50) | 11,013 (5.07) | 10,866 (5.04) | 12,342 (5.74) | 15,060 (6.92) | 14,070 (6.47) | 13,210 (6.06) | 13,614 (6.19) | 12,056 (5.68) | 13,207 (6.04) | 12,564 (5.60) | 16,085 (7.14) | 17,316 (7.57) |  |
| **Educational background** | | | | | | | | | | | | | | | | | |
|  | High school or lower | 1,958,634 (63.75) | 153,801 (68.66) | 145,949 (66.64) | 144,814 (66.71) | 140,829 (65.38) | 139,202 (64.74) | 137,996 (63.42) | 137,832 (63.37) | 136,254 (62.48) | 137,510 (62.57) | 131,847 (62.12) | 139,825 (63.96) | 138,267 (61.68) | 135,833 (60.28) | 138,675 (60.65) |  |
|  | College or higher | 1,113,912 (36.25) | 70,219 (31.34) | 73,051 (33.36) | 72,282 (33.29) | 74,588 (34.62) | 75,821 (35.26) | 79,605 (36.58) | 79,681 (36.63) | 81,827 (37.52) | 82,268 (37.43) | 80,411 (37.88) | 78,799 (36.04) | 85,918 (38.32) | 89,486 (39.72) | 89,956 (39.35) |  |
| **Occupation status** | | | | | | | | | | | | | | | | | |
|  | White-collar | 608,683 (19.81) | 42,239 (18.86) | 40,000 (18.26) | 41,742 (19.23) | 42,489 (19.72) | 42,996 (20.00) | 44,560 (20.48) | 43,609 (20.05) | 44,407 (20.36) | 44,597 (20.29) | 43,154 (20.33) | 40,992 (18.75) | 43,073 (19.21) | 46,970 (20.85) | 47,855 (20.93) |  |
|  | Blue-collar | 1,323,333 (43.07) | 94,473 (42.17) | 85,773 (39.17) | 95,911 (44.18) | 97,662 (45.34) | 95,082 (44.22) | 93,074 (42.77) | 96,959 (44.58) | 96,394 (44.20) | 96,431 (43.88) | 90,859 (42.81) | 95,866 (43.85) | 93,704 (41.80) | 94,785 (42.07) | 96,360 (42.15) |  |
|  | Unemployed | 1,140,530 (37.12) | 87,308 (38.97) | 93,227 (42.57) | 79,443 (36.59) | 75,266 (34.94) | 76,945 (35.78) | 79,967 (36.75) | 76,945 (35.37) | 77,280 (35.44) | 78,750 (35.83) | 78,245 (36.86) | 81,766 (37.40) | 87,408 (38.99) | 83,564 (37.09) | 84,416 (36.92) |  |
| **Marital status** | | | | | | | | | | | | | | | | | |
|  | Married | 2,145,535 (69.83) | 158,506 (70.76) | 157,437 (71.89) | 157,510 (72.55) | 156,887 (72.83) | 154,770 (71.98) | 154,315 (70.92) | 153,775 (70.70) | 152,412 (69.89) | 153,109 (69.67) | 148,401 (69.92) | 148,729 (68.03) | 148,364 (66.18) | 149,844 (66.50) | 151,476 (66.25) |  |
|  | Unmarried | 927,011 (30.17) | 65,514 (29.24) | 61,563 (28.11) | 59,586 (27.45) | 58,530 (27.17) | 60,253 (28.02) | 63,286 (29.08) | 63,738 (29.30) | 65,669 (30.11) | 66,669 (30.33) | 63,857 (30.08) | 69,895 (31.97) | 75,821 (33.82) | 75,475 (33.50) | 77,155 (33.75) |  |
| **Hypertension** | | | | | | | | | | | | | | | | | |
|  | No | 2,310,650 (75.20) | 180,798 (80.71) | 174,071 (79.48) | 170,092 (78.35) | 168,279 (78.12) | 165,636 (77.03) | 166,708 (76.61) | 164,258 (75.52) | 163,453 (74.95) | 161,605 (73.53) | 155,561 (73.29) | 156,274 (71.48) | 162,857 (72.64) | 161,222 (71.55) | 159,836 (69.91) |  |
|  | Yes | 761,896 (24.80) | 43,222 (19.29) | 44,929 (20.52) | 47,004 (21.65) | 47,138 (21.88) | 49,387 (22.97) | 50,893 (23.39) | 53,255 (24.48) | 54,628 (25.05) | 58,173 (26.47) | 56,697 (26.71) | 62,350 (28.52) | 61,328 (27.36) | 64,097 (28.45) | 68,795 (30.09) |  |
| **Hypertension treatment** | | | | | | | | | | | | | | | | | |
|  | No | 2,360,282 (76.82) | 184,192 (82.22) | 178,591 (81.55) | 174,626 (80.44) | 172,214 (79.94) | 169,865 (79.00) | 171,462 (78.80) | 169,196 (77.79) | 168,159 (77.11) | 166,534 (75.77) | 157,622 (74.26) | 158,450 (72.48) | 164,624 (73.43) | 163,042 (72.36) | 161,705 (70.73) |  |
|  | Yes | 712,264 (23.18) | 39,828 (17.78) | 40,409 (18.45) | 42,470 (19.56) | 43,203 (20.06) | 45,158 (21.00) | 46,139 (21.20) | 48,317 (22.21) | 49,922 (22.89) | 53,244 (24.23) | 54,636 (25.74) | 60,174 (27.52) | 59,561 (26.57) | 62,277 (27.64) | 66,926 (29.27) |  |

^a^The economic level of the family was categorized based on monthly household income into 3 groups: low (<3 million won [<US $2176]), high (≥3 million won [>US $2176]), and unknown [32,33].

**Table S2.** Nationwide trends in the prevalence of participants diagnosed with and receiving treatment for hypertension among South Korean adults, 2009-2022 (N=3,072,546).

| Trends | | | Total, weighted % (95% CI) | Before the COVID-19 pandemic, weighted % (95% CI) | | | | | | | | | | | During the COVID-19 pandemic, weighted % (95% CI) | | |
| --- | --- | --- | --- | --- | --- | --- | --- | --- | --- | --- | --- | --- | --- | --- | --- | --- | --- |
|  | | |  | 2009 | 2010 | 2011 | 2012 | 2013 | 2014 | 2015 | 2016 | 2017 | 2018 | 2019 | 2020 | 2021 | 2022 |
|  | | |  |  |  |  |  |  |  |  |  |  |  |  |  |  |  |
| **Overall** | | | | | | | | | | | | | | | | | |
|  | Hypertension | | 19.01 (18.94-19.07) | 15.02 (14.81-15.22) | 15.88 (15.66-16.11) | 16.60 (16.38-16.82) | 16.91 (16.68-17.13) | 17.40 (17.17-17.62) | 18.13 (17.90-18.36) | 18.63 (18.39-18.87) | 19.16 (18.93-19.40) | 19.96 (19.72-20.20) | 19.74 (19.49-19.99) | 20.71 (20.45-20.96) | 20.87 (20.62-21.12) | 21.71 (21.46-21.97) | 22.77 (22.51-23.03) |
|  | Treatment | | 17.39 (17.32-17.45) | 13.49 (13.30-13.68) | 13.75 (13.53-13.96) | 14.43 (14.22-14.63) | 14.96 (14.74-15.17) | 15.38 (15.16-15.59) | 15.88 (15.67-16.10) | 16.23 (16.01-16.46) | 16.99 (16.76-17.21) | 17.67 (17.44-17.90) | 18.82 (18.58-19.06) | 19.66 (19.41-19.91) | 20.01 (19.76-20.25) | 20.83 (20.57-21.08) | 21.90 (21.65-22.15) |
| **Age (years)** | | | | | | | | | | | | | | | | | |
|  | **19-39** | | | | | | | | | | | | | | | | |
|  |  | Hypertension | 2.53 (2.48-2.57) | 1.90 (1.77-2.03) | 2.46 (2.31-2.60) | 2.53 (2.38-2.69) | 2.43 (2.27-2.59) | 2.45 (2.29-2.60) | 2.69 (2.53-2.86) | 2.95 (2.78-3.12) | 2.64 (2.48-2.80) | 3.01 (2.84-3.19) | 1.87 (1.73-2.01) | 2.51 (2.34-2.69) | 2.52 (2.35-2.69) | 2.87 (2.69-3.05) | 2.93 (2.75-3.12) |
|  |  | Treatment | 1.20 (1.17-1.23) | 0.84 (0.76-0.93) | 0.84 (0.76-0.92) | 0.84 (0.75-0.93) | 0.88 (0.79-0.98) | 0.88 (0.80-0.97) | 0.90 (0.81-0.99) | 0.94 (0.85-1.04) | 0.92 (0.82-1.01) | 1.06 (0.96-1.17) | 1.25 (1.14-1.37) | 1.61 (1.47-1.74) | 1.74 (1.60-1.88) | 1.98 (1.83-2.14) | 2.09 (1.94-2.24) |
|  | **40-59** | | | | | | | | | | | | | | | | |
|  |  | Hypertension | 16.87 (16.77-16.96) | 15.12 (14.81-15.44) | 15.90 (15.57-16.24) | 16.37 (16.04-16.69) | 16.36 (16.03-16.68) | 16.41 (16.08-16.75) | 16.65 (16.33-16.98) | 16.83 (16.49-17.16) | 16.75 (16.42-17.08) | 17.39 (17.05-17.73) | 16.99 (16.64-17.34) | 17.00 (16.64-17.36) | 17.66 (17.31-18.01) | 17.94 (17.58-18.29) | 18.32 (17.96-18.69) |
|  |  | Treatment | 15.08 (14.99-15.17) | 13.29 (12.99-13.59) | 13.38 (13.07-13.69) | 13.92 (13.61-14.22) | 14.16 (13.85-14.47) | 14.15 (13.84-14.47) | 14.18 (13.88-14.48) | 14.17 (13.86-14.48) | 14.44 (14.13-14.75) | 14.97 (14.65-15.29) | 16.07 (15.73-16.41) | 15.94 (15.59-16.29) | 16.69 (16.35-17.03) | 16.99 (16.65-17.34) | 17.44 (17.08-17.79) |
|  | **60-79** | | | | | | | | | | | | | | | | |
|  |  | Hypertension | 46.87 (46.72-47.02) | 44.43 (43.83-45.03) | 45.41 (44.81-46.01) | 46.90 (46.31-47.49) | 47.10 (46.50-47.70) | 47.60 (47.02-48.19) | 47.73 (47.16-48.29) | 47.23 (46.67-47.80) | 48.27 (47.71-48.83) | 47.87 (47.32-48.42) | 48.13 (47.60-48.66) | 47.00 (46.50-47.51) | 45.53 (45.01-46.05) | 45.46 (44.96-45.97) | 46.78 (46.30-47.26) |
|  |  | Treatment | 45.10 (44.96-45.25) | 42.50 (41.90-43.10) | 42.96 (42.36-43.56) | 44.29 (43.69-44.88) | 44.83 (44.23-45.43) | 45.26 (44.67-45.85) | 45.12 (44.55-45.68) | 44.68 (44.12-45.25) | 45.66 (45.10-46.22) | 45.31 (44.77-45.86) | 46.82 (46.29-47.35) | 45.81 (45.30-46.32) | 44.72 (44.20-45.24) | 44.69 (44.19-45.19) | 45.89 (45.41-46.38) |
|  | **≥80** | | | | | | | | | | | | | | | | |
|  |  | Hypertension | 59.44 (59.06-59.81) | 49.26 (47.54-50.97) | 51.84 (50.08-53.61) | 53.29 (51.47-55.12) | 54.12 (52.28-55.96) | 57.79 (56.10-59.48) | 59.67 (58.10-61.24) | 59.12 (57.61-60.63) | 61.37 (59.98-62.77) | 60.10 (58.77-61.44) | 63.93 (62.64-65.23) | 61.29 (60.14-62.43) | 59.02 (57.85-60.19) | 60.44 (59.33-61.55) | 61.40 (60.40-62.40) |
|  |  | Treatment | 57.64 (57.27-58.02) | 47.06 (45.34-48.78) | 49.35 (47.59-51.12) | 50.60 (48.78-52.43) | 52.11 (50.27-53.95) | 54.85 (53.15-56.56) | 57.10 (55.51-58.69) | 56.60 (55.07-58.13) | 58.36 (56.94-59.78) | 57.47 (56.11-58.83) | 62.11 (60.81-63.41) | 59.95 (58.79-61.12) | 58.10 (56.93-59.28) | 59.37 (58.26-60.48) | 60.64 (59.64-61.65) |
| **Sex** | | | | | | | | | | | | | | | | | |
|  | **Men** | | | | | | | | | | | | | | | | |
|  |  | Hypertension | 19.75 (19.67-19.84) | 14.53 (14.26-14.80) | 15.91 (15.62-16.20) | 16.87 (16.58-17.17) | 17.29 (16.99-17.59) | 17.87 (17.57-18.18) | 19.02 (18.71-19.33) | 19.73 (19.40-20.05) | 20.05 (19.73-20.36) | 21.28 (20.96-21.61) | 20.74 (20.41-21.07) | 21.44 (21.10-21.78) | 21.77 (21.44-22.10) | 22.98 (22.63-23.32) | 24.16 (23.82-24.51) |
|  |  | Treatment | 17.49 (17.41-17.57) | 12.44 (12.19-12.69) | 12.97 (12.70-13.23) | 13.87 (13.60-14.14) | 14.54 (14.27-14.82) | 15.12 (14.84-15.40) | 15.79 (15.50-16.07) | 16.33 (16.04-16.62) | 17.03 (16.74-17.33) | 18.04 (17.74-18.35) | 19.51 (19.19-19.83) | 19.97 (19.64-20.29) | 20.56 (20.24-20.88) | 21.66 (21.32-21.99) | 22.93 (22.60-23.26) |
|  | **Women** | | | | | | | | | | | | | | | | |
|  |  | Hypertension | 18.25 (18.17-18.34) | 15.50 (15.23-15.77) | 15.86 (15.56-16.16) | 16.32 (16.04-16.61) | 16.51 (16.22-16.81) | 16.91 (16.62-17.20) | 17.23 (16.94-17.52) | 17.51 (17.21-17.81) | 18.27 (17.97-18.58) | 18.63 (18.32-18.93) | 18.73 (18.42-19.04) | 19.97 (19.65-20.29) | 19.98 (19.66-20.29) | 20.46 (20.14-20.78) | 21.39 (21.07-21.71) |
|  |  | Treatment | 17.29 (17.21-17.37) | 14.54 (14.27-14.80) | 14.53 (14.24-14.82) | 14.99 (14.71-15.26) | 15.38 (15.10-15.66) | 15.64 (15.36-15.92) | 15.98 (15.70-16.26) | 16.13 (15.84-16.42) | 16.94 (16.64-17.24) | 17.29 (16.99-17.58) | 18.12 (17.82-18.42) | 19.35 (19.04-19.66) | 19.46 (19.15-19.77) | 20.00 (19.68-20.32) | 20.88 (20.57-21.20) |
| **Region of residence** | | | | | | | | | | | | | | | | | |
|  | **Urban** | | | | | | | | | | | | | | | | |
|  |  | Hypertension | 18.23 (18.15-18.32) | 14.34 (14.09-14.60) | 15.35 (15.07-15.62) | 16.09 (15.82-16.37) | 16.38 (16.10-16.65) | 16.81 (16.53-17.09) | 17.48 (17.20-17.76) | 17.98 (17.68-18.27) | 18.38 (18.09-18.67) | 19.17 (18.88-19.47) | 19.08 (18.78-19.39) | 19.71 (19.40-20.02) | 19.87 (19.57-20.17) | 20.58 (20.27-20.88) | 21.61 (21.30-21.93) |
|  |  | Treatment | 16.59 (16.51-16.66) | 12.81 (12.57-13.05) | 13.14 (12.89-13.40) | 13.88 (13.62-14.13) | 14.35 (14.09-14.62) | 14.74 (14.47-15.00) | 15.21 (14.94-15.47) | 15.52 (15.25-15.80) | 16.19 (15.91-16.47) | 16.80 (16.52-17.08) | 18.19 (17.90-18.48) | 18.65 (18.35-18.95) | 18.98 (18.69-19.28) | 19.68 (19.38-19.98) | 20.71 (20.40-21.01) |
|  | **Rural** | | | | | | | | | | | | | | | | |
|  |  | Hypertension | 20.81 (20.70-20.93) | 16.58 (16.26-16.90) | 17.12 (16.72-17.52) | 17.78 (17.42-18.15) | 18.15 (17.77-18.53) | 18.77 (18.38-19.16) | 19.64 (19.25-20.04) | 20.15 (19.75-20.56) | 21.01 (20.60-21.42) | 21.80 (21.38-22.22) | 21.31 (20.88-21.74) | 23.03 (22.57-23.49) | 23.22 (22.78-23.65) | 24.39 (23.92-24.86) | 25.49 (25.04-25.94) |
|  |  | Treatment | 19.27 (19.15-19.38) | 15.07 (14.76-15.37) | 15.13 (14.74-15.52) | 15.71 (15.36-16.06) | 16.38 (16.01-16.74) | 16.89 (16.51-17.27) | 17.47 (17.09-17.84) | 17.89 (17.51-18.27) | 18.85 (18.46-19.25) | 19.69 (19.29-20.10) | 20.31 (19.89-20.74) | 22.01 (21.56-22.46) | 22.40 (21.98-22.83) | 23.52 (23.06-23.99) | 24.71 (24.27-25.15) |
| **Basic livelihood security recipient** | | | | | | | | | | | | | | | | | |
|  | **No** | | | | | | | | | | | | | | | | |
|  |  | Hypertension | 18.52 (18.45-18.58) | 14.53 (14.32-14.73) | 15.51 (15.29-15.74) | 16.27 (16.05-16.49) | 16.54 (16.32-16.77) | 17.02 (16.79-17.25) | 17.72 (17.49-17.95) | 18.23 (17.99-18.47) | 18.69 (18.45-18.92) | 19.51 (19.26-19.75) | 19.25 (19.01-19.50) | 20.22 (19.96-20.47) | 20.21 (19.97-20.46) | 21.10 (20.84-21.35) | 22.03 (21.78-22.29) |
|  |  | Treatment | 16.91 (16.85-16.98) | 13.02 (12.83-13.21) | 13.39 (13.17-13.60) | 14.09 (13.88-14.30) | 14.61 (14.39-14.82) | 15.01 (14.80-15.23) | 15.48 (15.27-15.70) | 15.85 (15.62-16.07) | 16.54 (16.31-16.77) | 17.23 (17.00-17.46) | 18.35 (18.11-18.59) | 19.18 (18.93-19.42) | 19.36 (19.12-19.60) | 20.21 (19.96-20.47) | 21.18 (20.93-21.43) |
|  | **Yes** | | | | | | | | | | | | | | | | |
|  |  | Hypertension | 36.10 (35.67-36.53) | 30.80 (29.40-32.21) | 28.95 (27.43-30.47) | 30.08 (28.55-31.61) | 31.77 (30.17-33.37) | 33.54 (31.89-35.19) | 34.04 (32.41-35.68) | 34.02 (32.36-35.69) | 38.51 (36.80-40.23) | 37.44 (35.81-39.07) | 37.19 (35.57-38.80) | 39.34 (37.68-40.99) | 39.72 (38.25-41.20) | 39.03 (37.64-40.43) | 41.72 (40.36-43.07) |
|  |  | Treatment | 34.10 (33.68-34.52) | 28.61 (27.27-29.96) | 26.46 (24.99-27.94) | 27.89 (26.40-29.39) | 29.27 (27.73-30.82) | 31.00 (29.38-32.62) | 31.52 (29.95-33.08) | 31.12 (29.52-32.73) | 35.16 (33.49-36.84) | 34.69 (33.11-36.28) | 35.65 (34.05-37.24) | 37.88 (36.25-39.51) | 38.54 (37.07-40.02) | 37.98 (36.59-39.37) | 40.38 (39.03-41.73) |
| **Economic level of the family^a^** | | | | | | | | | | | | | | | | | |
|  | **Low** | | | | | | | | | | | | | | | | |
|  |  | Hypertension | 27.09 (26.98-27.20) | 18.45 (18.16-18.75) | 20.47 (20.12-20.82) | 22.33 (21.97-22.69) | 23.41 (23.04-23.79) | 24.79 (24.40-25.17) | 24.44 (24.08-24.79) | 25.39 (25.02-25.77) | 27.21 (26.82-27.60) | 28.73 (28.33-29.13) | 32.70 (32.21-33.19) | 34.17 (33.66-34.68) | 32.81 (32.33-33.29) | 34.17 (33.67-34.66) | 36.74 (36.25-37.24) |
|  |  | Treatment | 25.22 (25.11-25.33) | 16.82 (16.54-17.10) | 18.18 (17.85-18.51) | 20.08 (19.74-20.42) | 21.35 (20.99-21.71) | 22.60 (22.22-22.97) | 22.05 (21.71-22.39) | 22.82 (22.46-23.18) | 24.80 (24.42-25.17) | 26.29 (25.90-26.68) | 31.43 (30.94-31.91) | 32.99 (32.48-33.49) | 31.85 (31.37-32.32) | 33.26 (32.77-33.75) | 35.78 (35.28-36.27) |
|  | **High** | | | | | | | | | | | | | | | | |
|  |  | Hypertension | 13.80 (13.73-13.88) | 11.19 (10.92-11.46) | 11.42 (11.13-11.70) | 11.66 (11.39-11.93) | 12.00 (11.74-12.26) | 12.38 (12.12-12.63) | 12.71 (12.44-12.98) | 13.34 (13.06-13.62) | 13.32 (13.05-13.58) | 14.20 (13.93-14.47) | 14.14 (13.86-14.42) | 15.13 (14.84-15.43) | 15.46 (15.16-15.77) | 16.35 (16.05-16.66) | 17.19 (16.89-17.49) |
|  |  | Treatment | 12.30 (12.22-12.37) | 9.76 (9.51-10.01) | 9.37 (9.11-9.62) | 9.55 (9.30-9.80) | 10.17 (9.92-10.41) | 10.48 (10.24-10.72) | 10.57 (10.32-10.82) | 11.09 (10.83-11.34) | 11.31 (11.07-11.56) | 12.01 (11.76-12.27) | 13.36 (13.09-13.64) | 14.19 (13.91-14.47) | 14.70 (14.41-14.99) | 15.53 (15.23-15.82) | 16.41 (16.12-16.70) |
| **Smoking status** | | | | | | | | | | | | | | | | | |
|  | **No** | | | | | | | | | | | | | | | | |
|  |  | Hypertension | 19.99 (19.92-20.07) | 16.36 (16.12-16.60) | 17.12 (16.85-17.38) | 17.79 (17.53-18.04) | 18.04 (17.78-18.30) | 18.62 (18.36-18.89) | 19.01 (18.75-19.28) | 19.39 (19.13-19.66) | 20.18 (19.91-20.46) | 20.75 (20.47-21.02) | 20.69 (20.42-20.97) | 21.54 (21.26-21.83) | 21.50 (21.22-21.78) | 22.31 (22.03-22.59) | 23.32 (23.04-23.60) |
|  |  | Treatment | 18.58 (18.51-18.66) | 15.02 (14.79-15.25) | 15.23 (14.97-15.48) | 15.89 (15.64-16.13) | 16.35 (16.10-16.60) | 16.80 (16.55-17.05) | 17.05 (16.80-17.30) | 17.34 (17.09-17.60) | 18.25 (17.99-18.51) | 18.74 (18.48-19.00) | 19.86 (19.59-20.13) | 20.62 (20.34-20.90) | 20.75 (20.48-21.02) | 21.53 (21.25-21.81) | 22.55 (22.27-22.83) |
|  | **Yes** | | | | | | | | | | | | | | | | |
|  |  | Hypertension | 15.37 (15.25-15.49) | 11.17 (10.83-11.51) | 12.16 (11.80-12.53) | 12.93 (12.54-13.31) | 13.32 (12.93-13.72) | 13.40 (13.00-13.79) | 15.16 (14.74-15.57) | 15.79 (15.33-16.25) | 15.40 (14.96-15.83) | 16.88 (16.41-17.36) | 16.06 (15.59-16.53) | 17.17 (16.67-17.67) | 18.06 (17.56-18.56) | 18.95 (18.43-19.47) | 20.28 (19.74-20.81) |
|  |  | Treatment | 12.99 (12.87-13.10) | 9.09 (8.79-9.39) | 9.27 (8.95-9.59) | 9.88 (9.55-10.21) | 10.58 (10.23-10.92) | 10.73 (10.38-11.07) | 11.96 (11.59-12.33) | 12.11 (11.71-12.51) | 12.32 (11.93-12.71) | 13.49 (13.07-13.91) | 14.78 (14.33-15.23) | 15.58 (15.10-16.05) | 16.66 (16.18-17.15) | 17.54 (17.03-18.05) | 18.98 (18.46-19.50) |
| **Alcohol consumption frequency (days/month)** | | | | | | | | | | | | | | | | | |
|  | **0** | | | | | | | | | | | | | | | | |
|  |  | Hypertension | 24.63 (24.52-24.73) | 19.25 (18.93-19.58) | 20.12 (19.77-20.47) | 21.13 (20.78-21.49) | 21.57 (21.21-21.93) | 22.23 (21.86-22.60) | 23.11 (22.73-23.48) | 23.36 (22.99-23.74) | 24.47 (24.09-24.85) | 25.47 (25.08-25.85) | 25.32 (24.94-25.70) | 26.22 (25.84-26.61) | 28.81 (28.41-29.22) | 29.65 (29.24-30.06) | 32.39 (31.98-32.81) |
|  |  | Treatment | 23.20 (23.10-23.30) | 17.94 (17.63-18.25) | 18.40 (18.06-18.74) | 19.28 (18.94-19.62) | 19.93 (19.58-20.28) | 20.47 (20.12-20.83) | 21.15 (20.80-21.51) | 21.34 (20.97-21.70) | 22.60 (22.23-22.97) | 23.46 (23.08-23.83) | 24.37 (24.00-24.75) | 25.24 (24.87-25.62) | 27.91 (27.51-28.31) | 28.86 (28.45-29.26) | 31.55 (31.14-31.97) |
|  | **<5** | | | | | | | | | | | | | | | | |
|  |  | Hypertension | 12.56 (12.47-12.65) | 9.41 (9.15-9.68) | 10.24 (9.96-10.53) | 10.81 (10.51-11.10) | 10.70 (10.41-11.00) | 11.33 (11.03-11.63) | 11.99 (11.69-12.29) | 12.59 (12.27-12.91) | 12.55 (12.24-12.86) | 13.25 (12.93-13.57) | 12.98 (12.66-13.30) | 13.74 (13.40-14.07) | 15.25 (14.84-15.65) | 15.02 (14.63-15.41) | 16.66 (16.28-17.04) |
|  |  | Treatment | 11.12 (11.04-11.21) | 8.20 (7.95-8.45) | 8.36 (8.10-8.61) | 8.98 (8.72-9.25) | 9.00 (8.73-9.27) | 9.54 (9.27-9.81) | 10.01 (9.74-10.28) | 10.50 (10.21-10.78) | 10.67 (10.39-10.96) | 11.19 (10.90-11.49) | 12.31 (11.99-12.63) | 12.88 (12.56-13.21) | 14.48 (14.09-14.87) | 14.16 (13.78-14.54) | 15.89 (15.52-16.26) |
|  | **≥5** | | | | | | | | | | | | | | | | |
|  |  | Hypertension | 18.62 (18.50-18.74) | 15.67 (15.23-16.10) | 17.07 (16.61-17.53) | 17.64 (17.20-18.09) | 18.31 (17.85-18.76) | 18.45 (18.00-18.91) | 19.21 (18.76-19.66) | 20.03 (19.56-20.49) | 20.40 (19.94-20.86) | 20.74 (20.27-21.21) | 20.21 (19.73-20.68) | 21.06 (20.57-21.55) | 16.20 (15.86-16.55) | 17.27 (16.89-17.64) | 18.05 (17.69-18.41) |
|  |  | Treatment | 16.47 (16.35-16.58) | 13.15 (12.76-13.55) | 13.68 (13.26-14.09) | 14.35 (13.94-14.75) | 15.38 (14.96-15.81) | 15.62 (15.20-16.04) | 16.06 (15.65-16.47) | 16.56 (16.13-16.98) | 17.23 (16.80-17.66) | 17.63 (17.20-18.07) | 18.95 (18.49-19.41) | 19.57 (19.09-20.04) | 15.31 (14.97-15.64) | 16.24 (15.88-16.60) | 17.08 (16.74-17.43) |
| **BMI (kg/m^2^) group** | | | | | | | | | | | | | | | | | |
|  | **Normal** | | | | | | | | | | | | | | | | |
|  |  | Hypertension | 12.07 (12.00-12.14) | 9.57 (9.36-9.78) | 10.11 (9.87-10.35) | 10.78 (10.54-11.01) | 10.91 (10.68-11.15) | 11.37 (11.13-11.62) | 11.67 (11.42-11.91) | 11.91 (11.65-12.17) | 12.55 (12.28-12.82) | 12.86 (12.59-13.13) | 12.22 (11.94-12.49) | 11.99 (11.72-12.27) | 13.71 (13.43-14.00) | 14.39 (14.10-14.69) | 15.21 (14.91-15.50) |
|  |  | Treatment | 10.96 (10.89-11.03) | 8.54 (8.35-8.74) | 8.76 (8.54-8.98) | 9.31 (9.09-9.53) | 9.61 (9.39-9.84) | 9.97 (9.74-10.20) | 10.24 (10.01-10.47) | 10.36 (10.12-10.60) | 11.06 (10.81-11.31) | 11.37 (11.11-11.62) | 11.52 (11.25-11.78) | 11.30 (11.03-11.57) | 13.08 (12.80-13.35) | 13.80 (13.51-14.08) | 14.63 (14.34-14.91) |
|  | **Overweight-obese** | | | | | | | | | | | | | | | | |
|  |  | Hypertension | 25.57 (25.47-25.66) | 21.46 (21.13-21.80) | 22.59 (22.23-22.95) | 23.19 (22.84-23.55) | 23.49 (23.13-23.85) | 23.90 (23.55-24.26) | 24.98 (24.62-25.33) | 25.39 (25.04-25.75) | 25.48 (25.13-25.83) | 26.58 (26.23-26.93) | 25.90 (25.55-26.25) | 27.20 (26.85-27.55) | 26.73 (26.38-27.07) | 27.76 (27.41-28.12) | 29.03 (28.68-29.39) |
|  |  | Treatment | 23.47 (23.37-23.56) | 19.34 (19.02-19.66) | 19.54 (19.20-19.87) | 20.22 (19.88-20.55) | 20.83 (20.49-21.17) | 21.21 (20.88-21.55) | 21.86 (21.53-22.20) | 22.15 (21.81-22.49) | 22.65 (22.32-22.98) | 23.54 (23.21-23.88) | 24.79 (24.45-25.13) | 25.89 (25.54-26.23) | 25.67 (25.33-26.01) | 26.64 (26.29-26.98) | 27.93 (27.58-28.28) |
| **Depression status** | | | | | | | | | | | | | | | | | |
|  | **No** | | | | | | | | | | | | | | | | |
|  |  | Hypertension | 18.68 (18.61-18.75) | 14.57 (14.36-14.78) | 15.58 (15.35-15.81) | 16.32 (16.10-16.54) | 16.61 (16.38-16.84) | 17.08 (16.85-17.31) | 17.74 (17.51-17.98) | 18.29 (18.05-18.54) | 18.89 (18.65-19.13) | 19.70 (19.46-19.95) | 19.39 (19.13-19.64) | 20.39 (20.13-20.65) | 20.73 (20.48-20.98) | 21.39 (21.13-21.65) | 22.31 (22.04-22.57) |
|  |  | Treatment | 17.12 (17.05-17.18) | 13.10 (12.90-13.29) | 13.51 (13.29-13.72) | 14.22 (14.01-14.43) | 14.70 (14.48-14.91) | 15.13 (14.91-15.35) | 15.57 (15.35-15.79) | 16.01 (15.78-16.24) | 16.78 (16.55-17.01) | 17.46 (17.23-17.70) | 18.51 (18.27-18.76) | 19.37 (19.12-19.63) | 19.89 (19.65-20.14) | 20.56 (20.30-20.81) | 21.49 (21.24-21.75) |
|  | **Yes** | | | | | | | | | | | | | | | | |
|  |  | Hypertension | 23.85 (23.59-24.10) | 20.54 (19.77-21.32) | 20.98 (20.07-21.89) | 21.54 (20.54-22.54) | 22.17 (21.13-23.22) | 22.29 (21.33-23.25) | 23.28 (22.40-24.16) | 23.42 (22.52-24.31) | 23.36 (22.41-24.31) | 23.68 (22.73-24.62) | 25.50 (24.49-26.51) | 25.51 (24.52-26.50) | 23.13 (22.17-24.09) | 25.86 (24.96-26.75) | 28.57 (27.69-29.45) |
|  |  | Treatment | 21.41 (21.17-21.65) | 18.36 (17.61-19.11) | 17.79 (16.94-18.63) | 17.97 (17.04-18.90) | 19.59 (18.60-20.57) | 20.08 (19.26-20.90) | 19.41 (18.59-20.23) | 20.22 (19.32-21.11) | 20.64 (19.75-21.53) | 23.79 (22.81-24.77) | 23.97 (23.01-24.93) | 21.78 (20.85-22.72) | 24.30 (23.42-25.17) | 27.04 (26.18-27.90) | 18.36 (17.61-19.11) |
| **Educational background** | | | | | | | | | | | | | | | | | |
|  | **High school or less** | | | | | | | | | | | | | | | | |
|  |  | Hypertension | 28.45 (28.35-28.54) | 21.35 (21.06-21.64) | 22.90 (22.58-23.22) | 24.08 (23.76-24.40) | 24.92 (24.59-25.25) | 25.91 (25.58-26.25) | 27.10 (26.76-27.44) | 27.86 (27.51-28.22) | 29.29 (28.94-29.65) | 30.40 (30.04-30.76) | 30.73 (30.36-31.09) | 31.66 (31.30-32.03) | 31.70 (31.33-32.07) | 33.27 (32.89-33.64) | 35.04 (34.67-35.41) |
|  |  | Treatment | 26.70 (26.60-26.79) | 19.62 (19.34-19.90) | 20.71 (20.40-21.02) | 21.74 (21.44-22.05) | 22.88 (22.56-23.20) | 23.79 (23.46-24.12) | 24.70 (24.37-25.02) | 25.34 (25.00-25.68) | 26.90 (26.55-27.24) | 28.03 (27.68-28.37) | 29.59 (29.23-29.95) | 30.53 (30.16-30.89) | 30.76 (30.40-31.13) | 32.41 (32.04-32.78) | 34.13 (33.76-34.49) |
|  | **College or more** | | | | | | | | | | | | | | | | |
|  |  | Hypertension | 9.20 (9.13-9.27) | 6.28 (6.05-6.50) | 7.36 (7.11-7.60) | 7.64 (7.40-7.88) | 7.80 (7.56-8.04) | 8.16 (7.91-8.40) | 8.58 (8.34-8.82) | 9.17 (8.91-9.43) | 9.02 (8.78-9.27) | 9.77 (9.52-10.02) | 9.18 (8.93-9.43) | 9.96 (9.70-10.22) | 10.46 (10.21-10.72) | 11.30 (11.04-11.57) | 11.72 (11.46-11.98) |
|  |  | Treatment | 7.72 (7.66-7.79) | 5.02 (4.82-5.22) | 5.28 (5.07-5.48) | 5.65 (5.44-5.87) | 5.96 (5.74-6.17) | 6.25 (6.03-6.47) | 6.49 (6.28-6.70) | 6.90 (6.68-7.13) | 7.07 (6.85-7.29) | 7.56 (7.34-7.78) | 8.46 (8.22-8.70) | 8.99 (8.75-9.24) | 9.66 (9.42-9.91) | 10.38 (10.13-10.64) | 10.89 (10.64-11.15) |
| **Occupation status** | | | | | | | | | | | | | | | | | |
|  | **White-collar** | | | | | | | | | | | | | | | | |
|  |  | Hypertension | 10.00 (9.90-10.09) | 7.89 (7.57-8.20) | 8.66 (8.32-9.00) | 8.95 (8.61-9.29) | 8.69 (8.36-9.02) | 8.92 (8.58-9.26) | 9.70 (9.36-10.04) | 9.81 (9.46-10.15) | 9.90 (9.56-10.24) | 10.75 (10.39-11.10) | 9.89 (9.55-10.24) | 10.67 (10.30-11.05) | 11.25 (10.88-11.62) | 11.47 (11.12-11.82) | 11.88 (11.52-12.23) |
|  |  | Treatment | 8.41 (8.32-8.50) | 6.40 (6.11-6.68) | 6.33 (6.03-6.62) | 6.70 (6.41-7.00) | 6.71 (6.42-7.00) | 6.82 (6.53-7.11) | 7.42 (7.12-7.72) | 7.40 (7.10-7.70) | 7.79 (7.49-8.09) | 8.52 (8.20-8.85) | 9.18 (8.85-9.52) | 9.72 (9.36-10.07) | 10.43 (10.07-10.78) | 10.53 (10.19-10.86) | 11.06 (10.71-11.40) |
|  | **Blue-collar** | | | | | | | | | | | | | | | | |
|  |  | Hypertension | 19.03 (18.94-19.13) | 13.71 (13.41-14.00) | 15.11 (14.78-15.44) | 15.85 (15.53-16.16) | 16.76 (16.43-17.08) | 17.25 (16.92-17.58) | 17.89 (17.55-18.23) | 18.91 (18.56-19.26) | 19.15 (18.80-19.49) | 20.23 (19.88-20.58) | 20.17 (19.80-20.53) | 21.00 (20.63-21.38) | 21.00 (20.63-21.37) | 22.63 (22.25-23.00) | 23.77 (23.39-24.15) |
|  |  | Treatment | 17.19 (17.10-17.29) | 11.92 (11.65-12.19) | 12.73 (12.42-13.03) | 13.41 (13.12-13.70) | 14.61 (14.30-14.91) | 15.05 (14.74-15.36) | 15.29 (14.98-15.60) | 16.09 (15.77-16.41) | 16.71 (16.39-17.04) | 17.65 (17.32-17.98) | 19.13 (18.77-19.49) | 19.82 (19.46-20.18) | 19.98 (19.63-20.34) | 21.65 (21.28-22.03) | 22.78 (22.41-23.16) |
|  | **Unemployed** | | | | | | | | | | | | | | | | |
|  |  | Hypertension | 25.55 (25.43-25.66) | 20.66 (20.29-21.03) | 20.80 (20.42-21.18) | 22.72 (22.32-23.13) | 23.18 (22.76-23.59) | 23.84 (23.42-24.25) | 24.60 (24.18-25.01) | 25.01 (24.59-25.44) | 26.33 (25.90-26.76) | 26.85 (26.41-27.29) | 26.89 (26.46-27.32) | 27.72 (27.28-28.15) | 27.34 (26.92-27.76) | 28.80 (28.36-29.24) | 30.48 (30.05-30.92) |
|  |  | Treatment | 24.13 (24.02-24.25) | 19.35 (19.00-19.71) | 18.98 (18.62-19.34) | 20.88 (20.49-21.26) | 21.48 (21.07-21.88) | 22.07 (21.66-22.47) | 22.73 (22.32-23.13) | 23.09 (22.68-23.49) | 24.37 (23.95-24.79) | 24.82 (24.39-25.24) | 25.92 (25.49-26.34) | 26.74 (26.31-27.17) | 26.59 (26.17-27.00) | 28.03 (27.60-28.47) | 29.70 (29.27-30.14) |
| **Marital status** | | | | | | | | | | | | | | | | | |
|  | **Married** | | | | | | | | | | | | | | | | |
|  |  | Hypertension | 21.08 (20.99-21.16) | 16.32 (16.06-16.58) | 17.48 (17.19-17.77) | 18.34 (18.07-18.62) | 18.80 (18.51-19.08) | 19.26 (18.97-19.54) | 20.09 (19.80-20.37) | 20.64 (20.33-20.94) | 21.15 (20.84-21.45) | 22.13 (21.82-22.44) | 22.18 (21.86-22.50) | 23.01 (22.69-23.34) | 23.61 (23.28-23.94) | 24.32 (23.99-24.65) | 25.56 (25.23-25.90) |
|  |  | Treatment | 19.39 (19.31-19.47) | 14.67 (14.42-14.92) | 15.17 (14.89-15.44) | 16.02 (15.76-16.29) | 16.73 (16.46-17.01) | 17.16 (16.88-17.44) | 17.75 (17.47-18.03) | 18.14 (17.85-18.43) | 18.95 (18.65-19.25) | 19.83 (19.54-20.13) | 21.23 (20.91-21.54) | 21.96 (21.64-22.28) | 22.75 (22.43-23.07) | 23.47 (23.15-23.80) | 24.73 (24.40-25.06) |
|  | **Unmarried** | | | | | | | | | | | | | | | | |
|  |  | Hypertension | 14.93 (14.83-15.02) | 12.33 (12.02-12.64) | 12.48 (12.16-12.80) | 12.78 (12.46-13.11) | 12.74 (12.40-13.07) | 13.43 (13.09-13.77) | 14.08 (13.74-14.42) | 14.43 (14.09-14.78) | 15.19 (14.84-15.54) | 15.68 (15.32-16.03) | 14.97 (14.62-15.31) | 16.41 (16.05-16.77) | 16.13 (15.79-16.47) | 17.11 (16.75-17.47) | 18.03 (17.67-18.38) |
|  |  | Treatment | 13.44 (13.35-13.53) | 11.06 (10.78-11.35) | 10.72 (10.43-11.01) | 10.93 (10.63-11.23) | 11.04 (10.73-11.34) | 11.57 (11.26-11.88) | 12.02 (11.71-12.33) | 12.25 (11.94-12.56) | 13.05 (12.73-13.37) | 13.39 (13.07-13.72) | 14.09 (13.76-14.42) | 15.36 (15.02-15.71) | 15.26 (14.93-15.59) | 16.14 (15.79-16.49) | 17.10 (16.75-17.44) |

^a^The economic level of the family was categorized based on monthly household income into 3 groups: low (<3 million won [<US $2176]), high (≥3 million won [>US $2176]), and unknown [32,33].

**Table S3.** Nationwide trends in the prevalence of participants diagnosed with and receiving treatment for hypertension before (2009-2019) and during the COVID-19 pandemic (2020-2022) among South Korean adults.^a^

| Weighted | | | Trend of before pandemic, β coefficient (95% CI) | Trend of during pandemic, β coefficient (95% CI) | Trend difference, β coefficient (95% CI) | Before and during the pandemic, odds ratio (95% CI)^b^ |
| --- | --- | --- | --- | --- | --- | --- |
| **Overall** | | | | | | |
|  | Hypertension | | *.124 (0.119 to 0.130)* | *.024 (0.020 to 0.027)* | *–.101 (–0.107 to –0.094)* | *1.099 (1.084 to 1.114)* |
|  | Treatment | | *.160 (0.154 to 0.165)* | *.026 (0.023 to 0.030)* | *–.133 (–0.140 to –0.127)* | *1.110 (1.095 to 1.126)* |
| **Sex** | | | | | | |
|  | **Men** | | | | | |
|  |  | Hypertension | *.147 (0.140 to 0.154)* | *.027 (0.023 to 0.032)* | *–.120 (–0.128 to –0.111)* | *1.116 (1.096 to 1.136)* |
|  |  | Treatment | *.199 (0.191 to 0.206)* | *.030 (0.026 to 0.035)* | *–.169 (–0.177 to –0.160)* | *1.129 (1.108 to 1.149)* |
|  | **Women** | | | | | |
|  |  | Hypertension | *.100 (0.093 to 0.107)* | *.020 (0.015 to 0.024)* | *–.080 (–0.089 to –0.072)* | *1.082 (1.063 to 1.101)* |
|  |  | Treatment | *.120 (0.113 to 0.128)* | *.022 (0.017 to 0.026)* | *–.098 (–0.107 to –0.089)* | *1.092 (1.073 to 1.112)* |
| **Age (years)** | | | | | | |
|  | **19-39** | | | | | |
|  |  | Hypertension | .006 (–0. 016 to 0.028) | *.060 (0.044 to 0.076)* | *.054 (0.027 to 0.082)* | *1.274 (1.194 to 1.358)* |
|  |  | Treatment | *.223 (0.187 to 0.258)* | *.077 (0.058 to 0.095)* | *–.146 (–0.186 to –0.106)* | *1.361 (1.260 to 1.470)* |
|  | **40-59** | | | | | |
|  |  | Hypertension | *.036 (0.027 to 0.045)* | *.017 (0.011 to 0.023)* | *–.019 (–0.029 to –0.009)* | *1.070 (1.046 to 1.095)* |
|  |  | Treatment | *.077 (0.068 to 0.086)* | *.019 (0.013 to 0.025)* | *–.058 (–0.069 to –0.047)* | *1.078 (1.054 to 1.103)* |
|  | **60-79** | | | | | |
|  |  | Hypertension | *.028 (0.020 to 0.036)* | *–.016 (–0.021 to –0.011)* | *–.044 (–0.053 to –0.035)* | *.938 (0.921 to 0.956)* |
|  |  | Treatment | *.045 (0.037 to 0.053)* | *–.012 (–0.016 to –0.007)* | *–.057 (–0.066 to –0.048)* | *.954 (0.936 to 0.972)* |
|  | **≥80** | | | | | |
|  |  | Hypertension | *.170 (0.149 to 0.191)* | *–.021 (–0.032 to –0.011)* | *–.191 (–0.214 to –0.168)* | *.916 (0.876 to 0.958)* |
|  |  | Treatment | *.083 (0.162 to 0.204)* | *–.015 (–0.026 to –0.004)* | *–.098 (–0.122 to –0.074)* | *.942 (0.900 to 0.985)* |
| **Region of residence** | | | | | | |
|  | **Urban** | | | | | |
|  |  | Hypertension | *.120 (0.113 to 0.127)* | *.020 (0.016 to 0.024)* | *–.100 (–0.108 to –0.091)* | *1.084 (1.066 to 1.103)* |
|  |  | Treatment | *.158 (0.151 to 0.165)* | *.022 (0.018 to 0.027)* | *–.136 (–0.144 to –0.127)* | *1.093 (1.074 to 1.113)* |
|  | **Rural** | | | | | |
|  |  | Hypertension | *.134 (0.125 to 0.143)* | *.031 (0.025 to 0.036)* | *–.104 (–0.114 to –0.093)* | *1.131 (1.105 to 1.157)* |
|  |  | Treatment | *.164 (0.154 to 0.173)* | *.034 (0.028 to 0.040)* | *–.130 (–0.141 to –0.119)* | *1.147 (1.121 to 1.174)* |
| **Basic livelihood security recipient** | | | | | | |
|  | **No** | | | | | |
|  |  | Hypertension | *.123 (0.118 to 0.129)* | *.021 (0.018 to 0.025)* | *–.102 (–0.109 to –0.096)* | *1.089 (1.074 to 1.104)* |
|  |  | Treatment | *.160 (0.154 to 0.166)* | *.024 (0.020 to 0.027)* | *–.136 (–0.143 to –0.129)* | *1.100 (1.084 to 1.116)* |
|  | **Yes** | | | | | |
|  |  | Hypertension | *.161 (0.135 to 0.187)* | *.020 (0.006 to 0.034)* | *–.141 (–0.170 to –0.112)* | *1.086 (1.024 to 1.153)* |
|  |  | Treatment | *.178 (0.153 to 0.204)* | *.024 (0.009 to 0.038)* | *–.155 (–0.185 to –0.125)* | *1.102 (1.038 to 1.170)* |
| **Economic level of the family^c^** | | | | | | |
|  | **Low** | | | | | |
|  |  | Hypertension | *.251 (0.244 to 0.258)* | *.012 (0.007 to 0.017)* | *–.239 (–0.247 to –0.230)* | *1.050 (1.029 to 1.072)* |
|  |  | Treatment | *.276 (0.268 to 0.283)* | *.016 (0.011 to 0.021)* | *–.260 (–0.269 to –0.251)* | *1.065 (1.043 to 1.086)* |
|  | **High** | | | | | |
|  |  | Hypertension | *.114 (0.106 to 0.122)* | *.033 (0.028 to 0.038)* | *–.081 (–0.091 to –0.071)* | *1.142 (1.118 to 1.165)* |
|  |  | Treatment | *.164 (0.155 to 0.173)* | *.036 (0.031 to 0.041)* | *–.128 (–0.138 to –0.117)* | *1.155 (1.131 to 1.180)* |
| **Smoking status** | | | | | | |
|  | **No** | | | | | |
|  |  | Hypertension | *.108 (0.102 to 0.114)* | *.018 (0.015 to 0.022)* | *–.090 (–0.097 to –0.083)* | *1.077 (1.061 to 1.093)* |
|  |  | Treatment | *.136 (0.130 to 0.142)* | *.021 (0.017 to 0.024)* | *–.115 (–0.123 to –0.108)* | *1.087 (1.070 to 1.103)* |
|  | **Yes** | | | | | |
|  |  | Hypertension | *.157 (0.145 to 0.170)* | *.043 (0.035 to 0.051)* | *–.115 (–0.129 to –0.100)* | *1.187 (1.150 to 1.224)* |
|  |  | Treatment | *.221 (0.208 to 0.234)* | *.047 (0.039 to 0.055)* | *–.174 (–0.189 to –0.159)* | *1.206 (1.168 to 1.245)* |
| **Alcohol consumption frequency (days/month)** | | | | | | |
|  | **0** | | | | | |
|  |  | Hypertension | *.135 (0.127 to 0.142)* | *.055 (0.051 to 0.060)* | *–.080 (–0.088 to –0.071)* | *1.247 (1.225 to 1.270)* |
|  |  | Treatment | *.155 (0.148 to 0.163)* | *.058 (0.053 to 0.062)* | *–.097 (–0.106 to –0.089)* | *1.261 (1.238 to 1.284)* |
|  | **<5** | | | | | |
|  |  | Hypertension | *.129 (0.119 to 0.139)* | *.046 (0.040 to 0.053)* | *–.083 (–0.095 to –0.071)* | *1.206 (1.175 to 1.238)* |
|  |  | Treatment | *.179 (0.168 to 0.190)* | *.048 (0.041 to 0.055)* | *–.131 (–0.144 to –0.118)* | *1.213 (1.181 to 1.246)* |
|  | **≥5** | | | | | |
|  |  | Hypertension | *.102 (0.092 to 0.113)* | *–.054 (–0.060 to –0.048)* | *–.156 (–0.168 to –0.144)* | *.799 (0.779 to 0.819)* |
|  |  | Treatment | *.158 (0.147 to 0.169)* | *–.050 (–0.056 to –0.044)* | *–.208 (–0.221 to –0.196)* | *.812 (0.791 to 0.834)* |
| **BMI (kg/m^2^) group** | | | | | | |
|  | **Normal** | | | | | |
|  |  | Hypertension | *.086 (0.077 to 0.095)* | *.051 (0.045 to 0.056)* | *–.035 (–0.046 to –0.025)* | *1.226 (1.198 to 1.254)* |
|  |  | Treatment | *.115 (0.106 to 0.124)* | *.055 (0.049 to 0.061)* | *–.060 (–0.071 to –0.050)* | *1.247 (1.219 to 1.276)* |
|  | **Overweight to obese** | | | | | |
|  |  | Hypertension | *.093 (0.086 to 0.100)* | *.016 (0.012 to 0.020)* | *–.077 (–0.085 to –0.069)* | *1.067 (1.050 to 1.084)* |
|  |  | Treatment | *.133 (0.126 to 0.140)* | *.018 (0.014 to 0.022)* | *–.115 (–0.123 to –0.107)* | *1.075 (1.058 to 1.093)* |
| **Depression status** | | | | | | |
|  | **No** | | | | | |
|  |  | Hypertension | *.126 (0.121 to 0.132)* | *.024 (0.021 to 0.028)* | *–.102 (–0.109 to –0.096)* | *1.102 (1.086 to 1.117)* |
|  |  | Treatment | *.162 (0.156 to 0.168)* | *.027 (0.023 to 0.030)* | *–.135 (–0.142 to –0.128)* | *1.113 (1.098 to 1.130)* |
|  | **Yes** | | | | | |
|  |  | Hypertension | *.100 (0.081 to 0.118)* | .007 (–0.004 to 0.019) | *–.092 (–0.114 to –0.071)* | 1.030 (0.983 to 1.078) |
|  |  | Treatment | *.134 (0.115 to 0.154)* | .009 (–0.002 to 0.021) | *–.125 (–0.148 to –0.102)* | 1.039 (0.991 to 1.088) |
| **Educational background** | | | | | | |
|  | **High school or less** | | | | | |
|  |  | Hypertension | *.181 (0.175 to 0.187)* | *.024 (0.021 to 0.028)* | *–.157 (–0.164 to –0.150)* | *1.102 (1.086 to 1.119)* |
|  |  | Treatment | *.207 (0.201 to 0.213)* | *.027 (0.024 to 0.031)* | *–.179 (–0.187 to –0.172)* | *1.116 (1.099 to 1.134)* |
|  | **College or more** | | | | | |
|  |  | Hypertension | *.130 (0.119 to 0.141)* | *.043 (0.037 to 0.049)* | *–.087 (–0.100 to –0.075)* | *1.188 (1.158 to 1.220)* |
|  |  | Treatment | *.212 (0.200 to 0.224)* | *.046 (0.040 to 0.053)* | *–.165 (–0.179 to –0.152)* | *1.204 (1.172 to 1.237)* |
| **Occupational status** | | | | | | |
|  | **White-collar** | | | | | |
|  |  | Hypertension | *.090 (0.076 to 0.104)* | *.032 (0.024 to 0.041)* | *–.057 (–0.073 to –0.041)* | *1.139 (1.101 to 1.179)* |
|  |  | Treatment | *.169 (0.154 to 0.185)* | *.034 (0.025 to 0.043)* | *–.135 (–0.153 to –0.117)* | *1.147 (1.107 to 1.188)* |
|  | **Blue-collar** | | | | | |
|  |  | Hypertension | *.158 (0.150 to 0.166)* | *.028 (0.023 to 0.033)* | *–.130 (–0.140 to –0.121)* | *1.118 (1.096 to 1.141)* |
|  |  | Treatment | *.201 (0.193 to 0.210)* | *.031 (0.026 to 0.036)* | *–.171 (–0.181 to –0.161)* | *1.131 (1.108 to 1.154)* |
|  | **Unemployed** | | | | | |
|  |  | Hypertension | *.137 (0.129 to 0.145)* | *.019 (0.014 to 0.024)* | *–.118 (–0.127 to –0.109)* | *1.080 (1.059 to 1.101)* |
|  |  | Treatment | *.157 (0.149 to 0.165)* | *.022 (0.017 to 0.027)* | *–.135 (–0.144 to –0.125)* | *1.093 (1.071 to 1.114)* |
| **Marital status** | | | | | | |
|  | **Married** | | | | | |
|  |  | Hypertension | *.135 (0.128 to 0.141)* | *.027 (0.022 to 0.031)* | *–.108 (–0.116 to –0.100)* | *1.112 (1.094 to 1.130)* |
|  |  | Treatment | *.171 (0.164 to 0.178)* | *.029 (0.025 to 0.034)* | *–.142 (–0.150 to –0.134)* | *1.125 (1.107 to 1.144)* |
|  | **Unmarried** | | | | | |
|  |  | Hypertension | *.114 (0.104 to 0.124)* | *.025 (0.019 to 0.031)* | *–.089 (–0.100 to –0.077)* | *1.107 (1.081 to 1.134)* |
|  |  | Treatment | *.147 (0.137 to 0.157)* | *.027 (0.021 to 0.033)* | *–.120 (–0.131 to –0.108)* | *1.116 (1.090 to 1.143)* |

^a^Significant difference is indicated in italics (*P*<.05).

^b^Odds ratios during the pandemic period (2020-2022) were compared with those before the pandemic period (2018-2019; the reference value). Covariates in the models were age, sex, region of residence, receipt of basic livelihood security, economic level of the family, smoking status, frequency of alcohol consumption, BMI group, depression status, educational background, occupation status, and marital status.

^c^The economic level of the family was categorized based on monthly household income (see text for details).
